# Red-Light Photoredox C–H Alkylation of Acceptor Heterocycles Enabled by Substoichiometric NADH

**DOI:** 10.1021/acs.orglett.6c01113

**Published:** 2026-04-21

**Authors:** Uxía Deus-Lorenzo, Riccardo Di Forti, Alejandro Cadranel, María Tomás-Gamasa, José Luis Mascareñas, Mauro Mato

**Affiliations:** † Centro Singular de Investigación en Química Biolóxica e Materiais Moleculares (CiQUS) and Departamento de Química Orgánica, 16780 Universidade de Santiago de Compostela, 15705 Santiago de Compostela, Spain; ‡ Department Chemie und Pharmazie, Physikalische Chemie I, 9171 Friedrich-Alexander-Universität Erlangen-Nürnberg (FAU), Egerlandstraße 3, 91058 Erlangen, Germany; § Interdisciplinary Center for Molecular Materials, Friedrich-Alexander-Universität Erlangen-Nürnberg (FAU), Egerlandstraße 3, 91058 Erlangen, Germany; ∥ Departamento de Química Inorgánica, Analítica y Química Física, Facultad de Ciencias Exactas y Naturales, Universidad de Buenos Aires, Pabellón 2, Ciudad Universitaria, C1428EHA Buenos Aires, Argentina; ⊥ Instituto de Química Física de Materiales, Medio Ambiente y Energía (INQUIMAE), CONICET−Universidad de Buenos Aires, Pabellón 2, Ciudad Universitaria, C1428EHA Buenos Aires, Argentina

## Abstract

NADH is a key redox mediator in biology and biocatalysis, yet its catalytic use in non-enzymatic synthetic chemistry remains largely unexplored. Here, we show that NADH can act as a substoichiometric reductive quencher in red-light photoredox catalysis, enabling a redox-neutral C­(sp^2^)–H alkylation of acceptor heterocycles. This strategy reduces waste and leads to excellent yields and selectivity by suppressing overreduction. The reaction proceeds in air, under mild, aqueous-compatible conditions and operates in biorelevant media, establishing NADH as a cofactor for artificial red-light photoredox catalysis.

## Graphical abstract

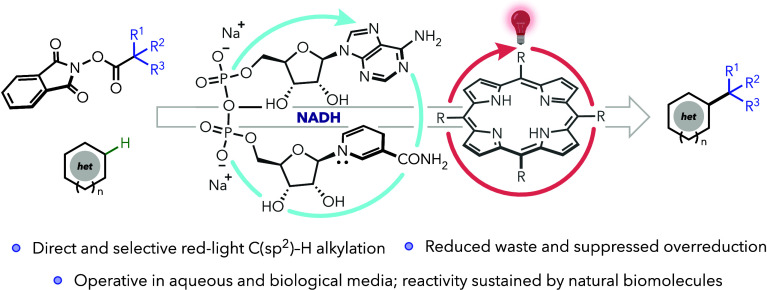

Nicotinamide adenine dinucleotide (NADH) is a universal biological redox cofactor that mediates essential electron-transfer events across central metabolic pathways.^1^ A similar cofactor, NADPH, plays a related role in photosynthesis by transferring reducing power to downstream biochemical processes.^2^ The reversible cycling of the NAD­(P)­H/NAD­(P)^+^ couple enables their repeated participation in net-reductive transformations promoted by dehydrogenases (Figure 1A).^3^ This also extends to redox-neutral processes, such as ketoreductase-promoted isomerizations, in which NAD­(P)H formally acts as a catalytic redox mediator.^4^ Despite these pivotal roles in nature and while NADH has been exploited as a reagent for Giese reactions,^5^ the substoichiometric use of this bioreductant in photoredox catalysis remains essentially unknown.^6^ We envisioned that NADH could be used in catalytic amounts as a biocompatible^7^ reductive quencher of otherwise unreactive red-light-responsive photocatalysts^8^ while maintaining a redox-neutral regime (Figure 1B). Indeed, here, we demonstrate that substoichiometric NADH enables a selective red-light-driven C­(sp^2^)–H alkylation of azauracils and other heterocycles (Figure 1C). In contrast to previous strategies to promote this transformation based on blue-light irradiation,^9^ electrochemistry,^10^ or transition-metal catalysis,^11^ this method operates with low-energy light^12^ and under air, tolerates aqueous or biorelevant media, and allows biomolecules to participate as redox partners. Beyond its conceptual novelty, this strategy inherently reduces waste and minimizes undesired overreduction.

**Figure 1:**
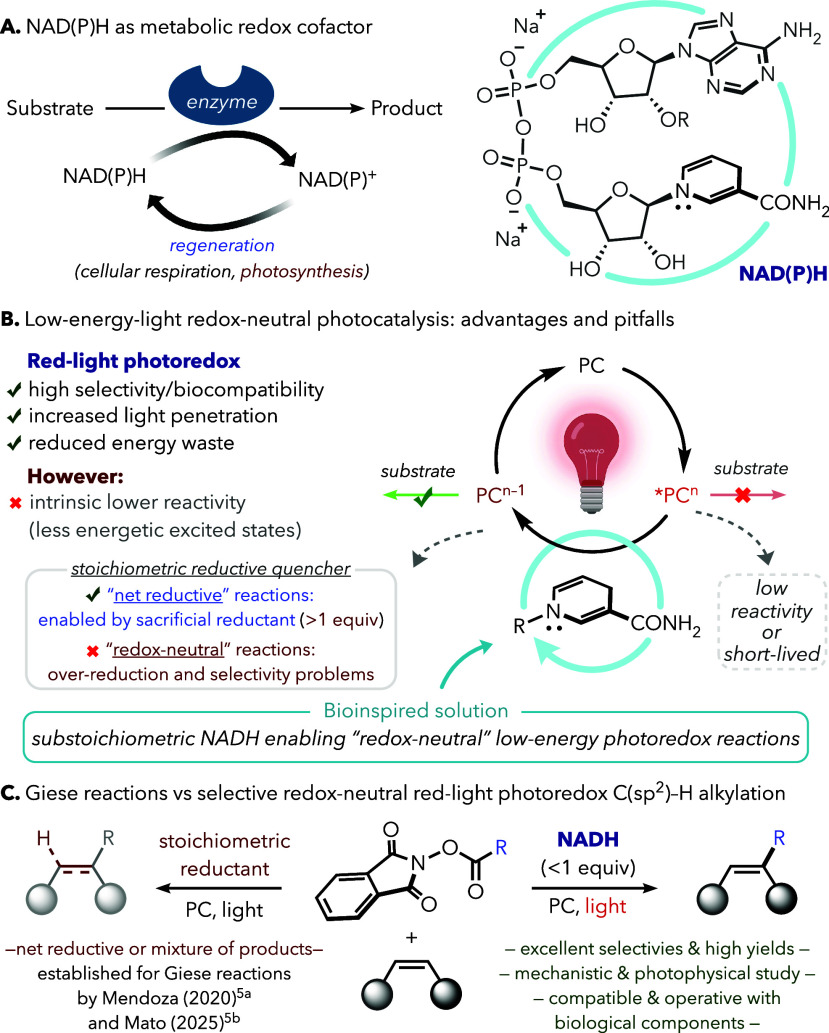
(A) NAD­(P)H as a redox cofactor in enzymatic biocatalysis. (B) Key advantages and drawbacks of low-energy light photoredox catalysis. (C) Classical Giese-type reductive reactivity vs our hypothesis: substoichiometric NADH enables low-energy light to promote redox-neutral C­(sp^2^)–H alkylation.

To evaluate this strategy, we selected redox-active phthalimide esters (RAEs) **1** as model alkyl radical sources,^13^ since they are not oxidizing enough (*E*_p/2_ ≈ −1.5 vs SCE)^14^ to undergo single-electron transfer (SET) from excited-state tetraphenylporphyrin **6** (*E*_ox_* [TPP^•^ ^+^/^3^TPP] = – 0.42 V vs SCE),^15^ an established red-light photocatalyst that has yet to be applied in this type of approach.^16^

Indeed, a mixture of RAE **1a** (1.0 equiv), azauracil **2a** (a model alkyl radical acceptor, 2.0 equiv),^9–11^ and TPP **6** (10 mol %) in DMSO (25 mM) showed no reactivity under red-light (660 nm) irradiation (Table 1, entry 1). In contrast, the addition of 150 mol % BNAH (a synthetic analogue of NADH)^17^ enabled activation of **1a**, producing C–H alkylation product **3a** in 64% yield, together with the Giese-type hydroalkylation product **4a** (3:1 mixture, entry 2), which is the main compound obtained under violet-light HAT-photocatalysis conditions.^18^ Importantly, reducing BNAH loading to only 10 mol % afforded exclusively (>20:1) the redox-neutral product **3a** in an excellent yield (entry 3), likely due to the absence of a sufficient amount of hydride source. The same reactivity was achieved using natural cofactors NADH or NADPH instead of BNAH (entries 4 and 5), highlighting the compatibility of this photoredox system with endogenous nicotinamides. As expected, control experiments without light or TPP gave no reaction (entries 6 and 7). A green-light manifold showed analogous behavior: while stoichiometric BNAH again promoted overreduction (entry 9), using 10 mol % Eosin Y (**5**) and 10 mol % BNAH led to 80% yield and >20:1 **3a**/**4a** selectivity after 1 h of irradiation (550 nm, entry 10). With the Eosin Y manifold, we observed the formation of 18% of **3a** even without BNAH, consistent with the use of a higher energy light system (entry 8). Furthermore, since BNAH is photoactive by itself under blue light,^5a^ we also evaluated the reaction at 456 nm without a photocatalyst (entries 13–15). However, this led only to stoichiometric reactivity (21% yield with 20 mol % BNAH), without significant turnover or propagation within 1 h (see Figure 6 for details).

**Table 1:**
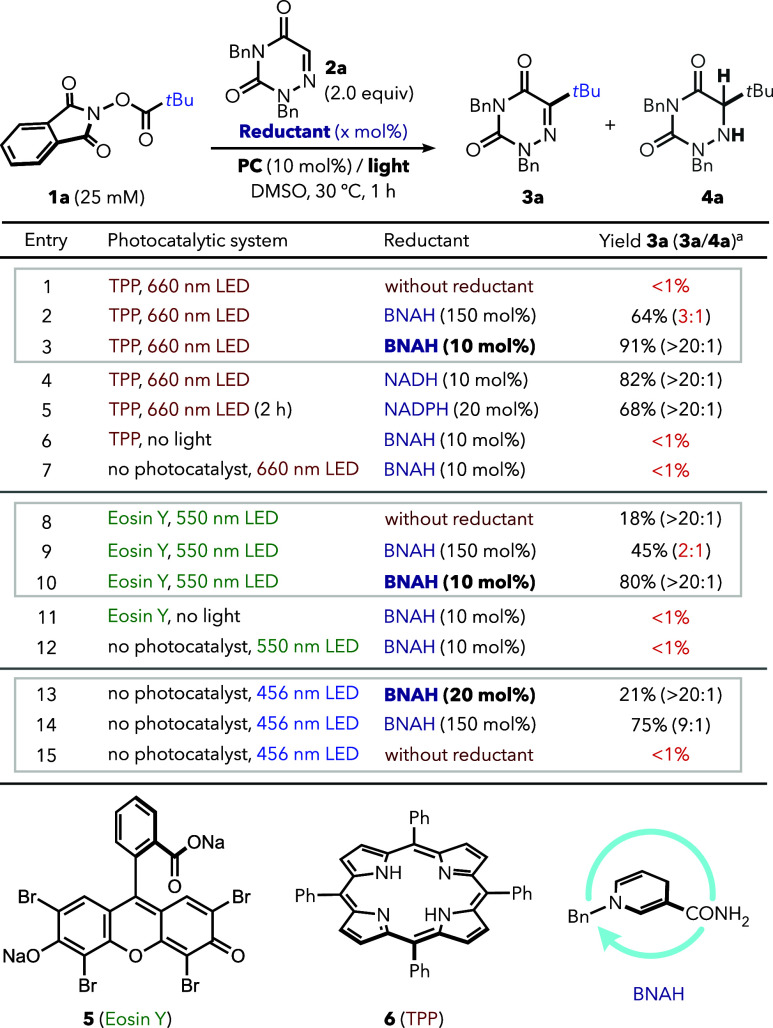
Comparison between Substoichiometric vs Stoichiometric Amounts of Reductants with Different Photocatalytic Manifolds^a^

* Standard conditions: **1a** (0.025 mmol, 25 mM in DMSO under air), **2a** (2.0 equiv), and the corresponding photocatalyst (10 mol %) and reductant (10 mol %) were dissolved in DMSO and submitted to light irradiation for 1 h (2× Kessil LED).

a Yields of **3a** were determined by calibrated GC–MS. **3a**/**4a** ratios are the relative integrals of both products.

After this initial screening, we tested the robustness of our air-tolerant methodology and its compatibility with aqueous and biorelevant conditions (Figure 2A). Thus, we switched to 1:1 water/DMSO as the solvent mixture, using 2 mol % tetrakis­(4-carboxyphenyl)­porphyrin (TCPP, **7**) instead of water-insoluble TPP. Under these conditions, efficient formation of **3a** was observed in the presence of NADH or NADPH (20 mol %, 92–95% yield), while no product was detected without a reductant. We then replaced water by Dulbecco’s Modified Eagle’s Medium (DMEM), a common cell culture medium containing a variety of biomolecules. The NADH-promoted reaction operated well within this complex medium, giving 79% **3a**. Remarkably, when we tested the reaction in DMEM without NADH, we still observed a 46% yield of **3a**, indicating that biomolecules present in this medium can act as reductive quenchers. HeLa cell lysates (3 mg/mL) also supported this reactivity: in the absence of added NADH, **3a** was obtained in 65% yield at 1 mM, whereas supplementation of the cell lysate medium with 10 mol % NADH increased the yield to 94% even at 10 mM. These results indicate that endogenous bioreductants may sustain the photoredox manifold under biologically relevant conditions.^7^ Beyond the use of natural bioreductants, we also examined whether we could harness the photocatalytic activity of natural pigments found in photosynthetic organisms to promote this artificial photoredox process. For this purpose, we tested the reaction using a crude spinach extract in DMSO as solvent (Figure 2B).^19^ This gave 26% of **3a** without an added photocatalyst, while controls lacking light, NADH, or plant extract led to no reactivity. Although preliminary, this result serves as a proof of concept for compatibility with natural pigments.

**Figure 2:**
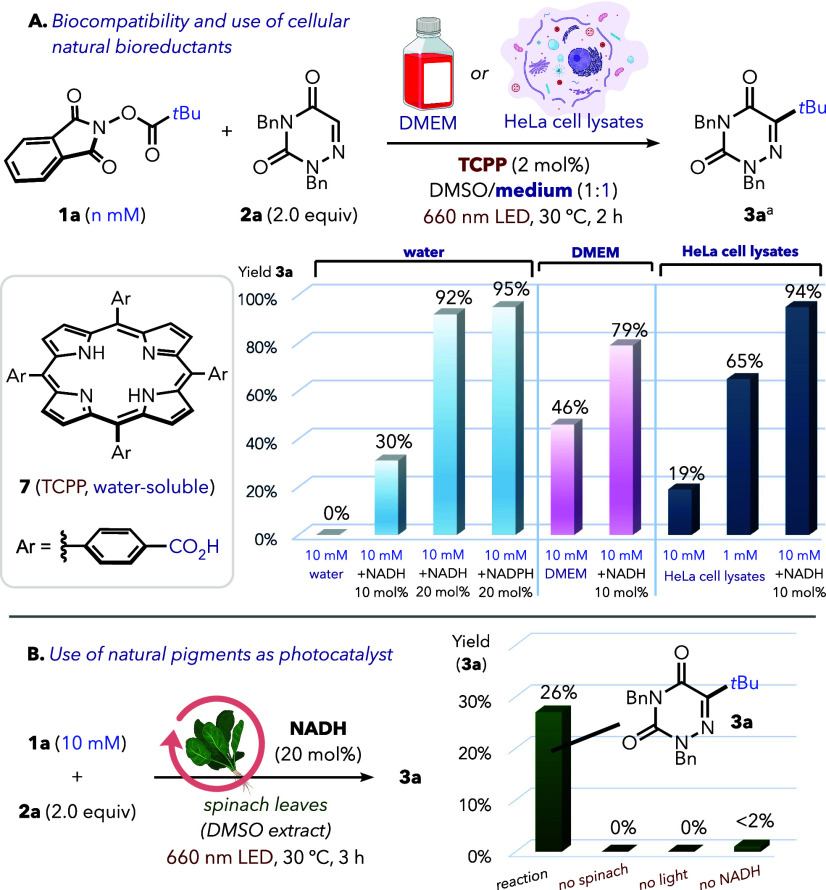
(A). Biocompatible C–H alkylation operates using natural biomolecules as reductive quenchers. (B) Reaction using natural pigments from spinach leaves as a photocatalyst. Yields of **3a** were determined by calibrated GC–MS as an average of two replicates. For detailed experimental conditions, see the Supporting Information.

Subsequently, we evaluated the reaction scope under standard conditions in DMSO with TPP as the photocatalyst (Figure 3). For all substrates, isolated yields were determined on a 0.20 mmol scale using BNAH (10 mol %), while reactions with NADH (10 mol %) were conducted on a 0.025 mmol scale (^1^H NMR yield). First, we tested a variety of RAEs **1** using azauracil **2a** as a model substrate. The reaction tolerated a wide range of RAEs, enabling transfer of tertiary (**3a**–**3d**), secondary (**3e** and **3f**), and even primary (**3g** and **3h**) alkyl radicals in good to excellent yields and excellent selectivity toward the unsaturated products. Notably, the reaction scaled to 1.0 mmol without further optimization, giving **3a** in 86% yield. A range of azauracil derivatives presenting functional groups, such as different amides (**3i**), unprotected N–H bonds (**3j**), esters (**3k**), or terminal alkynes (**3l**), also reacted smoothly. In contrast, the reaction of nonaza uracil **2m** afforded only trace amounts of **3m**, highlighting the importance of using an acceptor partner that can generate a reducing α-amino radical after addition (see **10** in Figure 5A and Figure S5).^11b^ Importantly, the selective redox-neutral C­(sp^2^)–H alkylation can also be extended to other heterocycles. We obtained products of alkylation of six-membered heterocycles, such as quinoxaline (**3o**) or quinoxalinone (**3p**), and the five-membered ring of indazole, confirmed by X-ray diffraction analysis of **3n**. Overall, both NADH and BNAH performed similarly across the entire scope (for details on scope limitations, see Figure S5). Finally, we further confirmed the benefits of using a substoichiometric reductive quencher beyond reducing waste, through the alkylation of chromone, a typical Giese-type acceptor.^20^ Using 20 mol % BNAH or NADH resulted in 6:1 or >20:1 selectivity, respectively, favoring unsaturated product **3q** over Giese-type reduced derivative **4q**. In contrast, stoichiometric amounts of either reductant led to a significant decrease in the overall yield, with a higher amount of the Giese product (1:5–1:2 **3q**/**4q** ratio) formed through HAT.

**Figure 3:**
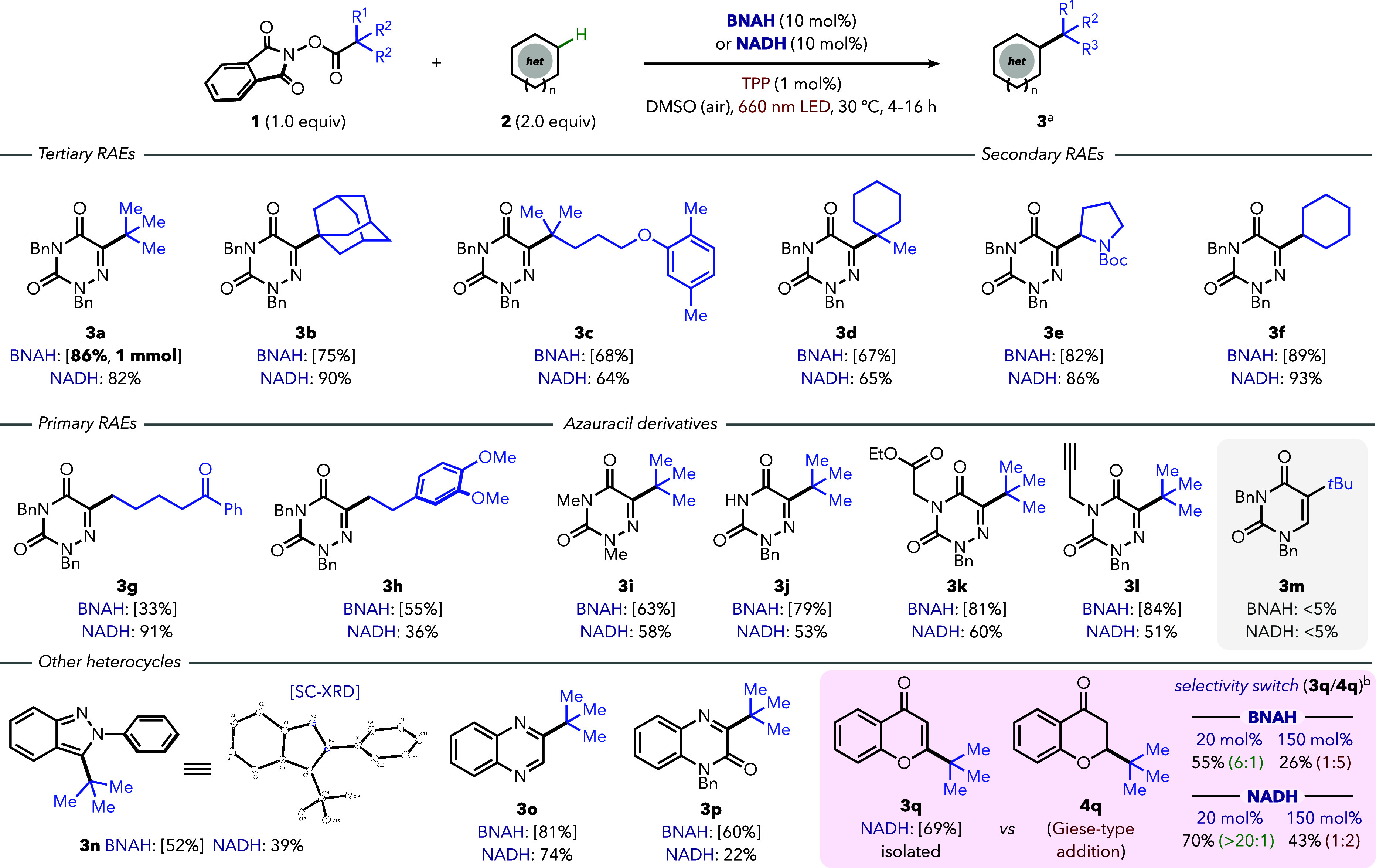
Reaction scope. With BNAH, isolated yields in parentheses at the 0.20 mmol scale; with NADH, yields of **3** determined by ^1^H NMR at the 0.025 mmol scale. ^a^Standard conditions for isolated yields: **1** (0.20 mmol, 50 mM in DMSO under air), **2** (2.0 equiv), BNAH (10 mol %), and **6** (TPP, 1 mol %) were submitted to red-light (2× 660 nm Kessil LED) irradiation for 4–16 h. ^b^With 5 mol % TPP (**6**) and Cs_2_CO_3_ (2.0 equiv), with the **3r**/**4r** ratio in parentheses determined by ^1^H NMR [isolated yield at the 0.20 mmol scale with NADH (20 mol %) instead of BNAH]. Ellipsoids at 50% probability for X-ray data (**3n**).

We then performed a preliminary kinetic study to determine the effects of the different key reaction components. First, we confirmed a positive effect of increasing BNAH loading in the 0–8 mol % range (Figure 4A), observing >40% yield after 1 h with only 2 mol % BNAH. A similar scenario was observed when varying the loading of photocatalyst (TPP) between 0 and 1 mol % at a constant 2.5 mol % BNAH (Figure 4B). This positive effect plateaus above 1 mol %, where the experimental solubility limit of TPP (**6**) in DMSO is reached. Finally, we found that the red-light intensity displayed a clear linear influence on the reaction rate for both continuous illumination (Figure 4C) and intermittent irradiation (Figure 4D), reinforcing that productive turnover is directly tied to photon input and that the reaction stops upon light interruption.

**Figure 4:**
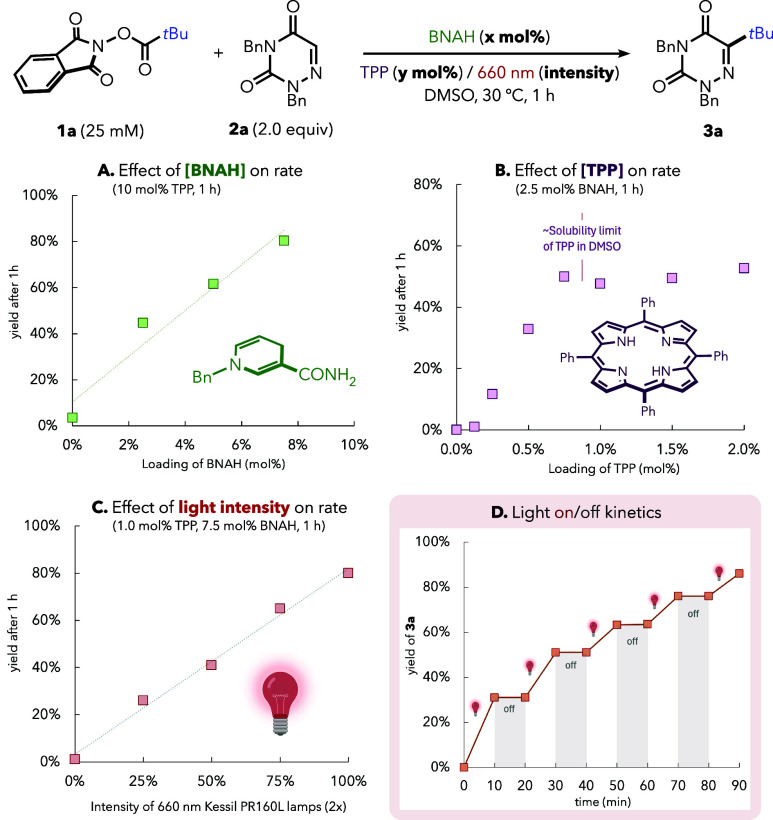
Effect on the rate of the different reaction components: (A) BNAH loading, (B) TPP loading, and (C) light intensity. (D) Intermittent irradiation kinetics. Yields of **3a** were determined by calibrated GC–MS. For experimental details, see the Supporting Information.

Our mechanistic hypothesis relies on the reductive quenching of triplet TPP by BNAH/NADH (Figure 5A). To investigate this process, we performed nanosecond transient absorption spectroscopy (nsTAS). TPP in DMSO displayed expected porphyrin dynamics: a TPP^S1^ singlet state that decays in ∼10 ns to populate triplet TPP^T1^, which returns to the ground state in ∼50 μs (Figure S9). In the presence of BNAH, the lifetime of TPP^T1^ is reduced to ∼10 μs and a longer lived species (∼50 μs) appears, with spectral features characteristic of TPP^•^ ^–^, consistent with the formation of a {TPP^•^ ^–^·BNAH^•^ ^+^} charge-separated state (CSS) upon SET (Figure 5B and Figure S10).^21^ Stern–Volmer studies (Figure 5C and Figure S12) revealed efficient quenching of both TPP^S1^ and TPP^T1^ by BNAH, with the former near the diffusion limit (*k*_q_ = 1.1 × 10^9^ M^–1^ s^–1^) and the latter reversible, whereas RAE **1a** showed no measurable quenching of either excited state.

**Figure 5:**
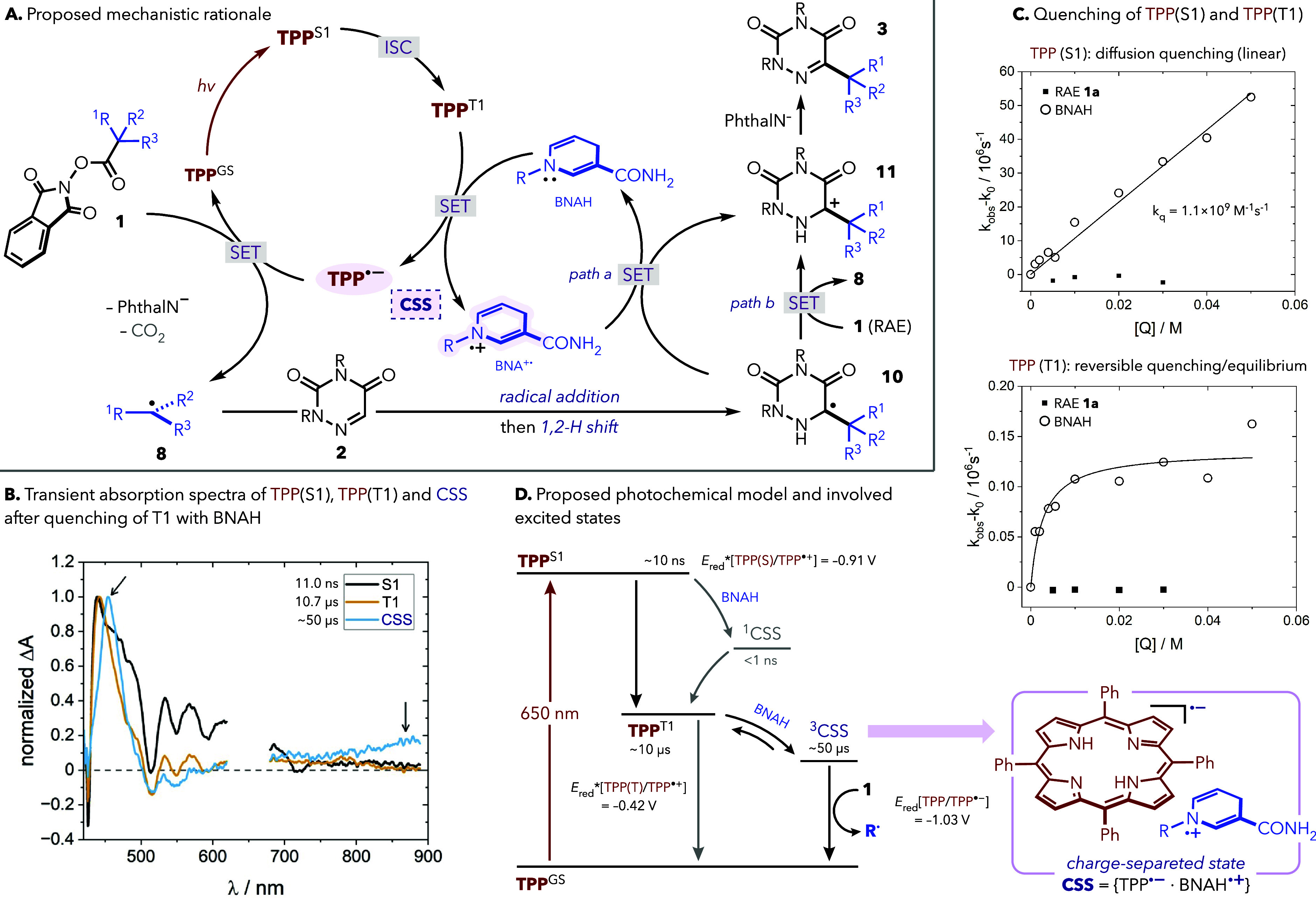
Mechanistic rationale and TAS. (A) Proposed reaction mechanism and possible pathways based on the use of substoichiometric BNAH/NADH. (B) Species-associated differential spectra were derived from TAS of TPP in the presence of 4 mM BNAH in DMSO at room temperature under 650 nm excitation. (C) Stern–Volmer quenching experiments of the singlet and triplet excited states of TPP (**6**) with RAE **1a** or BNAH. (D) Proposed photochemical model.

A similar behavior was observed when using NADH instead of BNAH (Figure S11): the CSS/TPP^T1^ lifetimes and the quenching constants are on the same order of magnitude, indicating that BNAH is an excellent model for NADH under the conditions explored. These data support the reductive-quenching hypothesis and explain the lack of redox-neutral reactivity in the absence of BNAH/NADH (Table 1, entry 1). When BNAH (4 mM) was present, the addition of RAE **1a** altered the dynamics only at long time delays (∼100 μs), while the TPP^S1^ and TPP^T1^ dynamics remained unchanged (Figure S14). This is consistent with a bimolecular reaction between RAE and the CSS. Together with the known redox potentials of TPP,^15^ this study supports the photochemical model shown in Figure 5D, in which activation of RAEs proceeds via SET reduction from TPP^•^ ^–^ generated through reductive quenching (for more details, see the Supporting Information). After SET reduction and fragmentation of **1a**, released alkyl radical **8** is trapped by azauracil **2** to give a N-centered radical, which then undergoes a fast 1,2-H shift to form more stable C-centered radical **10**.^9–11^ Then, **10** evolves to redox-neutral product **3** through SET oxidation to give **11**, followed by elimination. The oxidation of highly reducing α-amino radical **10** can proceed through two feasible pathways that would allow turnover in the presence of substoichiometric BNAH (or NADH). First, transient BNAH^•^ ^+^ or BNA^+^ generated upon SET oxidation could serve as an oxidant, thereby regenerating a reductive quencher (path a). Alternatively, intermediate **10** could reduce another unit of RAE **1**, initiating a free-radical chain (path b).

Experiments performed without a photocatalyst, relying solely on direct blue-light excitation of BNAH, afforded only stoichiometric amounts of product (Figure 6). Although the role of BNAH cannot be unambiguously established,^22^ these observations suggest that radical initiation alone is insufficient to sustain efficient reactivity and that the presence of the photocatalyst plays a key kinetic role. This is consistent with a scenario in which the interaction of BNAH/NADH with TPP either enables catalytic turnover or accelerates the initiation of radical chains.

**Figure 6:**
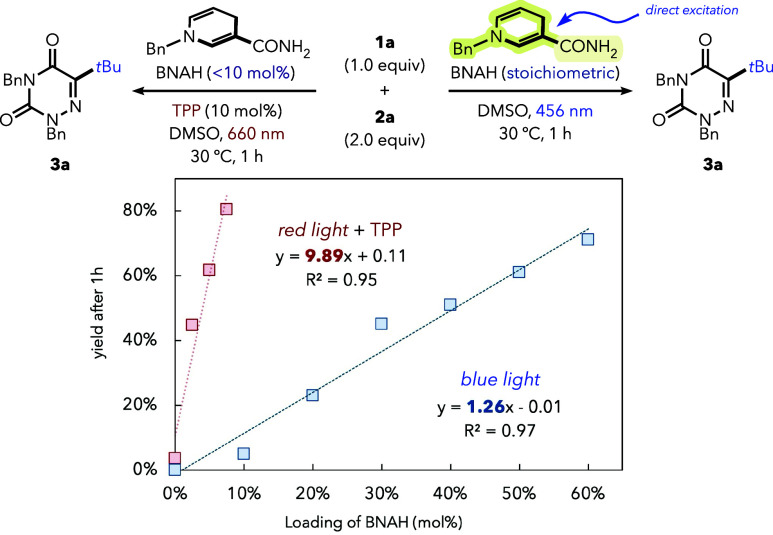
Effect of BNAH loading in red-light photocatalysis versus direct excitation under blue-light irradiation. Yields of **3a** were determined by calibrated GC–MS. For detailed experimental conditions, see the Supporting Information.

In summary, we have developed a red-light photoredox platform for redox-neutral C­(sp^2^)–H alkylation based on the use of substoichiometric amounts of natural cofactor NADH as a reductive quencher. Operating within a low-energy, redox-neutral regime, this approach enables the selective activation of challenging substrates while minimizing overreduction and avoiding stoichiometric waste. The method operates under air, in aqueous media, and in biorelevant environments. Kinetic and spectroscopic TAS data are consistent with a reductive-quenching mechanism involving a TPP–NADH charge-separated state. Collectively, these findings establish natural NADH as a cofactor for mild, red-light-driven artificial C–H functionalization, providing a basis for future exploration of photoredox processes under biological conditions.

## Supplementary Material



## Data Availability

The data underlying this study are available in the published article and its Supporting Information.

## Notes

Additional experimental details, materials, methods, and full characterization data (^1^H, ^13^C, HRMS, and X-ray) for all new compounds (PDF)
