# Supplementary material for: Red-Light Photoredox C–H Alkylation of Acceptor Heterocycles Enabled by Substoichiometric NADH
Source: Org Lett. 2026 Apr 21;28(17):5508–14. doi: 10.1021/acs.orglett.6c01113 (PMC13140127; doi:10.1021/acs.orglett.6c01113)
Supplement: Supplementary file 1 [file ol6c01113_si_001.pdf]

Supporting Information

Red-Light Photoredox C–H Alkylation of Acceptor Heterocycles Enabled by Substoichiometric NADH

Uxía Deus-Lorenzo,[a] Riccardo Di Forti,[b,c] Alejandro Cadranel,[b,c,d] María Tomás-Gamasa,[a,] José L. Mascareñas,[a,]* Mauro Mato[a,]*

* Email: mauro.mato@usc.es, joseluis.mascarenas@usc.es

[a] Centro Singular de Investigación en Química Biolóxica e Materiais Moleculares (CiQUS) and Departamento de Química Orgánica, Universidade de Santiago de Compostela, 15705, Santiago de Compostela (Spain).

[b] Department Chemie und Pharmazie, Physikalische Chemie I, Friedrich-Alexander-Universität Erlangen-Nürnberg, Egerlandstraße 3, 91058 Erlangen, Germany.

[c] Friedrich-Alexander-Universität Erlangen-Nürnberg (FAU), Interdisciplinary Center for Molecular Materials, Egerlandstraße 3, 91058, Erlangen, Germany.

[d] Universidad de Buenos Aires, Facultad de Ciencias Exactas y Naturales, Departamento de Química Inorgánica, Analítica y Química Física, Pabellón 2, Ciudad Universitaria, C1428EHA, Buenos Aires, Argentina. CONICET – Universidad de Buenos Aires. Instituto de Química Física de Materiales, Medio Ambiente y Energía (INQUIMAE), Pabellón 2, Ciudad Universitaria, C1428EHA, Buenos Aires, Argentina.

Supporting Information

1. General considerations 3

2. Light sources and photochemical set-up 4

   2.1. Light sources (Kessil LED) 4

   2.2. Photochemical reactions set-up 5

3. Synthesis of redox-active esters 7

   3.1. General procedure A for the synthesis of alkyl redox-active esters **1** 7

   3.2. Characterization data for alkyl redox-active esters **1** 8

4. Synthesis of C(sp^2)–H reaction substrates **2** 12

   4.1. General procedure B for the *N*-alkylation of reaction substrates **2** 12

   4.2. Detailed reaction conditions and characterization data for the substrates **2** 13

5. Redox-neutral C–H alkylation with redox-active esters 16

   5.1. General procedure C for red-light-promoted alkylation reactions at small scale 16

   5.2. General procedure D for the scale-up and isolation of photochemical reactions 17

   5.3. Detailed reaction conditions and characterization data for products **3** 18

   5.4. Unsuccessful substrates and scope limitations 34

6. Reaction development, optimization and control experiments 35

7. Biocompatible reactions using bioavailable components 41

   7.1. Reactions using bioreductant-containing media 41

   7.2. Reactions using natural-pigment extracts from spinach leaves 42

8. Photophysical studies 44

   8.1. UV–Vis absorption spectroscopy 44

   8.2. Transient-absorption spectroscopy (TAS) 45

9. Mechanistic experiments 52

   9.1. Effect of reaction components 52

   9.2. Red-light photocatalysis vs blue-light direct excitation 57

11. Crystal data and structure refinement 59

11. NMR spectra 65

12. References 95

1. General considerations

Unless stated otherwise, all reactions were conducted under air in HPLC-grade solvents. The water used in the reactions was deionized and purified on a Millipore Milli-Q® Integral system. Dry solvents (if needed) were directly purchased from Sigma–Aldrich and used without further purification. Unless otherwise noted, all reagents were obtained from commercial suppliers and used without further purification. Phosphate buffered saline (PBS, pH 7.4) was prepared following standard procedures. Dulbecco′s Modified Eagle's Medium (Gibco DMEM) was purchased from ThermoFisher Scientific. HeLa cell lysates were obtained from 2 days cultured HeLa cells: after two washings with PBS, cells were scraped from the well, sonicated and diluted with PBS to reach the indicated concentration.

^{1}H and ^{13}C NMR data were recorded in $CDCl_3$, using a Varian Mercury 300 MHz or Bruker AVIII 500 MHz spectrometer. (at 298–300 K, unless stated otherwise). ^{1}H and ^{13}C chemical shifts (δ) are reported in ppm relative to the solvent residual peaks as internal reference. For ^{1}H NMR, the following residual proton peaks of the deuterated solvents were used: $CDCl_3$, $\delta_H(CHCl_3)$ 7.26. For ^{13}C NMR: $CDCl_3$, δ 77.16. ^{13}C spectra were acquired with broadband ^{1}H decoupling unless mentioned otherwise. Coupling constants (*J*) are provided in Hz, and ^{1}H-NMR multiplicities are reported as follows: chemical shift (δ ppm), integration, multiplicity (s = singlet, d = doublet, t = triplet, q = quartet, dd = double doublet, td = triple doublet, m = multiplet, br = broad). NMR spectra were analyzed using MestReNova NMR data processing software. GC–MS analysis was performed on a 8890 GC System with a 5977B GC/MSD (CI) from Agilent. High-resolution mass spectra (HRMS) were acquired using electrospray ionization (ESI) in a Bruker microTOF (time-of-flight analyzer) instrument in FIA mode (flow-injection analysis). UV–Vis absorption spectra were recorded on a Jasco V-770 spectrometer using either disposable plastic cuvettes (2x10 mm) or quartz cuvettes (10x10 mm).

Thin-layer chromatography (TLC) was performed on pre-coated Merck 60 silica gel F_{254} plates. TLC plates were visualized by observation under UV light and/or staining with either phosphomolybdic acid solution or potassium permanganate solution, followed by heat. Chromatographic purifications were performed by flash column chromatography on silica gel (Merck Geduran® Si 60, 40–63 μm) or by preparative TLC using PLC Silica gel 60 F_{254}, 1 mm, 20x20 cm (Analtech).

Unless stated otherwise, EY, Eosin Y-Na_2 or EY-Na_2 all refer to Eosin Y disodium salt (**5**), and TPP refers to 5,10,15,20-tetraphenylporphyrin (**6**). BNAH/BnNAH refers to 1-benzyl-1,4-dihydro-nicotinamide and NADH refers to nicotinamide adenine dinucleotide.

2. Light sources and photochemical set-up

2.1. Light sources (Kessil LED)

Kessil PR160L

Blue, green and red-light irradiation was performed using standardized 456, 525 and 660 nm LED (respectively) PR160L lamps purchased from Kessil (see next section for details on the photochemical set-up). Unless stated otherwise, the lamps were used at full intensity (100%).

The emission profile of the three light sources was recorded with a MK350S Premium Handheld Spectrometer from UPRtek, sitting around 20 cm in front of the Kessil LED light at 25% intensity (Figure S1).

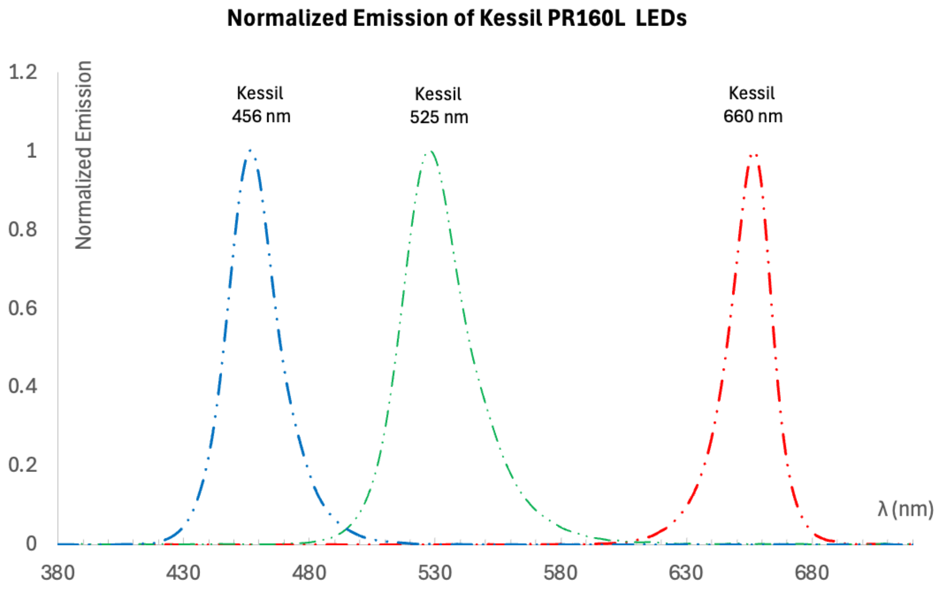

Figure S1. Recorded normalized emission of the Kessil LED light sources employed in this study.

For reference, the irradiance at the maximum emission wavelength for each of the Kessil LEDs employed was measured with the same instrument, for a single Kessil lamp at 25% potency, standing 20 cm away from the UPRtek MK350S Premium detector, giving the following values:

- Kessil PR160L-456 (19V, 40W Max.): λpV = 4008 mW/m^2
- Kessil PR160L-525 (19V, 40W Max.): λpV = 1165 mW/m^2
- Kessil PR160L-660 (19V, 40W Max.): λpV = 3284 mW/m^2

Intensity maps, cross-section illumination areas and more details can be found at the Kessil website:

https://kessil.com/products/science_PR160L.php

2.2. Photochemical reactions set-up

Photochemical reactions were performed inside a 3D-printed reactor box with two Kessil PR160L lamps of the appropriate wavelength (see previous section for details) at full intensity (100%) attached to the chamber and pointed towards the middle. The reactor box is also equipped with an electrical ventilation system, where cooling was provided by two 12 V DC axial fans (92 × 92 × 25 mm, 1.68 W each, model MF92251V1-1000U-A99 from Sunon, situated ca. 5 cm away from the samples) to keep the temperature of the chamber below 35 ºC. The photoreactor was installed on top of a stirring plate in order to stir the reaction mixtures using Teflon-coated magnetic stirring bars.

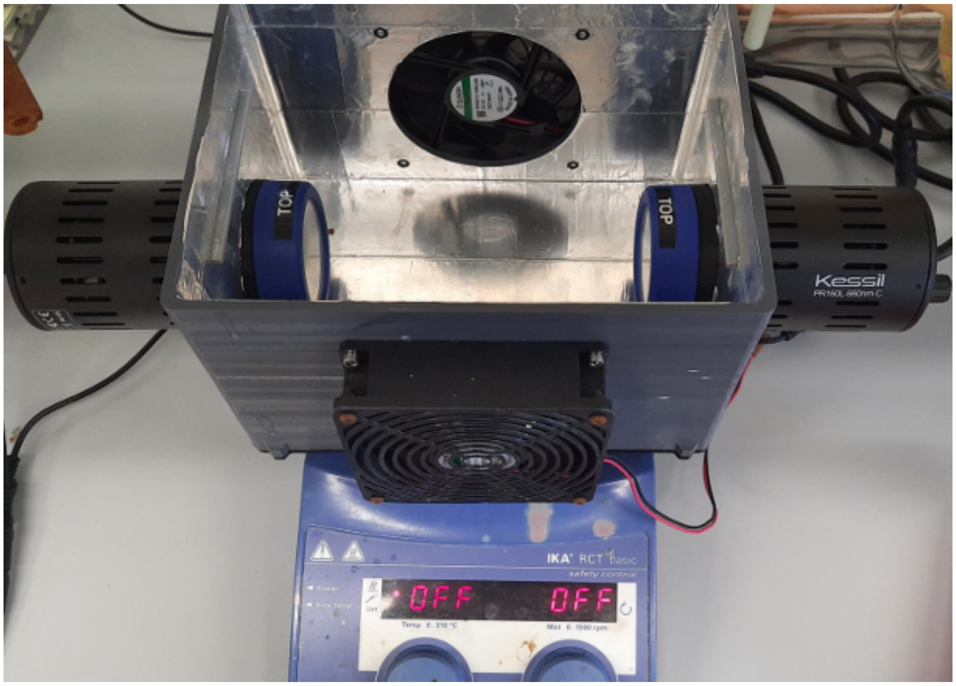

Figure S2. Picture of the photoreactor box employed in this work, equipped with two fans and two Kessil lamps pointing towards the middle.

The reactions were set up in 10 mL screw-cap glass culture tubes under ambient atmosphere, with no special precautions taken to exclude air. The reaction tubes were inserted into a 3D-printed plastic sample holder which left reaction mixtures exposed to direct light irradiation in front of the Kessil lamps (see Figures S3 and S4). A maximum of 4 reaction tubes were irradiated at the same time. Each tube was situated ca. 6 cm away (the standard reference distance used by the LED supplier for their irradiance maps) of the closest light source.

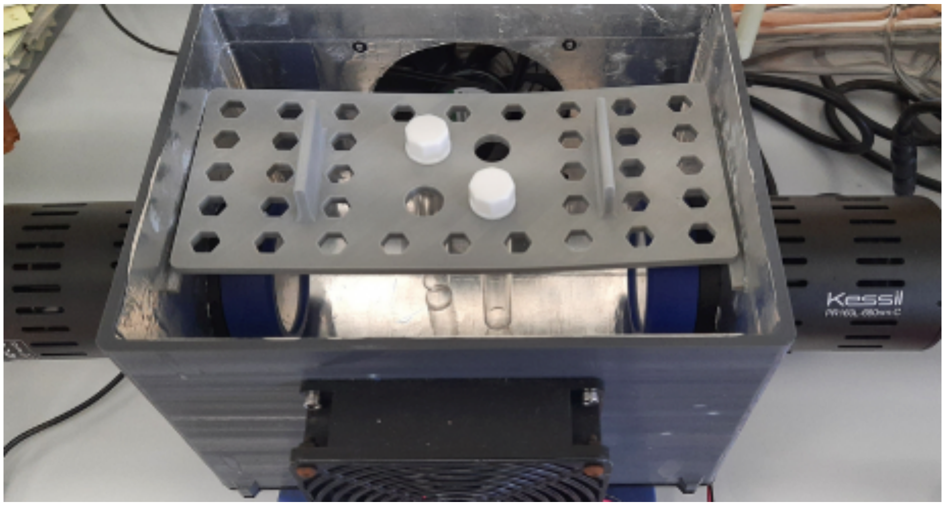

Figure S3. Picture of the photoreactor and sample holder for 10 mL screw-cap glass culture tubes.

The photoreactor can either be left open or further closed with either aluminum foil or a 3D-printed plastic cover.

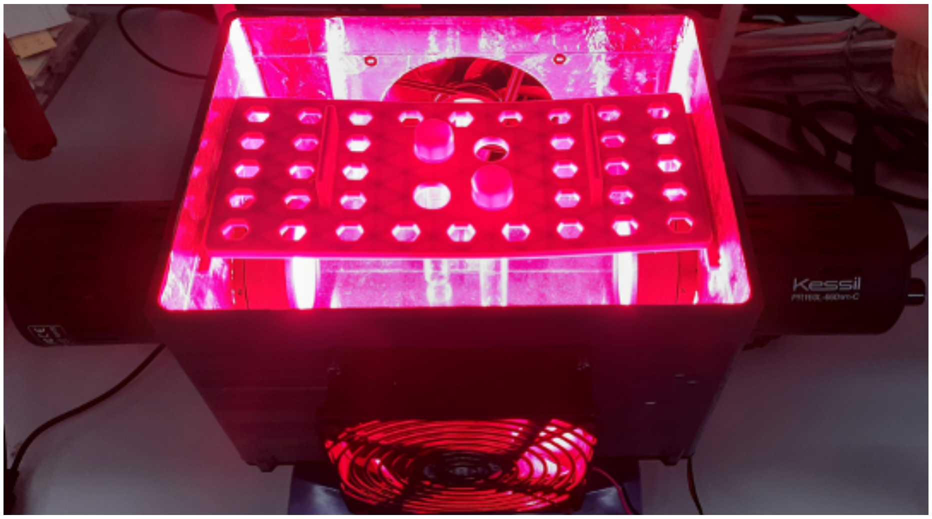

Figure S4. Picture of the photochemical set up with two reaction tubes under red-light irradiation.

3. Synthesis of redox-active esters

3.1. General procedure A for the synthesis of alkyl redox-active esters **1**

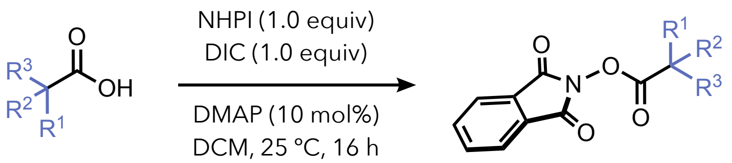

Following typical procedures for the synthesis of alkyl phthalimide esters,[1] a round-bottom flask of the appropriate size (equipped with a Teflon-coated stirring bar) was charged under air atmosphere with the corresponding carboxylic acid (1.0 equiv), *N*-hydroxyphthalimide (1.0 equiv) and 4-dimethylaminopyridine (DMAP, 10 mol%). All reagents were dissolved in HPLC-grade DCM (0.15–0.20 M), before *N,N'*-diisopropylcarbodiimide (DIC, 1.0 equiv) was added in a single portion via syringe. The flask was capped with a rubber septum, and the reaction mixture was left stirring at room temperature overnight (ca. 16 h), until TLC revealed consumption of most of the starting carboxylic acid. After this time, the solvent was removed in vacuum and product **1** was directly purified by flash column chromatography in silica gel, using the appropriate gradients of hexane and EtOAc.

Reaction substrates **1** were prepared from commercially available alkyl carboxylic acids, following General Procedure A. This procedure is based on well-established synthetic methods and, while the used compounds are already known in the literature,[1] for the sake of practicality, exact experimental details and NMR data is reported in the following pages.

3.2. Characterization data for alkyl redox-active esters **1**

1,3-Dioxoisoindolin-2-yl pivalate (1a)

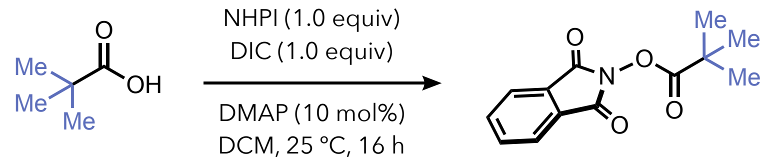

Following General Procedure A, the title product was obtained from pivalic acid (0.15 g, 1.5 mmol, 1.0 equiv), *N*-hydroxyphthalimide (0.25 g, 1.5 mmol, 1.0 equiv), 4-dimethylaminopyridine (DMAP, 18 mg, 0.15 mmol, 10 mol%) and *N,N'*-diisopropylcarbodiimide (DIC, 0.19 g, 0.23 mL, 1.5 mmol, 1.0 equiv) in DCM (7.5 mL, 0.20 M). Purification by flash column chromatography in silica gel, using hexanes/EtOAc as solvent (gradient from 9:1 to 7:3). R_f (7:3 hexane/EtOAc) = 0.7. This gave 0.22 g (78%) of the title product as a yellow solid.

^{1}H NMR (300 MHz, $CDCl_3$) δ 7.90 – 7.85 (m, 2H), 7.80 – 7.75 (m, 2H), 1.43 (s, 9H).

^{13}C NMR (75 MHz, $CDCl_3$) δ 174.5, 162.2, 134.8, 129.3, 124.0, 38.6, 27.2.

1,3-Dioxoisoindolin-2-yl adamantane-1-carboxylate (1b)

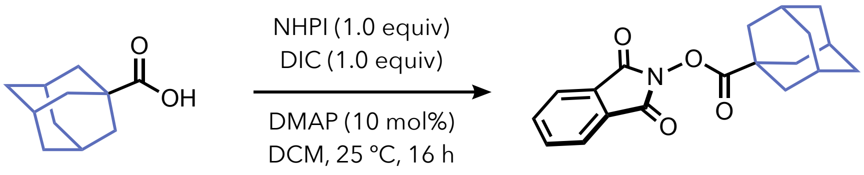

Following General Procedure A, the title product was obtained from 1-adamantanecarboxylic acid (0.72 g, 4.0 mmol, 1.0 equiv), *N*-hydroxyphthalimide (0.65 g, 4.0 mmol, 1.0 equiv), 4-dimethylaminopyridine (DMAP, 49 mg, 0.40 mmol, 10 mol%) and *N,N'*-diisopropylcarbodiimide (DIC, 0.51 g, 0.62 mL, 4.0 mmol, 1.0 equiv) in DCM (20 mL, 0.20 M). Purification by flash column chromatography in silica gel, using hexanes/EtOAc as solvent (gradient from 95:5 to 85:15). R_f (9:1 hexane/EtOAc) = 0.3. This gave 1.10 g (85%) of the title product as a white solid.

^{1}H NMR (300 MHz, $CDCl_3$) δ 7.91 – 7.85 (m, 2H), 7.82 – 7.75 (m, 2H), 2.17 – 2.14 (m, 6H), 2.13 – 2.09 (m, 3H), 1.81 – 1.78 (m, 6H).

^{13}C NMR (75 MHz, $CDCl_3$) δ 173.2, 162.1, 134.6, 129.1, 123.8, 40.6, 38.5, 36.2, 27.7.

1,3-Dioxoisoindolin-2-yl 5-(2,5-dimethylphenoxy)-2,2-dimethylpentanoate (1c)

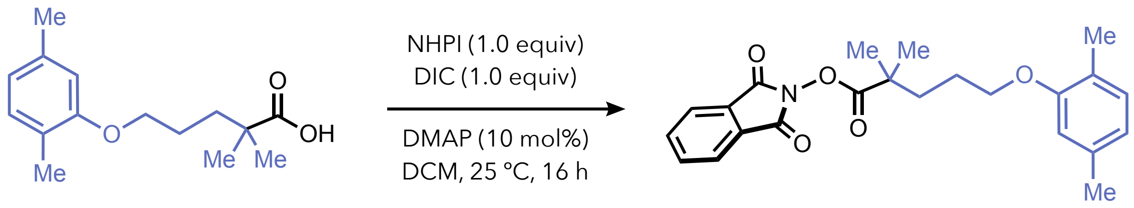

Following General Procedure A, the title product was obtained from gemfibrozil (0.75 g, 3.0 mmol, 1.0 equiv), *N*-hydroxyphthalimide (0.49 g, 3.0 mmol, 1.0 equiv), 4-dimethylaminopyridine (DMAP, 37 mg, 0.50 mmol, 10 mol%) and *N,N'*-diisopropylcarbodiimide (DIC, 0.38 g, 0.46 mL, 5.0 mmol 1.0 equiv) in DCM (15 mL, 0.20 M). Purification by flash column chromatography in silica gel, using hexanes/EtOAc as solvent (gradient from 95:5 to 85:15). R_f (8:2 hexane/EtOAc) = 0.4. This gave 0.93 g (79%) of the title product as a white solid.

^{1}H NMR (300 MHz, $CDCl_3$) δ 7.91 – 7.84 (m, 2H), 7.82 – 7.75 (m, 2H), 7.00 (d, *J* = 8.0 Hz, 1H), 6.68 – 6.63 (m, 2H), 4.01 (m, 2H), 2.32 (s, 3H), 2.19 (s, 3H), 1.99 – 1.93 (m, 4H), 1.45 (s, 6H).

^{13}C NMR (75 MHz, $CDCl_3$) δ 173.9, 162.2, 157.1, 136.6, 134.8, 130.4, 129.2, 124.0, 123.8, 120.9, 112.2, 67.9, 42.1, 37.5, 25.3, 25.1, 21.5, 15.9.

1,3-Dioxoisoindolin-2-yl 1-methylcyclohexane-1-carboxylate (1d)

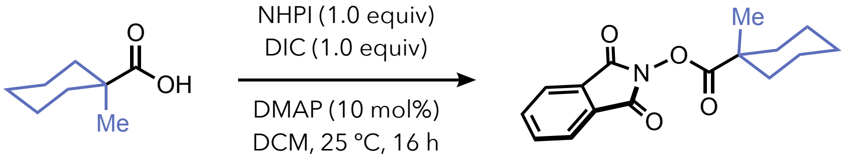

Following General Procedure A, the title product was obtained from 1-methylcyclohexane-1-carboxylic acid (0.71 g, 5.0 mmol, 1.0 equiv), *N*-hydroxyphthalimide (0.82 g, 5.0 mmol, 1.0 equiv), 4-dimethylaminopyridine (DMAP, 61 mg, 0.50 mmol, 10 mol%) and *N,N'*-diisopropylcarbodiimide (DIC, 0.63 g, 0.77 mL, 5.0 mmol 1.0 equiv) in DCM (33 mL, 0.15 M). Purification by flash column chromatography in silica gel, using hexanes/EtOAc as solvent (gradient from 95:5 to 9:1). R_f (9:1 hexane/EtOAc) = 0.4. This gave 1.27 g (88%) of the title product as a colorless oil that solidified upon standing in the fridge to give a white solid.

^{1}H NMR (300 MHz, $CDCl_3$) δ 7.88 (dd, *J* = 5.5, 3.1 Hz, 2H), 7.78 (td, *J* = 5.3, 2.1 Hz, 2H), 2.30 – 2.16 (m, 2H), 1.71 – 1.51 (m, 5H), 1.43 (s, 3H), 1.42 – 1.20 (m, 3H).

^{13}C NMR (75 MHz, $CDCl_3$) δ 173.7, 162.2, 134.6, 129.1, 123.8, 43.2, 35.7, 26.7, 25.5, 23.0.

1-(*tert*-Butyl) 2-(1,3-dioxoisoindolin-2-yl) pyrrolidine-1,2-dicarboxylate (1e)

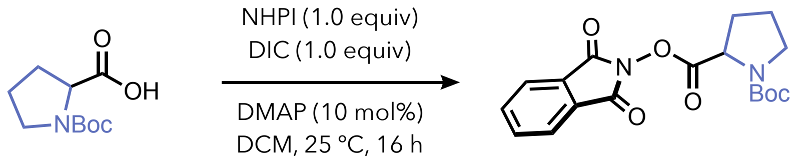

Following General Procedure A, the title product was obtained from *N*-Boc-proline (0.65 g, 3.0 mmol, 1.0 equiv), *N*-hydroxyphthalimide (0.51 g, 3.1 mmol, 1.05 equiv), 4-dimethylaminopyridine (DMAP, 37 mg, 0.30 mmol, 10 mol%) and *N,N'*-diisopropylcarbodiimide (DIC, 0.38 g, 0.47 mL, 3.0 mmol, 1.0 equiv) in DCM (20 mL, 0.15 M). Purification by flash column chromatography in silica gel, using hexanes/EtOAc as solvent (gradient from 95:5 to 9:1). This gave 0.85 g (79%) of the title product as a white foam.

^{1}H NMR (300 MHz, $CDCl_3$) δ 7.99 – 7.76 (m, 4H), 4.79 – 4.59 (m, 1H), 3.70 – 3.40 (m, 2H), 2.51 – 2.33 (m, 2H), 2.14 – 1.95 (m, 2H), 1.52 (s, 9H).

^{13}C NMR (75 MHz, $CDCl_3$) δ 169.6, 161.7, 153.5, 134.8, 128.9, 123.9, 81.1, 57.2, 46.3, 31.4, 28.1, 23.5.

* Some NMR signals are broad and split due to the presence of diastereomeric rotamers.

1,3-Dioxoisoindolin-2-yl cyclohexanecarboxylate (1f)

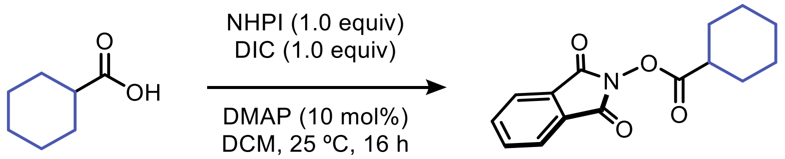

Following General Procedure A, the title product was obtained from cyclohexanecarboxylic acid (0.28 g, 2.2 mmol, 1.0 equiv), *N*-hydroxyphthalimide (0.36 g, 2.2 mmol, 1.0 equiv), 4-dimethylaminopyridine (DMAP, 27 mg, 0.28 mmol, 10 mol%) and *N,N'*-diisopropylcarbodiimide (DIC, 0.28 g, 0.34 mL, 2.2 mmol, 1.0 equiv) in DCM (11 mL, 0.20 M). Purification by flash column chromatography in silica gel, using hexanes/EtOAc as solvent (9:1). R_f (4:1 hexane/EtOAc) = 0.6. This gave 0.42 g (71%) of the title product as a white solid.

^{1}H NMR (300 MHz, $CDCl_3$) δ 7.91 – 7.83 (m, 2H), 7.82 – 7.74 (m, 2H), 2.80 – 2.67 (m, 1H), 2.16 – 2.04 (m, 2H), 1.90 – 1.78 (m, 2H), 1.75 – 1.58 (m, 3H), 1.47 – 1.23 (m, 3H).

^{13}C NMR (75 MHz, $CDCl_3$) δ 172.0, 162.2, 134.8,129.2,124.0, 40.7, 29.0, 25.6, 25.2.

1,3-Dioxoisoindolin-2-yl 6-oxo-6-phenylhexanoate (1g)

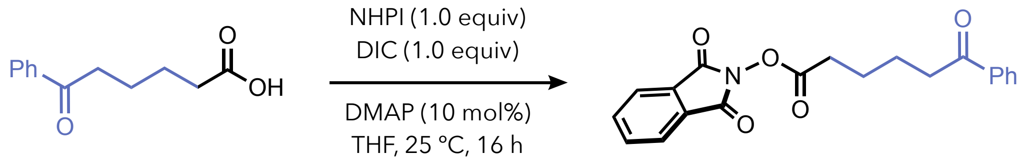

Following General Procedure A, the title product was obtained from 6-oxo-6-phenylhexanoic acid (0.41 g, 2.00 mmol, 1.0 equiv), *N*-hydroxyphthalimide (0.33 g, 2.00 mmol, 1.0 equiv), 4-dimethylaminopyridine (DMAP, 24 mg, 0.20 mmol, 10 mol%) and *N,N'*-diisopropylcarbodiimide (DIC, 0.25 g, 0.31 mL, 2.00 mmol, 1.0 equiv) in THF (10 mL, 0.20 M—*for this compound, THF was used instead of DCM*). Purification by flash column chromatography in silica gel, using hexanes/EtOAc as solvent (7:3). R_f (3:1 hexane/EtOAc) = 0.7. This gave 0.30 g (42%) of the title product as a white solid.

^{1}H NMR (300 MHz, $CDCl_3$) δ 8.06 – 7.93 (m, 2H), 7.95 – 7.83 (m, 2H), 7.85 – 7.73 (m, 2H), 7.63 – 7.50 (m, 1H), 7.52 – 7.41 (m, 2H), 3.14 – 2.98 (m, 2H), 2.85 – 2.66 (m, 2H), 1.98 – 1.86 (m, 4H).

^{13}C NMR (75 MHz, $CDCl_3$) δ 199.6, 169.5, 162.1, 137.0, 134.9, 133.2, 129.1, 128.7, 128.2, 124.1, 38.0, 31.1, 24.4, 23.4.

1,3-Dioxoisoindolin-2-yl 3-(3,4-dimethoxyphenyl) propanoate (1h)

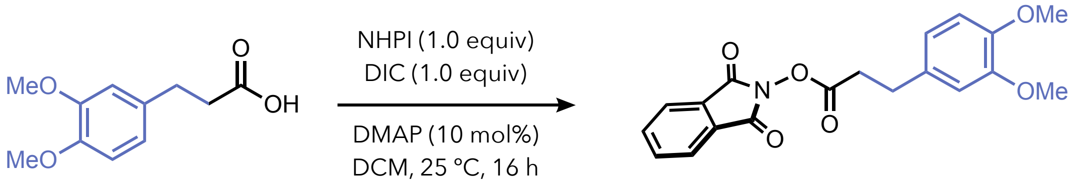

Following General Procedure A, the title product was obtained from 3-(3,4-dimethoxyphenyl)propanoic acid (0.32 g, 1.5 mmol, 1.0 equiv), *N*-hydroxyphthalimide (0.25 g, 1.5 mmol, 1.0 equiv), 4-dimethylaminopyridine (DMAP, 18 mg, 0.15 mmol, 10 mol%) and *N,N'*-diisopropylcarbodiimide (DIC, 0.19 g, 0.23 mL, 1.5 mmol, 1.0 equiv) in DCM (7.5 mL, 0.20 M). Purification by flash column chromatography in silica gel, using hexanes/EtOAc as solvent (gradient from 8:2 to 6:4). R_f (1:1 hexane/EtOAc) = 0.6. This gave 0.53 g (41%) of the title product as a colorless solid.

^{1}H NMR (300 MHz, $CDCl_3$) δ 7.89 (dd, J = 5.6, 3.1 Hz, 2H), 7.79 (dd, J = 5.5, 3.2 Hz, 2H), 6.86 – 6.77 (m, 3H), 3.90 (s, 3H), 3.87 (s, 3H), 3.06 (m, 2H), 2.96 (m, 2H).

^{13}C NMR (75 MHz, $CDCl_3$) δ169.1, 162.1, 149.3, 148.0, 134.9, 132.0, 129.1, 124.1, 120.4, 111.8, 111.7, 56.1, 56.0, 33.2, 30.4.

4. Synthesis of C(sp^2)–H reaction substrates **2**

4.1. General procedure B for the *N*-alkylation of reaction substrates **2**

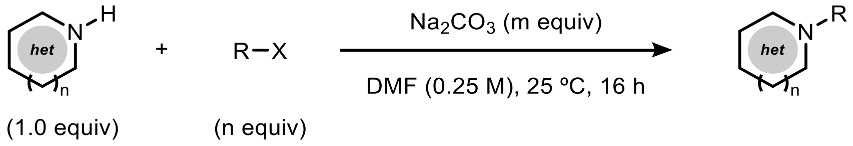

Following typical procedures for the alkylation of heterocyclic compounds,[2] a round-bottom flask of appropriate size (equipped with a Teflon-coated stirring bar) was charged under air atmosphere with the corresponding reaction substrate (1.0 equiv) and sodium carbonate (2.2 equiv). All reagents were dissolved in HPLC-grade DMF (0.25 M), before the alkyl halide (2.2 equiv) was added in a single portion via syringe. The flask was capped with a rubber septum, and the reaction mixture was left stirring at room temperature overnight (ca. 16 h).

After this time, the reaction mixture was transferred to an extraction funnel and diluted with water (ca. 50 mL). The product was extracted with EtOAc (ca. 50 mL), and the organic fraction was washed twice with water and twice with brine, then dried over anhydrous sodium sulfate. After filtration, the crude mixture was concentrated in vacuum. Purification by flash column chromatography in silica gel, using appropriate gradient of hexane and EtOAc afforded the desired product.

→ For the substrates with more than one free N–H group, the amount of alkyl halide and sodium carbonate was adjusted according to the number of N–H sites intended for alkylation. When selective mono-alkylation was desired, 0.9 equiv of alkyl halide and 0.5 equiv of sodium carbonate were employed.

Reaction acceptors **2** were prepared from commercially available heterocyclic compounds, following General Procedure B. This procedure is based on well-established synthetic methods and, while the used compounds are already known in the literature,[2] for the sake of practicality, exact experimental details and NMR data are reported in the following pages.

4.2. Detailed reaction conditions and characterization data for the substrates **2**

2,4-Dibenzyl-1,2,4-triazine-3,5(2*H*,4*H*)-dione (2a)

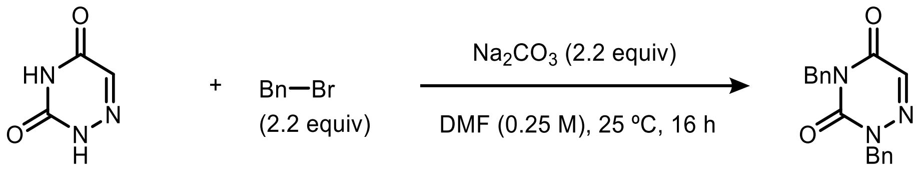

Following General Procedure B, the title product was obtained from benzyl bromide (4.99 g, 3.47 mL, 29.2 mmol, 2.2 equiv), 6-azauracil (1.50 g, 13.3 mmol, 1.0 equiv) and sodium carbonate (3.09 g, 29.2 mmol, 2.2 equiv) in DMF (53 mL, 0.25 M). Purification by flash chromatography in silica gel, using hexanes/EtOAc as solvent (gradient from 95:5 to 75:25). R_f (8:2 hexane/EtOAc) = 0.4. This gave 3.05 g (78%) of the title product as a pale-yellow solid. Characterization data matched the ones reported in the literature.[2]

^{1}H NMR (300 MHz, $CDCl_3$) δ 7.50 – 7.45 (m, 2H), 7.42 – 7.28 (m, 9H), 5.11 (s, 2H), 5.08 (s, 2H).

^{13}C NMR (75 MHz, $CDCl_3$) δ 156.0, 148.8, 135.6, 134.6, 129.5, 128.9, 128.7, 128.5, 128.3, 55.6, 44.1.

2,4-Dimethyl-1,2,4-triazine-3,5(2*H*,4*H*)-dione (2b)

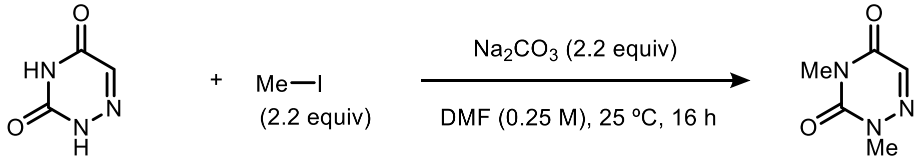

Following General Procedure B, the title product was obtained from methyl iodide (1.38 g, 0.61 mL 9.73 mmol, 2.2 equiv), 6-azauracil (0.50 g, 4.42 mmol, 1.0 equiv) and sodium carbonate (1.03 g, 9.73 mmol, 2.2 equiv) in DMF (18 mL, 0.25 M). Purification by flash column chromatography in silica gel, using hexanes/EtOAc as solvent (gradient from 8:2 to 1:1). R_f (7:3 hexane/EtOAc) = 0.4. This gave 0.22 g (35%) of the title product as a white solid. Characterization data matched the ones reported in the literature.[2]

^{1}H NMR (300 MHz, $CDCl_3$) δ 7.35 (s, 1H), 3.62 (s, 3H), 3.32 (s, 3H).

^{13}C NMR (75 MHz, $CDCl_3$) δ 156.5, 149.1, 133.8, 39.7, 27.0.

2-Benzyl-1,2,4-triazine-3,5(2*H*,4*H*)-dione (2c)

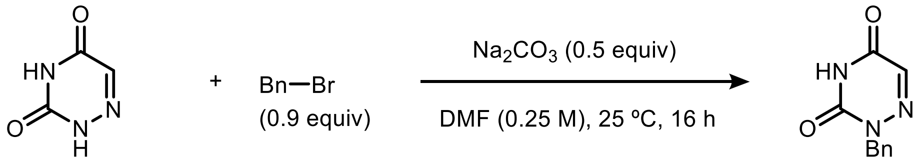

Following General Procedure B, the title product was obtained from benzyl bromide (1.02 g, 0.71 mL 5.97 mmol, 0.90 equiv), 6-azauracil (0.75 g, 6.63 mmol, 1.0 equiv) and sodium carbonate (0.46 g, 3.32 mmol, 0.50 equiv) in DMF (27 mL, 0.25 M). Purification by flash column chromatography in silica gel, using hexanes/EtOAc as solvent (gradient from 9:1 to 6:4). R_f (6:4 hexane/EtOAc) = 0.4. This gave 0.50 g (41%) of the title product as a white solid. Characterization data matched the ones reported in the literature.[2]

^{1}H NMR (300 MHz, $CDCl_3$) δ 10.26 (s, 1H), 7.62 – 7.02 (m, 6H), 5.07 (s, 2H).

^{13}C NMR (75 MHz, $CDCl_3$) δ 156.0, 149.8, 135.7, 135.2, 129.5, 128.8, 128.4, 43.5.

Ethyl 2-(2-benzyl-3,5-dioxo-2,5-dihydro-1,2,4-triazin-4(3*H*)-yl) acetate (2d)

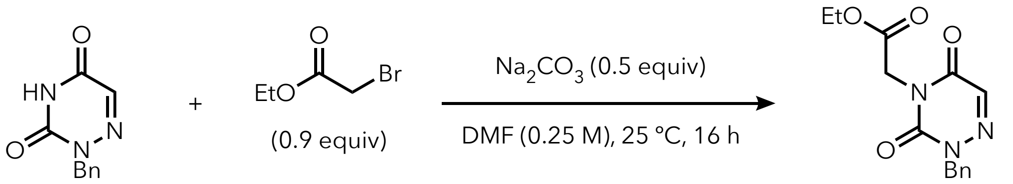

Following General Procedure B, the title product was obtained from ethyl 2-bromoacetate (0.15 g, 98 µL, 0.89 mmol, 0.9 equiv), 4-benzyl-1,2,4-triazine-3,5(2*H*,4*H*)-dione (0.20 g, 0.98 mmol, 1.0 equiv) and sodium carbonate (50 mg, 0.49 mmol, 0.50 equiv) in DMF (4.0 mL, 0.25 M). Purification by flash column chromatography in silica gel, using hexanes/EtOAc as solvent (gradient from 8:2 to 7:3). R_f (6:4 hexane/EtOAc) = 0.4. This gave 200 mg (80%) of the title product as a pale-yellow solid. Characterization data matched the ones reported in the literature.[2]

^{1}H NMR (300 MHz, $CDCl_3$) δ 7.48 – 7.42 (m, 3H), 7.35 – 7.27 (m, 3H), 5.09 (s, 2H), 4.68 (s, 2H), 4.30 – 4.18 (m, 2H), 1.27 (t, J = 7.2 Hz, 3H).

^{13}C NMR (75 MHz, $CDCl_3$) δ 167.4, 155.9, 149.0, 135.3, 135.1, 129.3, 128.8, 128.3, 62.1, 52.9, 44.1, 14.2.

2-Benzyl-4-(prop-2-yn-1-yl)-1,2,4-triazine-3,5(2*H*,4*H*)-dione (2e)

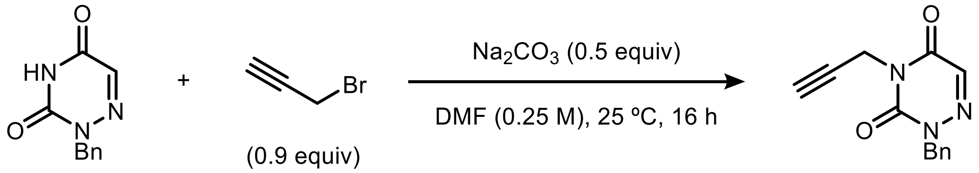

Following General Procedure B, the title product was obtained from 3-bromoprop-1-yne (70 mg, 43 μL, 0.58 mmol, 0.90 equiv), 4-benzyl-1,2,4-triazine-3,5(2*H*,4*H*)-dione (0.13 g, 0.64 mmol, 1.0 equiv) and sodium carbonate (30 mg, 0.32 mmol, 0.50 equiv) in DMF (2.6 mL, 0.25 M). Purification by flash column chromatography in silica gel, using hexanes/EtOAc as solvent (gradient from 9:1 to 75:25). R_f (8:2 hexane/EtOAc) = 0.4. This gave 100 mg (72%) of the title product as a colorless viscous oil. Characterization data matched the ones reported in the literature.[2]

^{1}H NMR (300 MHz, $CDCl_3$) δ 7.51 – 7.44 (m, 3H), 7.37 – 7.28 (m, 3H), 5.09 (s, 2H), 4.73 (d, J = 2.5 Hz, 2H), 2.38 (t, J = 2.5 Hz, 1H).

^{13}C NMR (75 MHz, $CDCl_3$) δ 155.9, 148.3, 135.3, 135.2, 129.7, 128.8, 128.4, 76.7, 73.8, 44.2, 41.6.

1-Benzylquinoxalin-2(1*H*)-one (2f)

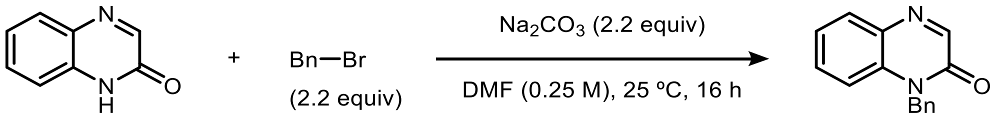

Following General Procedure B, the title product was obtained from benzyl bromide (1.29 g, 0.90 mL, 7.5 mmol, 2.2 equiv), quinoxalin-2-(1*H*)-one (0.50 g, 3.4 mmol, 1.0 equiv) and sodium carbonate (0.80 g, 7.5 mmol, 2.2 equiv) in DMF (14 mL, 0.25 M). Purification by flash column chromatography in silica gel, using hexanes/EtOAc as solvent (gradient from 6:4 to 100% EtOAc). R_f (7:3 hexane/EtOAc) = 0.4. This gave 0.51 g (63%) of the title product as a pale-orange solid. Characterization data matched the ones reported in the literature.[2]

^{1}H NMR (300 MHz, $CDCl_3$) δ 8.44 (s, 1H), 7.92 (dd, J = 7.9, 1.6 Hz, 1H), 7.53 – 7.46 (m, 1H), 7.39 – 7.27 (m, 7H), 5.52 (s, 2H).

^{13}C NMR (75 MHz, $CDCl_3$) δ 155.3, 150.4, 135.1, 133.8, 132.7, 131.1, 130.8, 129.1, 127.9, 127.0, 123.9, 114.8, 45.7.

5. Redox-neutral C–H alkylation with redox-active esters

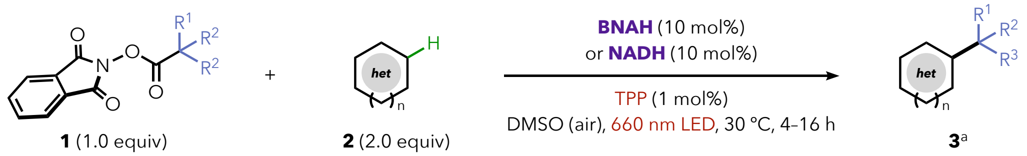

5.1. General procedure C for red-light-promoted alkylation reactions at small scale

Reaction at 0.025 mmol scale (for NMR yield): A 10 mL screw-cap glass culture tube equipped with a Teflon-coated stirring bar was charged, under air atmosphere, with the corresponding redox-active ester **1** (0.025 mmol, 1.0 equiv), BNAH (10 mol%) or NADH (10 mol%), the corresponding heterocyclic acceptor **2** (2.0 equiv) and tetraphenylporphyrin (TPP, 5 mol%). This was followed by the addition of DMSO (1.0 mL, 25 mM for **1**). The reaction tube was loaded in the sample holder of the photoreactor (see Section 2.2 for photochemical set up details). After ca. 1 min of pre-stirring, the reaction tube was irradiated with 2 red Kessil LEDs (660 nm) while stirring and cooling down with the reactor fan system (temperature was determined to be ca. 30 ºC).

After the specified reaction time (usually 4 h or 16 h), the reaction tube was extracted from the reactor, and 1.0 equiv of 1,3,5-trimethoxybenzene was added as an internal standard in 1.0 mL of EtOAc (alternatively, this internal standard can be added before the reaction; we found 1,3,5-trimethoxybenzene not to interfere with the reported chemistry). The reaction mixture was further diluted with ca. 2.0 mL of EtOAc and 2.0 mL of water and shaken energetically. The organic fraction was dried over anhydrous sodium or magnesium sulfate and centrifuged. The organic fraction was dried in vacuum, and the resulting crude product was analyzed by ^{1}H NMR.

5.2. General procedure D for the scale-up and isolation of photochemical reactions

After determining the reaction yield by ^{1}H NMR using 1,3,5-trimethoxybenzene as an internal standard, one of the two systems (using substoichiometric amounts of BNAH or NADH; generally, the former, for simple economic/practical reasons) was used for scale-up (8x, 0.20 mmol), purification, and characterization of each of the redox-neutral alkylation products.

Unless stated otherwise, scale-up reactions for product isolation were carried out at a 0.20 mmol scale at a concentration of 50 mM (4.0 mL of DMSO), which ensures efficient irradiation of the entire reaction mixture within the window directly exposed to the Kessil lamps in our experimental setup.

Thus, an example of a scaled-up reaction would be carried out as follows:

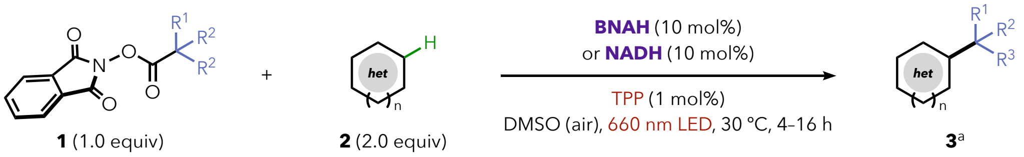

Reaction at 0.20 mmol scale (for isolation): A 10 mL screw-cap glass culture tube equipped with a Teflon-coated magnetic stirring bar was charged, under air atmosphere, with the corresponding redox-active ester **1** (0.20 mmol, 1.0 equiv), BNAH **4** (0.020 mmol, 10 mol%), the corresponding heterocyclic acceptor **2** (0.40 mmol, 2.0 equiv) and the photocatalyst (TPP, 1–5 mol%). Everything was dissolved in DMSO (4 mL, 50 mM), and the reaction tube was loaded in the sample holder of the photoreactor (see Section 2.2 for details on the set up). After ca. 1 min of pre-stirring, the reaction tube was irradiated with two Kessil LEDs (660 nm) while stirring and cooling down with the reactor fan system. After the specified reaction time (usually 4 h or 16 h), the mixture was transferred to an extraction funnel and diluted with water (ca. 50 mL) The product was extracted with EtOAc (ca. 50 mL), and the organic layer was washed twice with water and twice with brine, before drying with anhydrous sodium sulfate. After filtration, the crude mixture was concentrated in vacuum. Purification using flash column chromatography in silica gel with the corresponding gradient of hexane/EtOAc afforded the desired product. Further experimental details and characterization data of the isolated products are reported below.

5.3. Detailed reaction conditions and characterization data for products **3**

2,4-Dibenzyl-6-(*tert*-butyl)-1,2,4-triazine-3,5(2*H*,4*H*)-dione (3a)

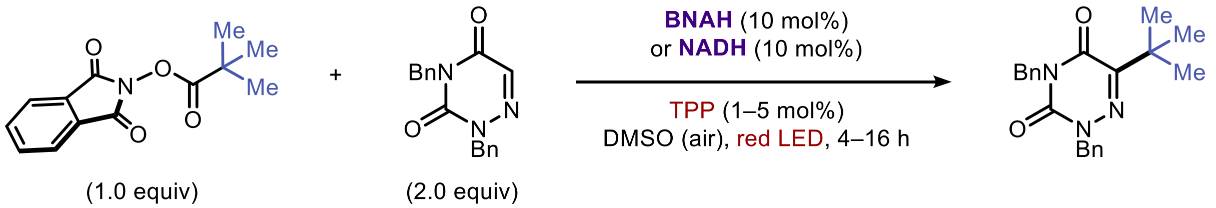

Substoichiometric reductant comparison: As determined by NMR using 1,3,5-trimethoxybenzene as internal standard at 0.025 mmol scale, the title product was obtained (using General Procedure C) in 88% yield using BNAH (10 mol%), or in 82% yield using NADH (10 mol%).

Isolated product (***Note****: this reaction was ran at 1 mmol to evaluate scalability*): Following General Procedure D, the title product was obtained from 1,3-dioxoisoindolin-2-yl pivalate (0.25 g, 1.0 mmol, 1.0 equiv, 1-benzyl-1,4-dihydronicotinamide), BNAH (21 mg, 0.10 mmol, 10 mol%) and 2,4-dibenzyl-1,2,4-triazine-3,5(2*H*,4*H*)-dione (0.59 g, 2.0 mmol, 2.0 equiv), with tetraphenylporphyrin as photocatalyst (TPP, 6.1 mg, 0.01 mmol, 1.0 mol%) in DMSO (20 mL, 50 mM) and red-light irradiation (16 h), after purification by flash column chromatography in silica gel, using hexanes/EtOAc as solvent (gradient from 9:1 to 7:3). This gave 299 mg (86% isolated yield) of the title product as a pale-brown solid (color due to trace—less than 1%, not detected by ^{1}H NMR—porphyrin contamination, which has an identical R_f than the product).

$\boldsymbol{R_f}$ (8:2 hexanes/EtOAc): 0.4

^{1}H NMR (300 MHz, $CDCl_3$) δ 7.50 – 7.45 (m, 2H), 7.45 – 7.39 (m, 2H), 7.39 – 7.26 (m, 6H), 5.08 (s, 4H), 1.33 (s, 9H).

^{13}C NMR (75 MHz, $CDCl_3$) δ 154.9, 150.5, 149.2, 136.1, 136.0, 129.5, 129.1, 128.8, 128.7, 128.3, 128.0, 55.4, 44.1, 37.4, 27.9.

HRMS (ESI): calculated for $C_{21}H_{23}N_3O_2$ $[M+H]^+$: 350.1857; found: 350.1863.

Furthermore, in this 1 mmol-scale experiment, other reaction components were also isolated, recovering phthalimide byproduct (pale-yellow solid, 103 mg, 70% yield) and unreacted excess 2,4-dibenzyl-1,2,4-triazine-3,5(2*H*,4*H*)-dione (pale-yellow solid, 321 mg, 90% recovery).

The scaled-up reaction was performed in a 50 mL plastic Falcon tube instead of a glass culture tube:

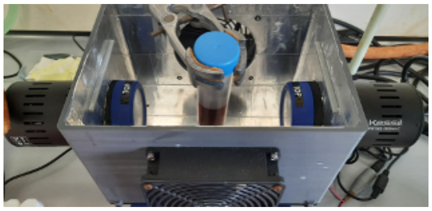

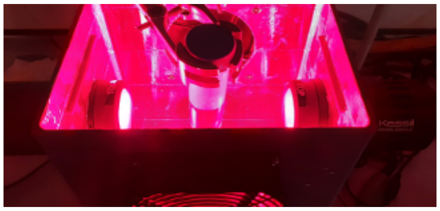

6-(Adamantan-1-yl)-2,4-dibenzyl-1,2,4-triazine-3,5(2*H*,4*H*)-dione (3b)

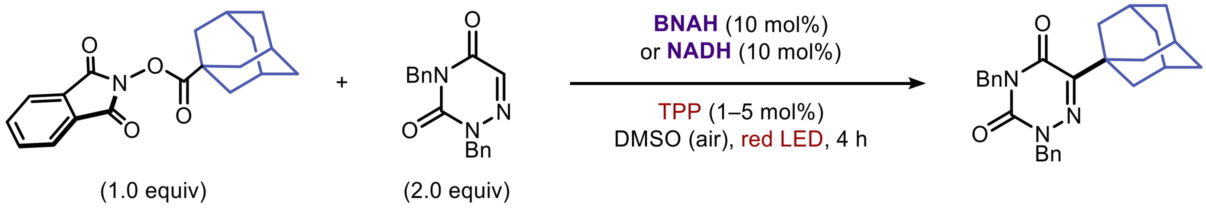

Substoichiometric reductant comparison: As determined by NMR using 1,3,5-trimethoxybenzene as internal standard at 0.025 mmol scale, the title product was obtained (using General Procedure C) in 89% yield using BNAH (10 mol%), or in 90% yield using NADH (10 mol%).

Isolated product: Following General Procedure D, the title product was obtained from 1,3-dioxoisoindolin-2-yl adamantane-1-carboxylate (65 mg, 0.20 mmol, 1.0 equiv), 1-benzyl-1,4-dihydronicotinamide (BNAH, 4.3 mg, 0.020 mmol, 10 mol%) and 2,4-dibenzyl-1,2,4-triazine-3,5(2*H*,4*H*)-dione (117 mg, 0.40 mmol, 2.0 equiv), with tetraphenylporphyrin as photocatalyst (TPP, 1.2 mg, 0.0020 mmol, 1 mol%) in DMSO (4.0 mL, 50 mM) and red-light irradiation (4 h), after purification by flash column chromatography in silica gel, using hexanes/EtOAc as solvent (gradient from 99:1 to 95:5). This gave 64 mg (75% isolated yield) of the title product as a white solid.

R_f (9:1 hexanes/EtOAc): 0.4

^{1}H NMR (300 MHz, $CDCl_3$) δ 7.50 – 7.46 (m, 2H), 7.45 – 7.41 (m, 2H), 7.40 – 7.28 (m, 6H), 5.07 (d, J = 5.1 Hz, 4H), 2.09 – 2.00 (m, 9H), 1.79 – 1.73 (m, 6H).

^{13}C NMR (75 MHz, $CDCl_3$) δ 154.8, 150.1, 149.0, 136.1, 136.0, 129.5, 129.1, 128.8, 128.7, 128.3, 128.0, 55.4, 44.0, 39.1, 36.9, 28.4.

HRMS (ESI): calculated for $C_{27}H_{29}N_3O_2$ $[M+H]^+$: 428.2333; found: 428.2332.

2,4-Dibenzyl-6-(5-(2,5-dimethylphenoxy)-2-methylpentan-2-yl)-1,2,4-triazine-3,5(2*H*,4*H*)-dione (3c)

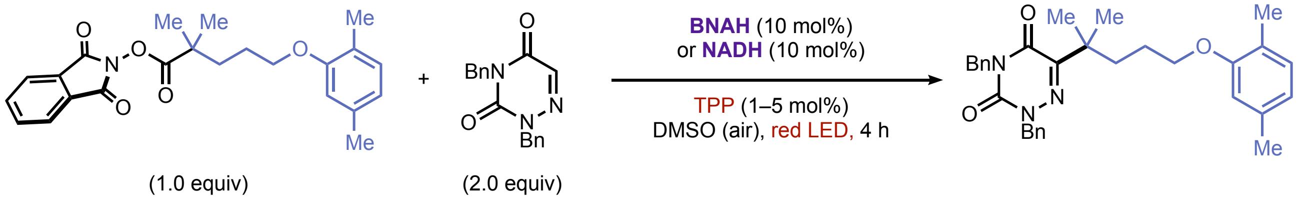

Substoichiometric reductant comparison: As determined by NMR using 1,3,5-trimethoxybenzene as internal standard at 0.025 mmol scale, the title product was obtained (using General Procedure C) in 52% yield using BNAH (10 mol%), or in 64% yield using NADH (10 mol%).

Isolated product: Following General Procedure D, the title product was obtained from 1,3-dioxoisoindolin-2-yl 5-(2,5-dimethylphenoxy)-2,2-dimethylpentanoate (79 mg, 0.20 mmol, 1.0 equiv), 1-benzyl-1,4-dihydronicotinamide (BNAH, 4.3 mg, 0.020 mmol, 10 mol%) and 2,4-dibenzyl-1,2,4-triazine-3,5(2*H*,4*H*)-dione (117 mg, 0.40 mmol, 2.0 equiv), with tetraphenylporphyrin as photocatalyst (TPP, 1.2 mg, 0.0020 mmol, 1 mol%) in DMSO (4.0 mL, 50 mM) and red-light irradiation (4 h), after purification by flash column chromatography in silica gel, using hexanes/EtOAc as solvent (gradient from 99:1 to 96:4). This gave 68 mg (68% isolated yield) of the title product as a pale-yellow oil.

$\boldsymbol{R_f}$ (9:1 hexanes/EtOAc): 0.4

^{1}H NMR (300 MHz, $CDCl_3$) δ 7.49 – 7.39 (m, 4H), 7.39 – 7.26 (m, 6H), 7.01 (d, J = 7.4 Hz, 1H), 6.67 (d, J = 7.5 Hz, 1H), 6.57 (s, 1H), 5.09 (s, 2H), 5.06 (s, 2H), 3.85 (t, J = 6.5 Hz, 2H), 2.31 (s, 3H), 2.18 (s, 3H), 2.02 – 1.93 (m, 2H), 1.65 – 1.53 (m, 2H), 1.35 (s, 6H).

^{13}C NMR (75 MHz, $CDCl_3$) δ 157.1, 154.9, 149.4, 149.0, 136.6, 136.0, 135.9, 130.4, 129.3, 129.0, 128.8, 128.7, 128.3, 128.0, 123.7, 120.8, 112.0, 68.0, 55.4, 36.0, 26.1, 25.2, 21.6, 15.9.

HRMS (ESI): calculated for $C_{31}H_{35}N_3O_3$ $[M+H]^+$: 498.2751; found: 498.2750.

2,4-Dibenzyl-6-(1-methylcyclohexyl)-1,2,4-triazine-3,5(2*H*,4*H*)-dione (3d)

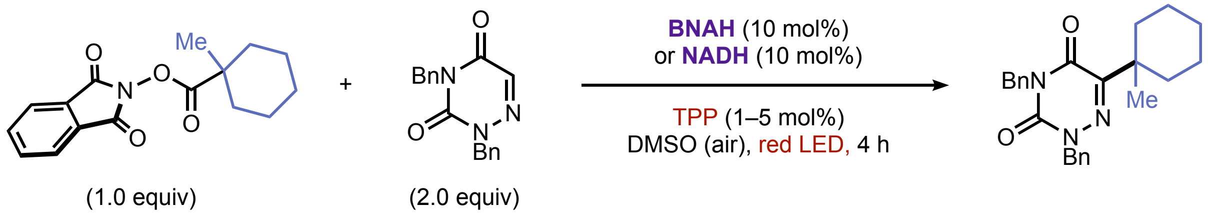

Substoichiometric reductant comparison: As determined by NMR using 1,3,5-trimethoxybenzene as internal standard at 0.025 mmol scale, the title product was obtained (using General Procedure C) in 67% yield using BNAH (10 mol%), or in 65% yield using NADH (10 mol%).

Isolated product: Following General Procedure D, the title product was obtained from 1,3-dioxoisoindolin-2-yl 1-methylcyclohexane-1-carboxylate (58 mg, 0.20 mmol, 1.0 equiv), 1-benzyl-1,4-dihydronicotinamide (BNAH, 4.3 mg, 0.020 mmol, 10 mol%) and 2,4-dibenzyl-1,2,4-triazine-3,5(2*H*,4*H*)-dione (117 mg, 0.40 mmol, 2.0 equiv), with tetraphenylporphyrin as photocatalyst (TPP, 1.2 mg, 0.0020 mmol, 1 mol%) in DMSO (4.0 mL, 50 mM) and red-light irradiation (4 h), after purification by flash column chromatography in silica gel, using hexanes/EtOAc as solvent (gradient from 99:1 to 97:3). This gave 52 mg (67% isolated yield) of the title product as a pale-purple solid (color due to trace porphyrin contamination, same Rf than product; not detected by ^{1}H NMR).$\boldsymbol{R_f}$ (8:2 hexanes/EtOAc): 0.6

^{1}H NMR (300 MHz, $CDCl_3$) δ 7.57 – 7.18 (m, 10H), 5.10 (d, J = 4.6 Hz, 4H), 2.24 – 2.12 (m, 2H), 1.64 – 1.39 (m, 8H), 1.31 (s, 3H).

^{13}C NMR (75 MHz, $CDCl_3$) δ 154.9, 149.6, 148.9, 136.0, 135.9, 129.3, 128.9, 128.7, 128.6, 128.2, 127.9, 55.3, 44.0, 40.7, 35.3, 26.3, 24.6, 22.4.

HRMS (ESI): calculated for $C_{24}H_{27}N_3O_2$ $[M+H]^+$: 390.2176; found: 390.2179.

tert-Butyl -2-(2,4-dibenzyl-3,5-dioxo-2,3,4,5-tetrahydro-1,2,4-triazin-6-yl) pyrrolidine-1-carboxylate (3e)

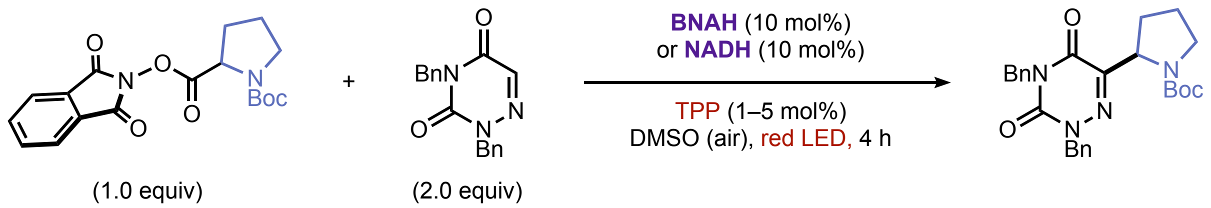

Substoichiometric reductant comparison: As determined by NMR using 1,3,5-trimethoxybenzene as internal standard at 0.025 mmol scale, the title product was obtained (using General Procedure C) in 61% yield using BNAH (10 mol%), or in 86% yield using NADH (10 mol%).

Isolated product: Following General Procedure D, the title product was obtained from 1-(*tert*-butyl) 2-(1,3-dioxoisoindolin-2-yl)-pyrrolidine-1,2-dicarboxylate (72 mg, 0.20 mmol, 1.0 equiv), 1-benzyl-1,4-dihydronicotinamide (BNAH, 4.3 mg, 0.020 mmol, 10 mol%) and 2,4-dibenzyl-1,2,4-triazine-3,5(2*H*,4*H*)-dione (117 mg, 0.40 mmol, 2.0 equiv), with tetraphenylporphyrin as photocatalyst (TPP, 1.2 mg, 0.0020 mmol, 1 mol%) in DMSO (4.0 mL, 50 mM) and red-light irradiation (4 h), after purification by flash column chromatography in silica gel, using hexanes/EtOAc as solvent (gradient from 98:2 to 75:25). This gave 90 mg (82% yield isolated together with ca. 15 mol% of phthalimide) of the title product as a yellow solid.

R_f (8:2 hexanes/EtOAc): 0.3

^{1}H NMR (300 MHz, $CDCl_3$) δ 7.49 – 7.39 (m, 2H), 7.37 – 7.22 (m, 8H), 5.18 – 4.92 (m, 5H), 3.63 – 3.35 (m, 2H), 2.29 – 2.12 (m, 1H), 1.92 – 1.76 (m, 3H), 1.43 (s, 4.5H), 1.16 (s, 4.5H).

^{13}C NMR (75 MHz, $CDCl_3$) δ 155.1, 154.2, 153.7, 149.0, 146.0, 144.5, 135.7, 134.4, 132.9, 129.6, 129.4, 128.9, 128.8, 128.7, 128.4, 128.2, 79.6, 56.5, 55.9, 55.5, 46.8, 46.6, 44.3, 31.7, 30.5, 28.6, 28.3, 23.6, 23.0.

* **Note:** some NMR signals are broad and split due to the presence of diastereomeric rotamers.

HRMS (ESI): calculated for $C_{26}H_{30}N_4O_4$ $[M+H]^+$: 463.2340; found: 463.2341.

2,4-Dibenzyl-6-cyclohexyl-1,2,4-triazine-3,5(2*H*,4*H*)-dione (3f)

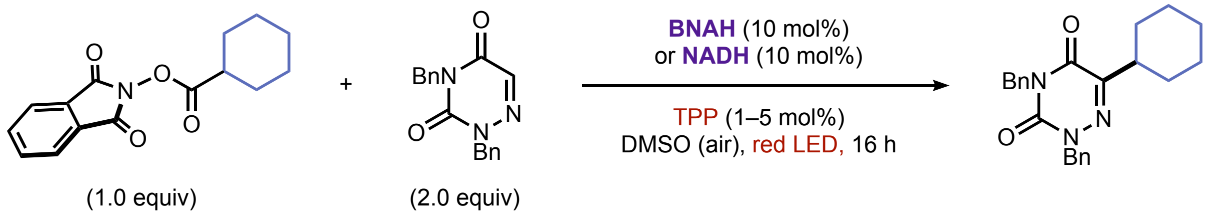

Substoichiometric reductant comparison: As determined by NMR using 1,3,5-trimethoxybenzene as internal standard at 0.025 mmol scale, the title product was obtained (using General Procedure C) in 89% yield using BNAH (10 mol%), or in 93% yield using NADH (10 mol%).

Isolated product: Following General Procedure D, the title product was obtained from 1,3-dioxoisoindolin-2-yl cyclohexanecarboxylate (55 mg, 0.20 mmol, 1.0 equiv), 1-benzyl-1,4-dihydronicotinamide (BNAH, 4.3 mg, 0.020 mmol, 10 mol%) and 2,4-dibenzyl-1,2,4-triazine-3,5(2*H*,4*H*)-dione (117 mg, 0.40 mmol, 2.0 equiv), with tetraphenylporphyrin as photocatalyst (TPP, 1.2 mg, 0.0020 mmol, 1 mol%) in DMSO (4.0 mL, 50 mM) and red-light irradiation (16 h), after purification by flash column chromatography in silica gel, using hexanes/EtOAc as solvent (gradient from 99:1 to 96:4). This gave 65 mg (82% isolated yield) of the title product as a pale purple solid (color due to trace porphyrin contamination, same R_f than product; not detected by ^{1}H NMR).

$\boldsymbol{R_f}$ (9:1 hexanes/EtOAc): 0.4

^{1}H NMR (300 MHz, $CDCl_3$) δ 7.53 – 7.47 (m, 2H), 7.45 – 7.27 (m, 8H), 5.09 (d, J = 2.7 Hz, 4H), 2.98 – 2.75 (m, 1H), 1.96 – 1.63 (m, 6H), 1.48 – 1.30 (m, 4H).

^{13}C NMR (75 MHz, $CDCl_3$) δ 155.8, 149.1, 136.0, 129.6, 128.9, 128.8, 128.6, 128.2, 128.1, 55.4, 44.3, 38.5, 30.6, 26.2, 26.1.

HRMS (ESI): calculated for $C_{23}H_{25}N_3O_2$ $[M+H]^+$: 376.2020; found: 376.2024.

2,4-Dibenzyl-6-(5-oxo-5-phenylpentyl)-1,2,4-triazine-3,5(2*H*,4*H*)-dione (3g)

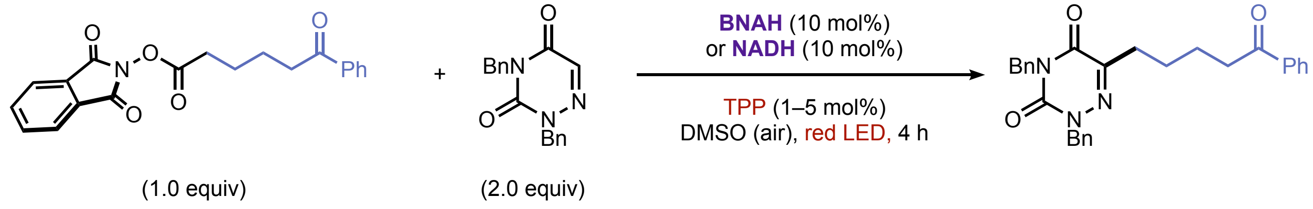

Substoichiometric reductant comparison: As determined by NMR using 1,3,5-trimethoxybenzene as internal standard at 0.025 mmol scale, the title product was obtained (using General Procedure C) in 75% yield using BNAH (10 mol%), or in 91% yield using NADH (10 mol%).

Isolated product: Following General Procedure D, the title product was obtained from 1,3-dioxoisoindolin-2-yl-6-oxo-6-phenylhexanoate (70 mg, 0.20 mmol, 1.0 equiv), 1-benzyl-1,4-dihydronicotinamide (BNAH, 4.3 mg, 0.020 mmol, 10 mol%) and 2,4-dibenzyl-1,2,4-triazine-3,5(2*H*,4*H*)-dione (117.3 mg, 0.40 mmol, 2.0 equiv), with tetraphenylporphyrin as photocatalyst (TPP, 1.2 mg, 0.0020 mmol, 1 mol%) in DMSO (4.0 mL, 50 mM) and red-light irradiation (4 h), after purification by flash column chromatography in silica gel, using hexanes/EtOAc as solvent (gradient from 95:5 to 85:15). This gave 30 mg (33% isolated yield) of the title product as a yellow oil.

R_f (8:2 hexanes/EtOAc): 0.4

^{1}H NMR (300 MHz, $CDCl_3$) δ 7.99 – 7.93 (m, 2H), 7.60 – 7.53 (m, 1H), 7.50 – 7.42 (m, 4H), 7.42 – 7.27 (m, 8H), 5.08 (d, J = 1.6 Hz, 4H), 3.00 (t, J = 6.9 Hz, 2H), 2.67 (t, J = 7.1 Hz, 2H), 1.84 – 1.69 (m, 4H).

^{13}C NMR (75 MHz, $CDCl_3$) δ 200.1, 156.1, 149.1, 145.3, 137.1, 135.9, 135.8, 133.1, 129.5, 128.8, 128.7, 128.7, 128.2, 55.3, 44.3, 38.3, 30.2, 25.8, 23.7.

HRMS (ESI): calculated for $C_{28}H_{27}N_3O_3$ $[M+H]^+$: 454.2125; found: 454.2127.

2,4-Dibenzyl-6-(3,4-dimethoxyphenethyl)-1,2,4-triazine-3,5(2*H*,4*H*)-dione (3h)

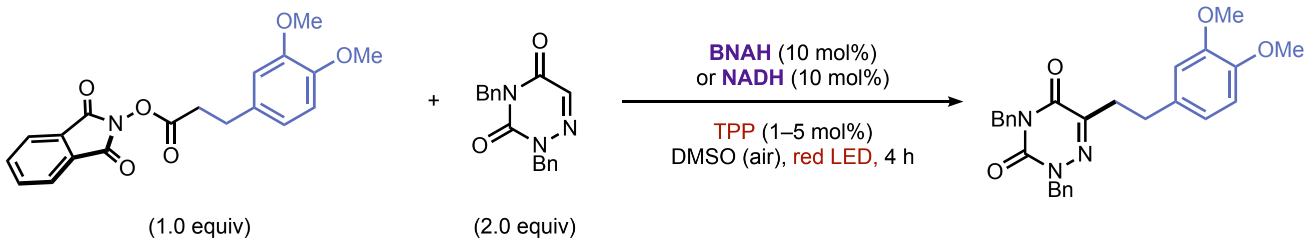

Substoichiometric reductant comparison: As determined by NMR using 1,3,5-trimethoxybenzene as internal standard at 0.025 mmol scale, the title product was obtained (using General Procedure C) in 63% yield using BNAH (10 mol%), or in 36% yield using NADH (10 mol%).

Isolated product: Following General Procedure D, the title product was obtained from 1,3-dioxoisoindolin-2-yl 3-(3,4-dimethoxyphenyl)propanoate (71 mg, 0.20 mmol, 1.0 equiv), 1-benzyl-1,4-dihydronicotinamide (BNAH, 4.3 mg, 0.020 mmol, 10 mol%) and 2,4-dibenzyl-1,2,4-triazine-3,5(2*H*,4*H*)-dione (117 mg, 0.40 mmol, 2.0 equiv), with tetraphenylporphyrin as photocatalyst (TPP, 1.2 mg, 0.0020 mmol, 1 mol%) in DMSO (4.0 mL, 50 mM) and red-light irradiation (4 h), after purification by flash column chromatography in silica gel, using hexanes/EtOAc as solvent (gradient from 95:5 to 85:15). This gave 50 mg (55% isolated yield) of the title product as a pale-yellow oil.

R_f (8:2 hexanes/EtOAc): 0.6

^{1}H NMR (300 MHz, $CDCl_3$) δ 7.51 – 7.42 (m, 2H), 7.30 (d, *J* = 6.3 Hz, 8H), 6.78 – 6.67 (m, 3H), 5.07 (d, *J* = 5.0 Hz, 4H), 3.85 (s, 3H), 3.80 (s, 3H), 2.92 (s, 4H).

^{13}C NMR (75 MHz, $CDCl_3$) δ 156.0, 148.9, 148.9, 147.4, 144.7, 135.8, 135.6, 133.2, 129.4, 128.7, 128.6, 128.5, 128.2, 128.1, 120.4, 111.6, 111.2, 55.9, 55.8, 55.3, 44.2, 32.2, 31.8.

HRMS (ESI): calculated for $C_{27}H_{27}N_3O_4$ $[M+H]^+$: 458.2074; found: 458.2074.

6-(*tert*-Butyl)-2,4-dimethyl-1,2,4-triazine-3,5(2*H*,4*H*)-dione (3i)

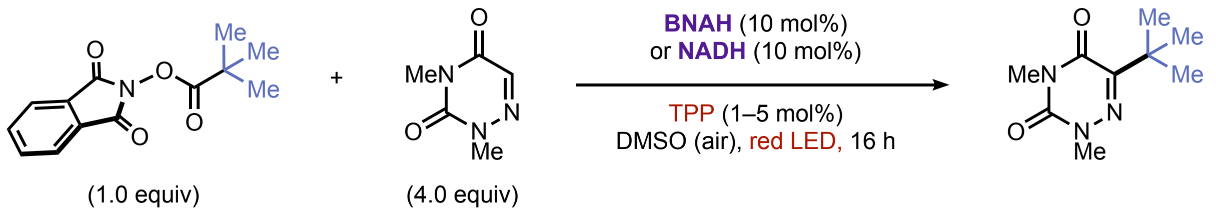

Substoichiometric reductant comparison: As determined by NMR using 1,3,5-trimethoxybenzene as internal standard at 0.025 mmol scale, the title product was obtained (using General Procedure C) in 67% yield using BNAH (10 mol%), or in 58% yield using NADH (10 mol%).

Isolated product: Following General Procedure D, the title product was obtained from 1,3-dioxoisoindolin-2-yl pivalate (50 mg, 0.20 mmol, 1.0 equiv), 1-benzyl-1,4-dihydronicotinamide (BNAH, 4.3 mg, 0.020 mmol, 10 mol%) and 2,4-dimethyl-1,2,4-triazine-3,5(2*H*,4*H*)-dione (115.7 mg, 0.82 mmol, 4.0 equiv), with tetraphenylporphyrin as photocatalyst (TPP, 1.2 mg, 0.0020 mmol, 1 mol%) in DMSO (4.0 mL, 50 mM) and red-light irradiation (16 h), after purification by flash column chromatography in silica gel, using hexanes/EtOAc as solvent (gradient from 95:5 to 85:15). This gave 25 mg (63% isolated yield) of the title product as a yellow oil.

R_f (8:2 hexanes/EtOAc): 0.5

^{1}H NMR (300 MHz, $CDCl_3$) δ 3.60 (s, 3H), 3.32 (s, 3H), 1.32 (s, 9H).

^{13}C NMR (75 MHz, $CDCl_3$) δ 155.5, 149.8, 149.6, 39.5, 37.1, 27.8, 27.1.

HRMS (ESI): calculated for $C_9H_{15}N_3O_2$ $[M+H]^+$: 198.1237; found: 198.1241.

2-Benzyl-6-(tert-butyl)-1,2,4-triazine-3,5(2*H*,4*H*)-dione (3j)

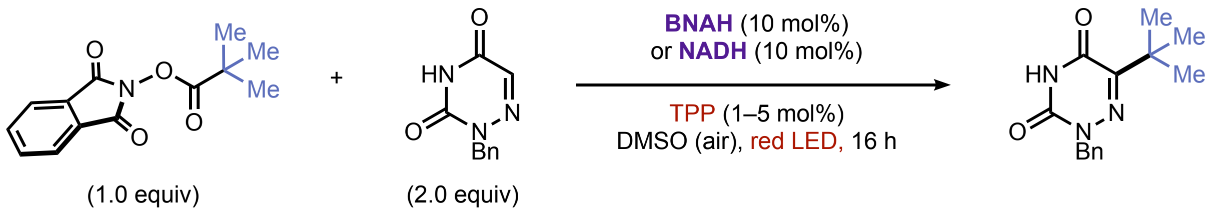

Substoichiometric reductant comparison: As determined by NMR using 1,3,5-trimethoxybenzene as internal standard at 0.025 mmol scale, the title product was obtained (using General Procedure C) in 75% yield using BNAH (10 mol%), or in 53% yield using NADH (10 mol%).

Isolated product: Following General Procedure D, the title product was obtained from 1,3-dioxoisoindolin-2-yl pivalate (50 mg, 0.20 mmol, 1.0 equiv), 1-benzyl-1,4-dihydronicotinamide (BNAH, 4.3 mg, 0.020 mmol, 10 mol%) and 2-benzyl-1,2,4-triazine-3,5(2*H*,4*H*)-dione (81 mg, 0.40 mmol, 2.0 equiv), with tetraphenylporphyrin as photocatalyst (TPP, 1.2 mg, 0.0020 mmol, 1 mol%) in DMSO (4.0 mL, 50 mM) and red-light irradiation (16 h), after purification by flash column chromatography in silica gel, using hexanes/EtOAc as solvent (gradient from 9:1 to 85:15). This gave 41 mg (79% isolated yield) of the title product as a pale-yellow solid.

R_f (8:2 hexanes/EtOAc): 0.5

^{1}H NMR (300 MHz, $CDCl_3$) δ 10.15 (s, 1H), 7.50 – 7.45 (m, 2H), 7.35 – 7.25 (m, 3H), 5.09 (s, 2H), 1.33 (s, 9H).

^{13}C NMR (75 MHz, $CDCl_3$) δ 154.9, 151.5, 150.4, 135.8, 129.4, 128.7, 128.1, 43.5, 37.3, 27.8.

HRMS (ESI): calculated for $C_{14}H_{17}N_3O_2$ $[M+H]^+$: 260.1394; found: 260.1394.

Ethyl 2-(2-benzyl-6-(tert-butyl)-3,5-dioxo-2,5-dihydro-1,2,4-triazin-4(3*H*)-yl) acetate (3k)

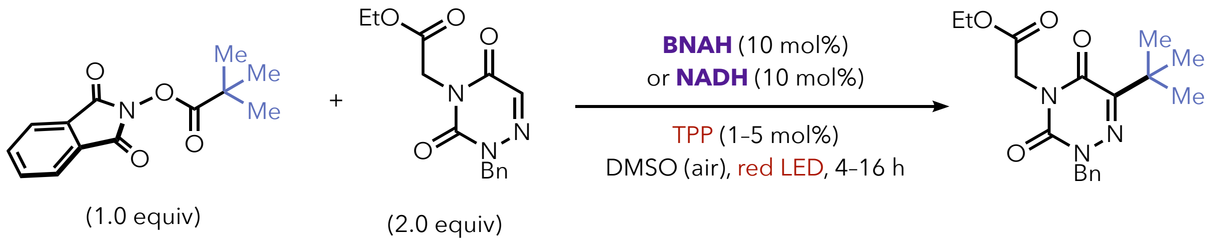

Substoichiometric reductant comparison: As determined by NMR using 1,3,5-trimethoxybenzene as internal standard at 0.025 mmol scale, the title product was obtained (using General Procedure C) in 90% yield using BNAH (10 mol%), or in 60% yield using NADH (10 mol%).

Isolated product: Following General Procedure D, the title product was obtained from 1,3-dioxoisoindolin-2-yl pivalate (50 mg, 0.20 mmol, 1.0 equiv), 1-benzyl-1,4-dihydronicotinamide (BNAH, 4.3 mg, 0.020 mmol, 10 mol%) and ethyl 2-(2-benzyl-3,5-dioxo-2,5-dihydro-1,2,4-triazin-4(3*H*)-yl)acetate (115.7 mg, 0.40 mmol, 2.0 equiv), with tetraphenylporphyrin as photocatalyst (TPP, 1.2 mg, 0.0020 mmol, 1 mol%) in DMSO (4.0 mL, 50 mM) and red-light irradiation (16 h), after purification by flash column chromatography in silica gel, using hexanes/EtOAc as solvent (gradient from 95:5 to 85:15). This gave 56 mg (81% isolated yield) of the title product as a yellow oil.

R_f (8:2 hexanes/EtOAc): 0.5

^{1}H NMR (300 MHz, $CDCl_3$) δ 7.47 – 7.42 (m, 2H), 7.35 – 7.27 (m, 3H), 5.10 (s, 2H), 4.65 (s, 2H), 4.24 (q, J = 7.1 Hz, 2H), 1.32 (s, 9H), 1.27 (t, J = 7.1 Hz, 3H).

^{13}C NMR (75 MHz, $CDCl_3$) δ 167.7, 154.9, 151.0, 149.4, 135.9, 129.2, 128.7, 128.0, 61.9, 52.9, 44.1, 37.3, 27.8, 14.2.

HRMS (ESI): calculated for $C_{18}H_{23}N_3O_4$ $[M+H]^+$: 346.1761; found: 346.1761.

2-Benzyl-6-(tert-butyl)-4-(prop-2-yn-1-yl)-1,2,4-triazine-3,5(2*H*,4*H*)-dione (3l)

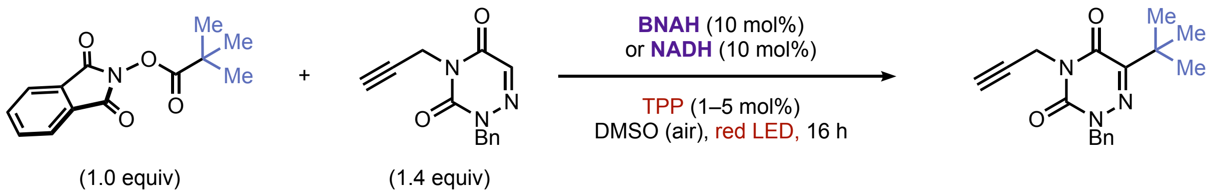

Substoichiometric reductant comparison: As determined by NMR using 1,3,5-trimethoxybenzene as internal standard at 0.025 mmol scale, the title product was obtained (using General Procedure C) in 72% yield using BNAH (10 mol%), or in 51% yield using NADH (10 mol%).

Isolated product: Following General Procedure D, the title product was obtained from 1,3-dioxoisoindolin-2-yl pivalate (50 mg, 0.20 mmol, 1.0 equiv), 1-benzyl-1,4-dihydronicotinamide (BNAH, 4.3 mg, 0.020 mmol, 10 mol%) and 2-benzyl-4-(prop-2-yn-1-yl)-1,2,4-triazine-3,5(2*H*,4*H*)-dione (67.0 mg, 0.28 mmol, 1.4 equiv), with tetraphenylporphyrin as photocatalyst (TPP, 1.2 mg, 0.0020 mmol, 1 mol%) in DMSO (4.0 mL, 50 mM) and red-light irradiation (16 h), after purification by flash column chromatography in silica gel, using hexanes/EtOAc as solvent (gradient from 99:1 to 9:1). This gave 50 mg (84% isolated yield) of the title product as a pale-beige oil.

R_f (8:2 hexanes/EtOAc): 0.4

^{1}H NMR (300 MHz, $CDCl_3$) δ 7.52 – 7.46 (m, 2H), 7.36 – 7.27 (m, 3H), 5.09 (s, 2H), 4.70 (d, J = 2.5 Hz, 2H), 2.34 (t, J = 2.5 Hz, 1H), 1.33 (s, 9H).

^{13}C NMR (75 MHz, $CDCl_3$) δ 154.9, 151.1, 148.7, 135.9, 129.6, 128.7, 128.1, 77.3, 73.3, 44.1, 41.6, 37.3, 27.8.

HRMS (ESI): calculated for $C_{17}H_{19}N_3O_2$ $[M+H]^+$: 298.1550; found: 298.1552.

3-(*tert*-Butyl)-2-phenyl-2*H*-indazole (3n)

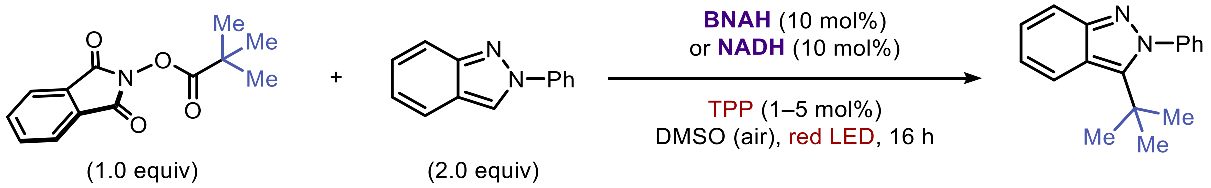

Substoichiometric reductant comparison: As determined by NMR using 1,3,5-trimethoxybenzene as internal standard at 0.025 mmol scale, the title product was obtained (using General Procedure C) in 45% yield using BNAH (10 mol%), or in 39% yield using NADH (10 mol%).

Isolated product: Following General Procedure D, the title product was obtained from 1,3-dioxoisoindolin-2-yl pivalate (50 mg, 0.20 mmol, 1.0 equiv), 1-benzyl-1,4-dihydronicotinamide (BNAH, 4.3 mg, 0.020 mmol, 10 mol%) and 2-phenyl-2*H*-indazole (77,7 mg, 0.40 mmol, 2.0 equiv), with tetraphenylporphyrin as photocatalyst (TPP, 1.2 mg, 0.0020 mmol, 1 mol%) in DMSO (4.0 mL, 50 mM) and red-light irradiation (16 h), after purification by flash column chromatography in silica gel, using hexanes/EtOAc as solvent (gradient from 95:5 to 8:2). This gave 26 mg (52% isolated yield) of the title product as a pale-brown solid.

$\boldsymbol{R_f}$ (9:1 hexanes/EtOAc): 0.3

^{1}H NMR (300 MHz, $CDCl_3$) δ 7.95 (d, J = 8.8 Hz, 1H), 7.68 (d, J = 8.7 Hz, 1H), 7.52 – 7.42 (m, 5H), 7.32 – 7.27 (m, 1H), 7.08 – 7.02 (m, 1H), 1.43 (s, 9H).

^{13}C NMR (75 MHz, $CDCl_3$) δ 148.5, 144.6, 143.0, 129.5, 128.6, 128.2, 126.2, 122.7, 120.9, 119.8, 117.9, 34.9, 32.0.

HRMS (ESI): calculated for $C_{17}H_{18}N_2$ $[M+H]^+$: 251.1543; found: 251.1544.

2-(*tert*-Butyl) quinoxaline (3o)

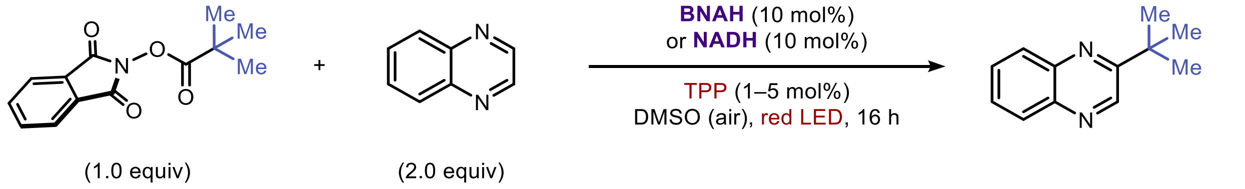

Substoichiometric reductant comparison: As determined by NMR using 1,3,5-trimethoxybenzene as internal standard at 0.025 mmol scale, the title product was obtained (using General Procedure C) in 84% yield using BNAH (10 mol%), or in 74% yield using NADH (10 mol%).

Isolated product: Following General Procedure D, the title product was obtained from 1,3-dioxoisoindolin-2-yl pivalate (50 mg, 0.20 mmol, 1.0 equiv), 1-benzyl-1,4-dihydronicotinamide (BNAH, 4.3 mg, 0.020 mmol, 10 mol%) and quinoxaline (52 mg, 46 µL, 0.40 mmol, 2.0 equiv), with tetraphenylporphyrin as photocatalyst (TPP, 1.2 mg, 0.0020 mmol, 1 mol%) in DMSO (4.0 mL, 50 mM) and red-light irradiation (16 h), after purification by preparative TLC, using hexanes/EtOAc as solvent (95:5, two elutions). This gave 30 mg (81% isolated yield) of the title product as a colorless solid.

R_f (9:1 hexanes/EtOAc): 0.5

^{1}H NMR (300 MHz, $CDCl_3$) δ 8.98 (s, 1H), 8.07 – 8.04 (m, 2H), 7.74 – 7.67 (m, 2H), 1.51 (s, 9H).

^{13}C NMR (75 MHz, $CDCl_3$) δ 163.8, 143.6, 141.8, 140.9, 129.8, 129.4, 129.0, 129.0, 37.4, 29.9.

HRMS (ESI): calculated for $C_{12}H_{14}N_2$ $[M+H]^+$: 187.1230; found: 187.1231.

1-Benzyl-3-(*tert*-butyl) quinoxalin-2(1*H*)-one (3p)

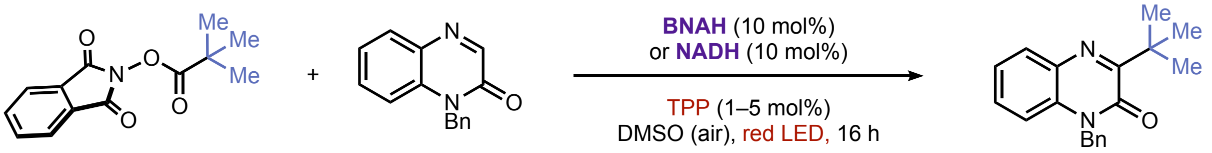

Substoichiometric reductant comparison: As determined by NMR using 1,3,5-trimethoxybenzene as internal standard at 0.025 mmol scale, the title product was obtained (using General Procedure C) in 53% yield using BNAH (10 mol%), or in 22% yield using NADH (10 mol%).

Isolated product: Following General Procedure D, the title product was obtained from 1,3-dioxoisoindolin-2-yl pivalate (50 mg, 0.20 mmol, 1.0 equiv), 1-benzyl-1,4-dihydronicotinamide (BNAH, 4.3 mg, 0.020 mmol, 10 mol%) and 1-benzylquinoxalin-2(1*H*)-one (95 mg, 0.40 mmol, 2.0 equiv), with tetraphenylporphyrin as photocatalyst (TPP, 1.2 mg, 0.0020 mmol, 1 mol%) in DMSO (4.0 mL, 50 mM) and red-light irradiation (16 h), after purification by flash column chromatography in silica gel, using hexanes/EtOAc as solvent (gradient from 95:5 to 8:2). This gave 35 mg (60% isolated yield) of the title product as a purple solid (color due to trace porphyrin contamination, same Rf than product; not detected by ^{1}H NMR).

R_f (9:1 hexanes/EtOAc): 0.5

^{1}H NMR (300 MHz, $CDCl_3$) δ 7.89 – 7.83 (m, 1H), 7.43 – 7.17 (m, 8H), 5.50 (s, 2H), 1.55 (d, J = 0.8 Hz, 9H).

^{13}C NMR (75 MHz, $CDCl_3$) δ 165.6, 153.8, 135.7, 132.8, 132.5, 130.3, 129.6, 129.0, 127.7, 126.9, 123.4, 114.2, 45.6, 39.7, 28.2.

HRMS (ESI): calculated for $C_{19}H_{20}N_2O$ $[M+H]^+$: 293.1648; found: 293.1650.

3-(*tert*-Butyl)-4*H*-chromen-4-one (3q)

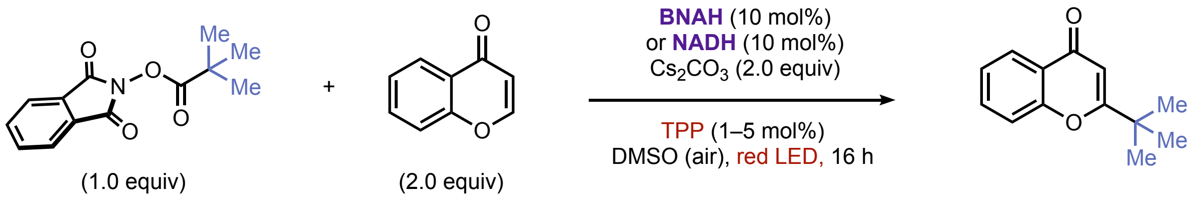

Substoichiometric reductant comparison: As determined by NMR using 1,3,5-trimethoxybenzene as internal standard at 0.025 mmol scale, the title product was obtained (using General Procedure C, but adding 2.0 equiv of Cs_2CO_3) in 55% yield using BNAH (10 mol%), or in 70% yield using NADH (10 mol%).

Isolated product: Following General Procedure D, the title product was obtained from 1,3-dioxoisoindolin-2-yl pivalate (50 mg, 0.20 mmol, 1.0 equiv), nicotinamide adenine dinucleotide (NADH, 28 mg, 0.040 mmol, 20 mol%—*for this particular compound, the scale-up/isolation reaction was carried out with NADH due to better yield and selectivity*), cesium carbonate (130 mg, 0.40 mmol, 2.0 equiv) and 4*H*-chromen-4-one (59 mg, 0.40 mmol, 2.0 equiv), with tetraphenylporphyrin as photocatalyst (TPP, 6.1 mg, 0.010 mmol, 5 mol%) in DMSO (4.0 mL, 50 mM) and red-light irradiation (16 h). After purification by flash column chromatography in silica gel, using hexanes/EtOAc as solvent (gradient from 95:5 to 8:2), the product was further purified by preparative TLC (hexanes/EtOAc 9:1, two elutions). This gave 28 mg (69% isolated yield) of the title product as a pale-brown solid.

R_f (8:2 hexanes/EtOAc): 0.5

^{1}H NMR (300 MHz, $CDCl_3$) δ 8.18 (dd, J = 8.0, 1.7 Hz, 1H), 7.68 – 7.60 (m, 1H), 7.47 – 7.34 (m, 2H), 6.28 (s, 1H), 1.36 (s, 9H).

^{13}C NMR (75 MHz, $CDCl_3$) δ 179.1, 176.2, 156.7, 133.6, 125.7, 125.0, 123.6, 118.0, 106.9, 36.7, 28.0.

HRMS (ESI): calculated for $C_{13}H_{14}O_2$ $[M+H]^+$: 203.1067; found: 203.1069.

5.4. Unsuccessful substrates and scope limitations

In order to explore the limitations of the reactions, besides the main scope, we also tested the following substrates under our standard reaction conditions. In all cases, we managed to observe some amount of product, but not enough for isolation/characterization without further re-optimizing the conditions.

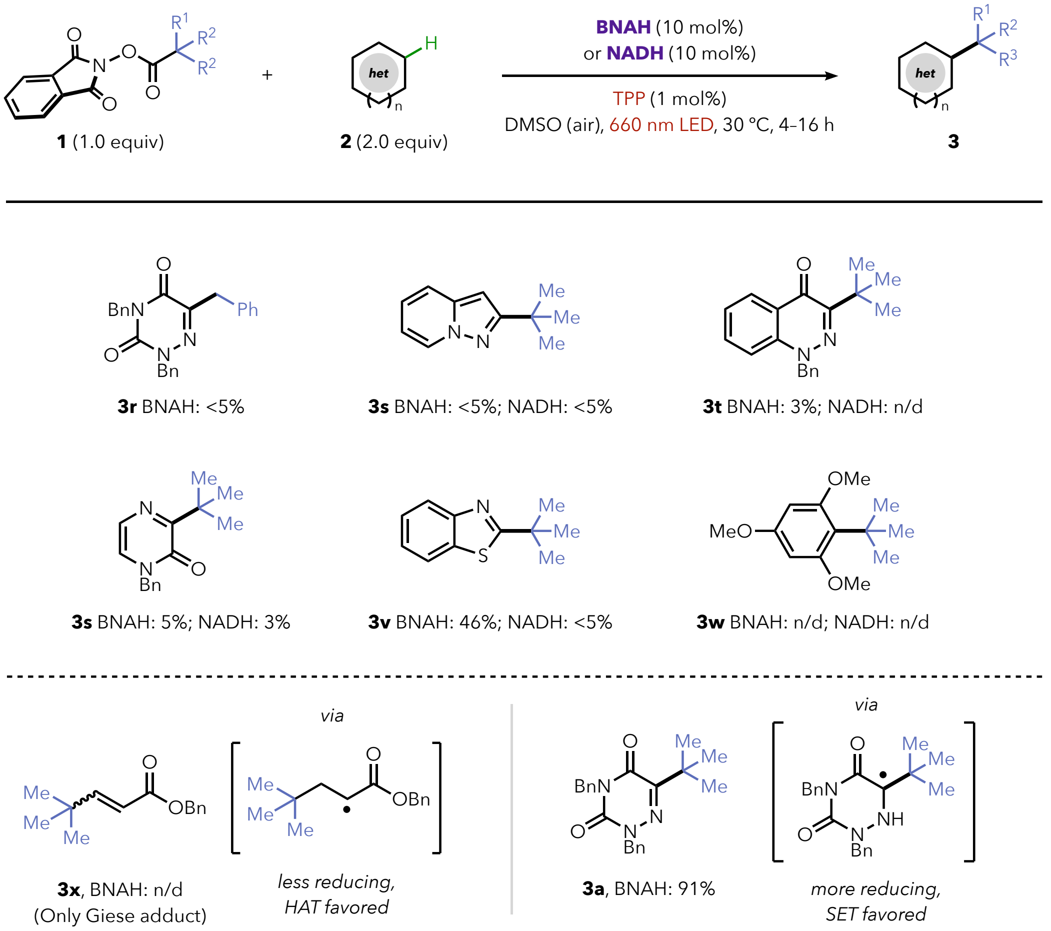

Figure S5. Additional substrates with substoichiometric reductant. Yields determined by ^{1}H NMR using 1,3,5-trimethoxybenzene as internal standard. Standard conditions: **1** (0.025 mmol, 25 mM), **2** (2.0 equiv), BNAH or NADH (10 mol%) and TPP (5 mol%) in DMSO under 660 nm light irradiation for 16 h. n/d = not detected.

Out of all RAEs (**1**) evaluated, only the one derived from phenylacetic acid was not successful to deliver product **3r**. While some other electron-poor heteroaromatics are reactive (see Figure 3 in the main text), some shown here showed much lower reactivity (**3t**, **3s** and **3v**). Product **3v** was obtained in 46% NMR yield using BNAH, but since purification was unsuccessful, and NADH only led to small amounts of product, we did not explore this reaction further. Electron-rich aromatics did not engage in the transformation (**3w**). Interestingly, while chromone is a successful acceptor partner, regular Michael acceptors such as benzyl acrylate (**3x**) are not active. This highlights the importance of the formation of highly reducing radical intermediates after the initial radical addition for the reaction to proceed, consistent with our mechanistic picture (see Figure 5 of the main text).

6. Reaction development, optimization and control experiments

General conditions

Unless stated otherwise, all reaction development was carried out using the standard protocols described in General Procedures C, under the correspondingly modified reaction conditions as described in this section. Unless stated otherwise, for product **3a**, in all optimization, development or control reactions, yields were determined by GCMS adding 1.0 equiv of 1,3,5-trimethoxybenzene as internal standard, according to the following calibration curve (**3a** = PDT in the table), where yield = (area of the product/area of the internal standard) divided by 1.47.

mmol PDT	mmol IS	area PDT/ area IS	mmol PDT/ mmol IS
0,00083	0,00500	0,174	0,167
0,00167	0,00500	0,387	0,333
0,00250	0,00500	0,654	0,500
0,00333	0,00500	0,875	0,667
0,00417	0,00500	1,159	0,833

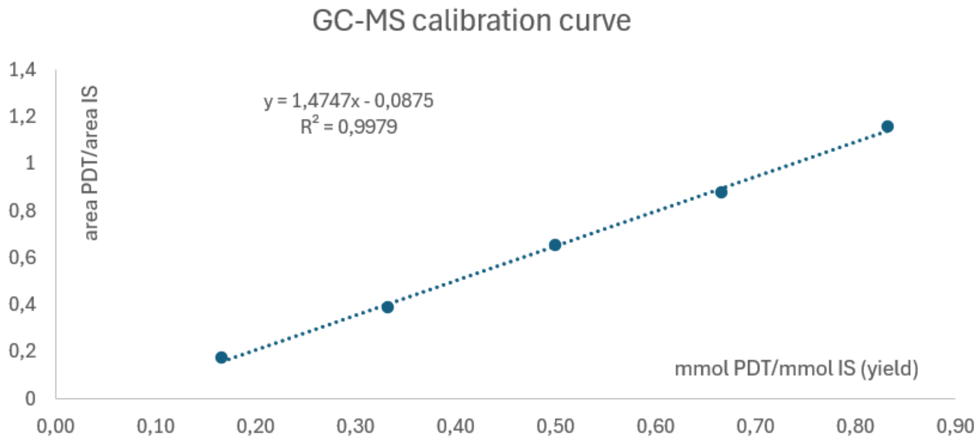

Figure S6. Calibration data for the determination of the yield of **3a** (PDT) by GCMS. Concentration of 1,3,5-trimethoxybenzene (IS) = 1 mg/mL

The ratio between **3a** and **4a** was initially determined by ^{1}H NMR. Since we found the **3a**/**4a** GC–MS integral ratio to be a good approximation of the actual ratio of products, for practical reasons, GC–MS was used to determine the ratio between both products.

Initial reaction development with red light

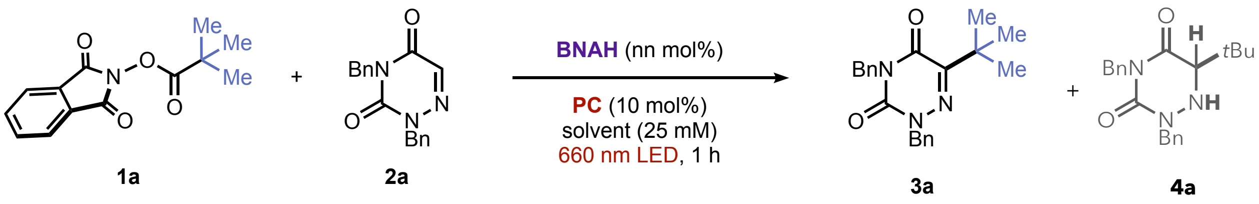

Entry	Conditions / Deviations	Yield **3a** (**3a**/**4a**)
1	TPP (10 mol%), red light, **without BNAH**	<1%
2	TPP (10 mol%), red light, BNAH (150 mol%)	64% (3:1)
3	TPP (10 mol%), red light, BNAH (10 mol%)	91% (>20:1)
4	TPP (10 mol%), red light, **without BNAH**, 16 h instead of 1 h	12%
5	TPP (10 mol%), **no light**, BNAH (10 mol%)	<1%
6	no photocatalyst, red light, BNAH (10 mol%)	<1%
7	TPP (5 mol%), red light, NADH (10 mol%) instead of BNAH	82% (>20:1)
8	TPP (5 mol%), red light, NADPH (20 mol%) instead of BNAH	68% (>20:1)
9	TPP (5 mol%), red light, DIPEA (20 mol%) instead of BNAH	38% (6:1)
10	TPP (5 mol%), red light, Et_3N (20 mol%) instead of BNAH	27% (5:1)

Unless stated otherwise, yields determined by ^1H NMR using 1,3,5-trimethoxybenzene as IS. n/d = not detected

Table S1. Initial development and control experiments of the red-light system.

Initial reaction development with green light

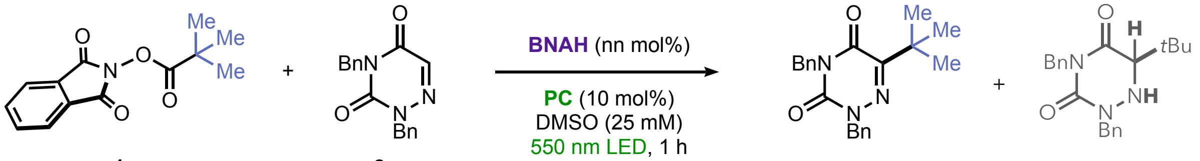

1a 2a 3a 4a

Entry	Conditions / Deviations	Yield **3a** (**3a**/**4a**)
1	Eosin Y (10 mol%), green light, **without BNAH**	18% (>20:1)
2	Eosin Y (10 mol%), green light, BNAH (150 mol%)	45% (2:1)
3	Eosin Y (10 mol%), green light, BNAH (10 mol%)	80% (>20:1)
4	Eosin Y (10 mol%), green light, **without BNAH**, 4 h instead of 1 h	22% (>20:1)
5	Eosin Y (10 mol%), **no light**, BNAH (10 mol%)	<1%
6	no photocatalyst, green light, BNAH (10 mol%)	<1%

Unless stated otherwise, yields determined by ^{1}H NMR using 1,3,5-trimethoxybenzene as IS. n/d = not detected

Table S2. Initial development and control experiments of the green-light system.

Initial screening with blue light

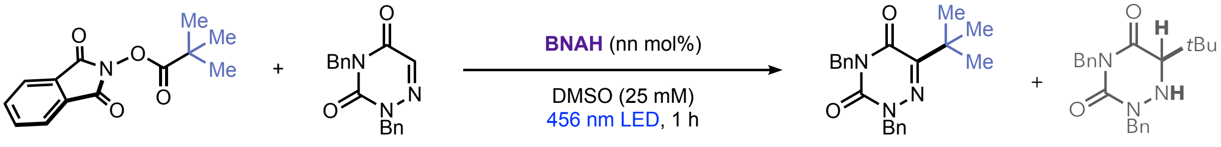

1a 2a 3a 4a

Entry	Conditions / Deviations	Yield **3a** (**3a**/**4a**)
1	blue light, without BNAH	<1%
2	blue light, BNAH (150 mol%)	75% (9:1)
3	blue light, BNAH (20 mol%)	21% (>20:1)

Unless stated otherwise, yields determined by ^{1}H NMR using 1,3,5-trimethoxybenzene as IS. n/d = not detected

Table S3. Initial screening of the direct blue-light excitation system.

Further analysis of this direct-excitation system was carried out and is detailed in Section 9.2.

Study of other red-light photocatalysts in different solvent mixtures

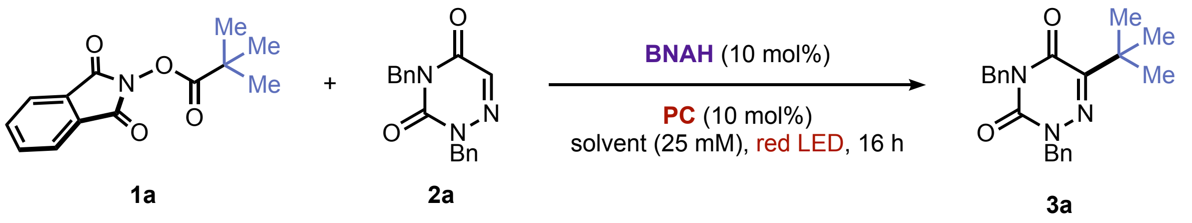

Entry	Conditions / Deviations	**3a** Yield
1	red light, TPP (10 mol%), DMSO	88%
2	red light, TPP (10 mol%), DMSO/water (9:1)	18%
3	red light, ZnTPP (10 mol%), DMSO	18%
4	red light, ZnTPP (10 mol%), DMSO/water (9:1)	20%
5	red light, methylene blue (10 mol%), DMSO	traces
6	red light, methylene blue (10 mol%), DMSO/water (9:1)	n/d
7	red light, TCPP (10 mol%), DMSO	71%

Unless stated otherwise, yields determined by ^1H NMR using 1,3,5-trimethoxybenzene as IS. n/d = not detected

Table S4. Screening of the red-light photocatalysts. TCPP = tetrakis(4-carboxyphenyl)porphyrin.

Evaluation of other solvents

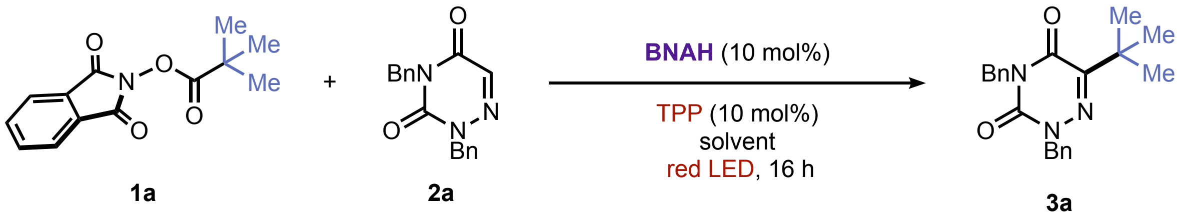

Entry	Conditions / Deviations	**3a** Yield
1	DMSO as solvent (25 mM)	88%
2	MeCN as solvent (25 mM)	n/d
3	DMF as solvent (25 mM)	2%
4	DMA as solvent (25 mM)	8%
5	DCM as solvent (25 mM)	n/d

Unless stated otherwise, yields determined by ^1H NMR using 1,3,5-trimethoxybenzene as IS. n/d = not detected

Table S5. Screening of different solvents.

Effect of concentration and catalyst loading

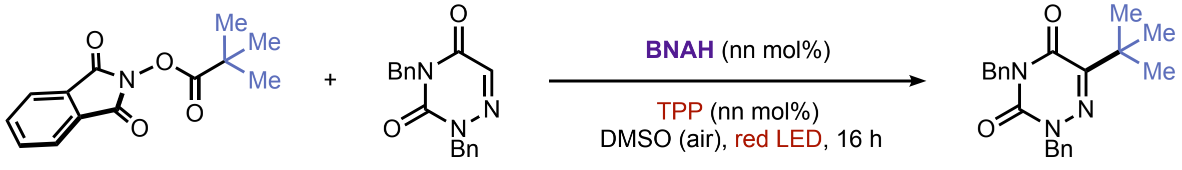

1a **2a** **3a**

Entry	Conditions / Deviations	**3a** Yield
1	**25 mM**, TPP (1 mol%), BNAH (10 mol%)	97%
2	**50 mM**, TPP (1 mol%), BNAH (10 mol%)	90%
3	**100 mM**, TPP (1 mol%), BNAH (10 mol%)	81%
4	25 mM, TPP (5 mol%), BNAH (5 mol %)	47%
5	25 mM, TPP (5 mol%), BNAH (20 mol %)	56%
6	25 mM, TPP (15 mol%), BNAH (5 mol %)	41%
7	25 mM, TPP (15 mol%), BNAH (20 mol %)	60%

Unless stated otherwise, yields determined by ^{1}H NMR using 1,3,5-trimethoxybenzene as IS. n/d = not detected

Table S6. Screening of different concentrations and catalyst loadings.

Screening and selectivity switch using chromone as substrate

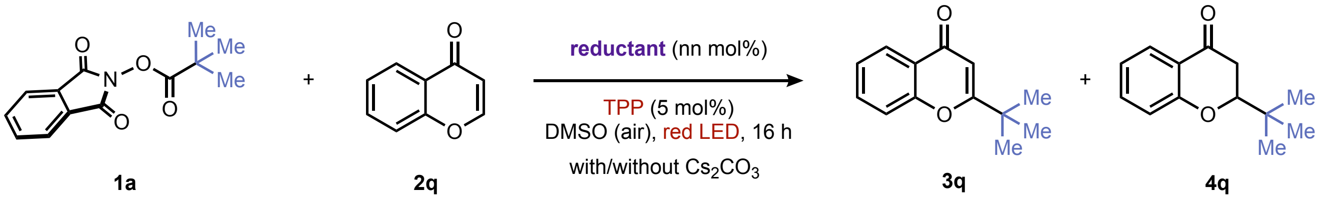

1a 2q 3q 4q

Entry	Conditions / Deviations	Recov. **1a**	**3p** Yield	**4b** Yield
1	BNAH (5 mol%)	77%	5%	n/d
2	BNAH (10 mol%)	29%	21%	13%
3	BNAH (20 mol%)	n/d	28%	18%
4	BNAH (50 mol%)	3%	18%	20%
5	BNAH (20 mol%), Cs_2CO_3	n/d	47%	8%
6	BNAH (150 mol%), Cs_2CO_3	n/d	5%	21%
7	NADH (10 mol%)	51%	15%	7%
8	NADH (20 mol%), Cs_2CO_3	n/d	68%	2%
9	NADH (150 mol%), Cs_2CO_3	n/d	15%	28%

Unless stated otherwise, yields determined by ^{1}H NMR using 1,3,5-trimethoxybenzene as IS. n/d = not detected

Table S7. Screening of conditions for the C–H alkylation of chromone.

7. Biocompatible reactions using bioavailable components

7.1. Reactions using bioreductant-containing media

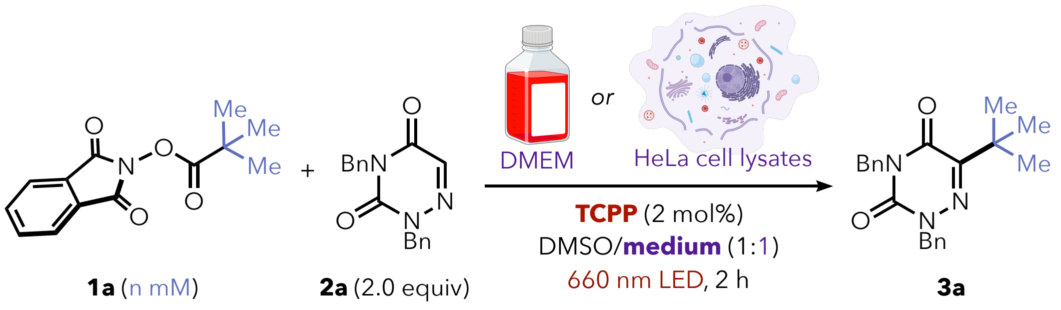

Unless stated otherwise, a 10 mL screw-cap glass culture tube equipped with a Teflon-coated stirring bar was charged, under air atmosphere, with redox-active ester **1a** (0.020 mmol, 1.0 equiv), BNAH or NADH (10 mol%), azaurazil derivative **2b** (2.0 equiv), tetrakis(4-carboxyphenyl) porphyrin (TCPP, 2 mol%) and 1.0 equiv of 1,3,5-trimethoxybenzene as internal standard. This was followed by the addition of DMSO (1.0 mL) and the corresponding aqueous medium (1.0 mL). The reaction tube was loaded in the sample holder of the photoreactor (see Section 2.2 for photochemical set up details). After ca. 1 min of pre-stirring, the reaction tube was irradiated with 2 red Kessil LEDs (660 nm) while stirring and cooling down with the reactor fan system. After 2 h of irradiation, the reaction tube was extracted from the photoreactor and the mixture was diluted with ca. 3 mL of EtOAc and ca. 3 mL of water and shaken energetically. The organic fraction was dried over anhydrous sodium or magnesium sulfate and centrifuged, then it was diluted in HPLC-grade DCM (ca. 1 mg/mL of **3a**) and filtered through an HPLC filter, before analyzing by GCMS to determine the yield of **3a**.

Different aqueous media were tested: (i) distilled water, (ii) DMEM (Gibco Dulbecco's Modified Eagle Medium, [+] 4.5 g/L D-glucose, L-glutamine, [+] 100 mg/L sodium pyruvate; REF: 11995-065) or (iii) mammalian HeLa cell lysates in PBS buffer (concentration of cell residue of 3 mg/mL). The obtained results are reported below. In all cases, yields are and average of two replicates ran on different days:

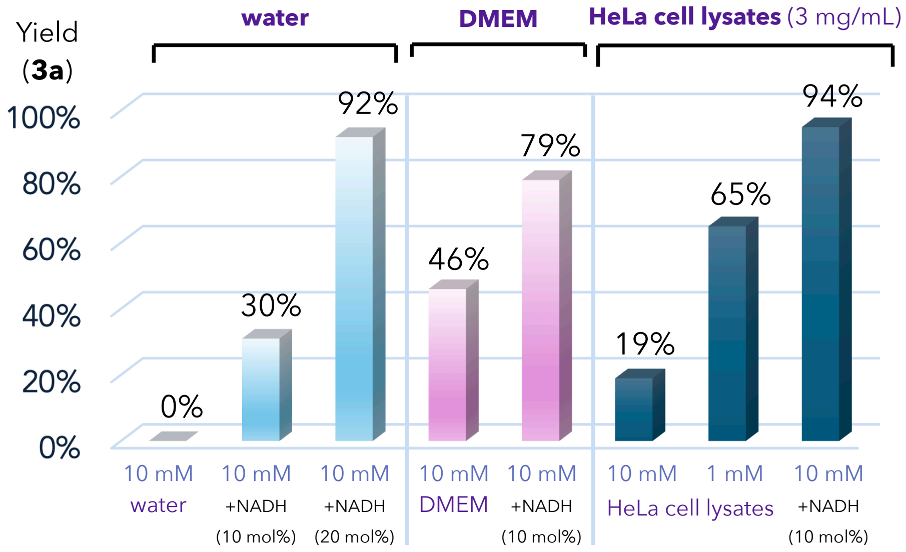

7.2. Reactions using natural-pigment extracts from spinach leaves

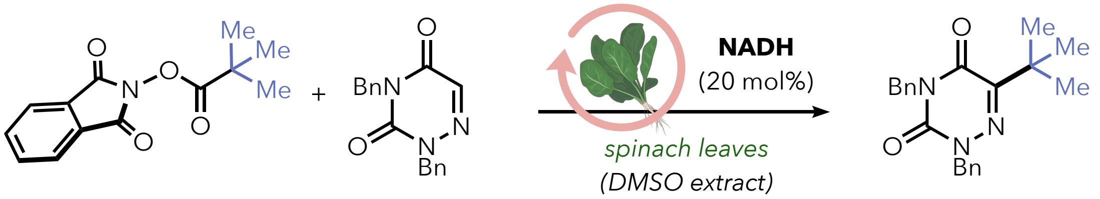

1a (10 mM) **2a** (2.0 equiv) 660 nm LED, 3 h **3a**

DMSO spinach extract was obtained as follows: 10 g of fresh spinach (Florette brand) were cut down to pieces of roughly 2x2 mm, and charged into a 50 mL Falcon tube equipped with a Teflon-coated magnetic stirring bar. This was followed by the addition of 10 mL of DMSO, and the resulting suspension was stirred for 60 min at room temperature (*we also attempted this extraction at 65 ºC, but the difference in reaction yield was minimal*). After this time, the tube was centrifuged for 3 min at 8000 rpm. The resulting freshly extracted green supernatant (crude extract) was directly used as reaction medium. The extraction was performed adapting a reported protocol, where the concentration of chlorophyl A and B was determined to be constant for <24 h in DMSO.[3]

Unless stated otherwise, a 10 mL screw-cap glass culture tube equipped with a Teflon-coated stirring bar was charged, under air atmosphere, with a solution in 0.50 mL of DMSO of redox-active ester **1a** (0.025 mmol, 1.0 equiv), NADH (20 mol%), azauracil derivative **2b** (2.0 equiv) and 1.0 equiv of 1,3,5-trimethoxybenzene as internal standard. This was followed by the addition of 2.0 mL of freshly prepared DMSO spinach extract (final concentration of **1a** = 10 mM). The reaction tube was loaded in the sample holder of the photoreactor (see Section 2.2 for photochemical set up details). After ca. 1 min of pre-stirring, the reaction tube was irradiated with 2 red Kessil LEDs (660 nm) while stirring and cooling down with the reactor fan system. After 3 h of irradiation, the reaction tube was extracted from the photoreactor and the mixture was diluted with ca. 3 mL of EtOAc and ca. 3 mL of water and shaken energetically. The organic fraction was dried over anhydrous sodium or magnesium sulfate and centrifuged, then it was diluted in HPLC-grade DCM (ca. 1 mg/mL of **3a**) and filtered through an HPLC filter, before analyzing by GCMS to determine the yield of **3a**.

The obtained results of the reaction, and the corresponding control experiments are reported below. In all cases, yields are an average of two replicates ran on different days with different batches of spinach:

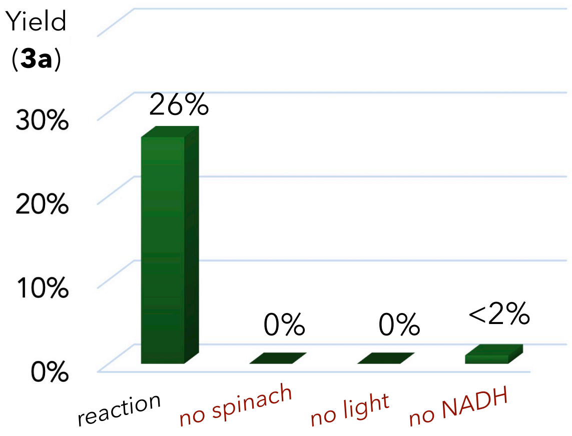

Screening the specificity of NADH as substoichiometric reductive quencher vs other biomolecules

Under aqueous conditions, we evaluated a range of biomolecules that could, in principle, function as reductive quenchers. Although the reactivity is not exclusively restricted to NADH (since certain species such as sodium ascorbate can also support productive activation) many common biomolecules proved completely inactive as cocatalysts. This contrast highlights the distinctive efficiency of NADH within this photoredox manifold.

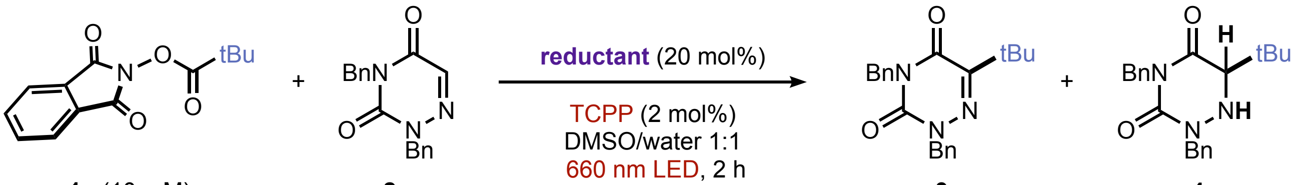

1a (10 mM) **2a** **3a** **4a**

Entry	Reductant co-catalyst	Yield **3a** (**3a**/**4a**)
1	NADH	92% (> 30:1)
2	Et_3N	n/d
4	DIPEA	n/d
6	Sodium ascorbate	94% (30:1)
7	Sodium pyruvate	n/d
8	Riboflavin	n/d
9	GSH	n/d
10	Glucose	n/d
11	Vitamin B1	n/d
12	L-cysteine	n/d
13	L-leucine	n/d
14	L-methionine	n/d
15	L-tryptophan	n/d
16	L-tyrosine	n/d

Yields of **3a** were determined by GC–MS using 1,3,5-trimethoxybenzene as internal standard.
3**a/4a r**atios are the relative integrals of both products. n/d = not detected

Table S8. Alternative biomolecules as potential reductive quenchers.

8. Photophysical studies

8.1. UV–Vis absorption spectroscopy

General information

UV–Vis absorption spectroscopy was performed using a Jasco V-770 UV–Vis spectrophotometer at room temperature (ca. 25 ºC). Sample solutions of the appropriate concentration were freshly prepared in HPLC-grade DMSO. Absorption measurements were performed under air either in 10×2 mm disposable plastic cuvettes (l = 10 mm) or in 10×10 mm quartz cuvettes (l = 10 mm).

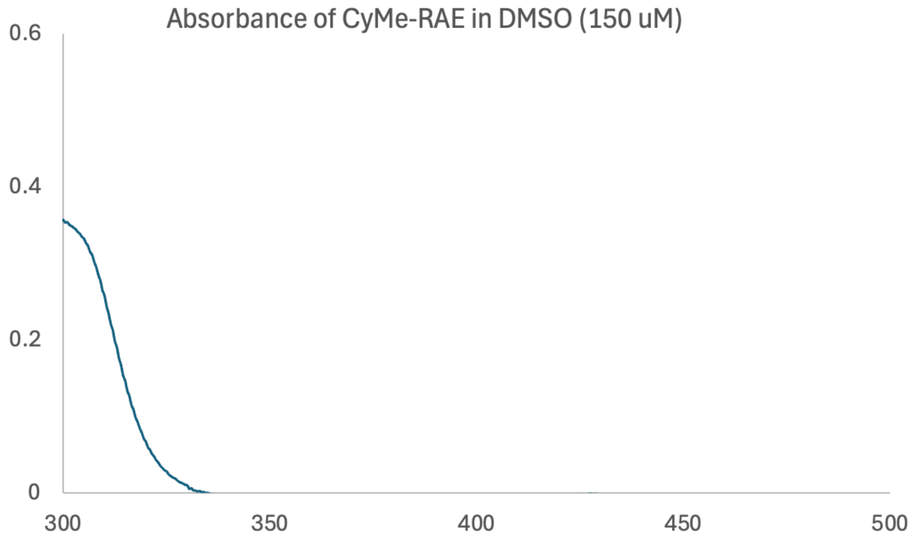

Figure S7. UV–Vis absorption spectrum of RAE **1a** in DMSO (150 μM).

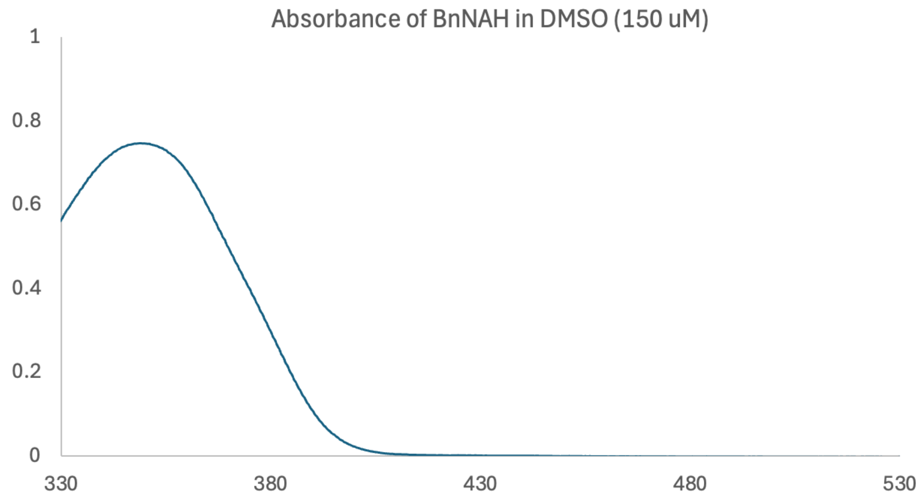

Figure S8. UV–Vis absorption spectrum of BNAH in DMSO (150 μM).

Neither the reaction substrate nor the reactant absorb at the wavelengths of emission of the two LEDs used in this study, justifying the requirement of photocatalysts. In contrast, BNAH (yellow solid) absorption tails into the blue-LED emission range, justifying reactivity through direct blue-light excitation.

8.2. Transient-absorption spectroscopy (TAS)

General considerations for TAS

Ultrafast transient absorption experiments were conducted using an Astrella-F-1K amplified Ti:sapphire femtosecond laser system from Coherent, operating at a repetition rate 1kHz, 5.5 W power (5 mJ pulse energy), pulse duration of 80 fs. TA pump / EOS (nanosecond TAS) detection system from Ultrafast Systems was utilized. White light (~370 to >1600 nm) was generated by a built-in photonic crystal fiber supercontinuum laser source with a fundamental of 1064 nm at 2 kHz output frequency and pulse width of approximately 1 ns. A 1.2 mJ fraction of the fundamental was used for pump beam generation by a TOPAS Prime from Light Conversion with standard NirUVis extension. A depolarizer was placed in the pump beam to avoid rotational dynamics. Bandpass filters with ± 5 or ± 10 nm were used to ensure low spectral width and to exclude 800 nm photons. Pump energy was around 1000 nJ per pulse. Excitation spot diameters were typically 150-200 μm (1/e) (affording pump fluences around 5-10 mJ/cm^2) and were ensured to be larger than those of the probe beam. All measurements were conducted in a 2 mm quartz cuvette under argon atmosphere, using solutions with absorbances of 0.5-0.7. To analyze transient absorption data, we used a suggested procedure.[4] We start with SVD and global analysis, using an all-sequential decay model that provides evolution associated spectra of potentially intervening species, to determine the number of decaying species that participate in the decay cascade. However, this doesn't necessary yield differential spectra with genuine physicochemical meaning. Afterwards, a target analysis is applied, using specific target models that result in species associated spectra with true physicochemical meaning. Obtained data were treated by SVD, global and target analyses using the R- package TIMP and GloTarAn.[4,5]

Transient-absorption spectroscopy study

Insights into the photochemical mechanism were gathered using nanosecond TAS, under 650 nm excitation. We started with TPP in DMSO, which shows the typical excited-state dynamics of free-base porphyrins (Figure S9). The lowest singlet excited state S_1, showing a differential spectrum with maxima at 440, 533, 568 and 614 nm, decays in 11.4 ns and populates the lowest triplet. Upon intersystem crossing, excited-state absorptions at $\lambda > 500$ nm appear less intense, but do not shift appreciably. At higher energies, the peak at 440 nm intensifies and shifts to 442. T_1 decays to the ground state in 50.0 μs. Upon addition of BNAH, a porphyrin-based longer-lived species is observed in TAS (Figure 5B and S10). This species, with a lifetime longer than the time window of our experiment, features a differential spectrum with clearly distinct bands at 454 and 875 nm. Notably, these excited-state absorptions grow hand in hand with the decay of the triplet state, without any evidence of them at shorter timescales. These peaks are characteristic for the radical anionic form of TPP and therefore are clear evidence of a BNAH-to-TPP electron transfer process producing a $\{TPP^{\bullet-}\cdot BNAH^{\bullet+}\}$ charge separated state CSS.[6] Like in the literature, no clear optical signatures of $BNAH^{\bullet+}$ were observed.[7] A similar behavior was observed when NADH was added to TPP (Figure S11), with an excited-state decay cascade including S_1, T_1 and the CSS. This confirms that BNAH is an excellent model for NADH.

Stern–Volmer quenching experiments were performed to study the bimolecular reactivity of TPP excited states with BNAH (Figures 5C and S12). Rate constants for S_1 decay upon addition of increasing amounts of BNAH show a linear Stern–Volmer plot, indicating dynamic diffusional quenching with a bimolecular rate constant of 1.1×10^9 $M^{-1}s^{-1}$, one order of magnitude smaller than the diffusion limit in DMSO of 1.9×10^{10} $M^{-1}s^{-1}$. Curiously, the product of this quenching process is not observed at any of the quencher concentrations utilized. We postulate that S_1 engages in photoinduced electron transfer with BNAH, and that the charge-separated product recombines, in a faster timescale, forming T_1, similar to other donor–acceptor assemblies.[8] On the other hand, T_1 shows a curved Stern-Volmer plot, which was fitted using a model that considers an equilibrium between T_1 and a charge-separated state with triplet multiplicity, ^{3}CSS, which also decays to the ground state (Figure S13). This state corresponds to our observations of $TPP^{\bullet-}$ spectral signatures at long time delays.

Next, bimolecular reactivity of TPP excited states with RAE was investigated, in presence or absence of BNAH (Figures 5C and S12). Stern–Volmer experiments in the absence of BNAH confirmed no significant impact of increasing concentrations of RAE on the excited-state dynamics of TPP. When 4 mM of BNAH were present, addition of RAE altered the dynamics only at time delays around 100 μs, while singlet and triplet dynamics remained unaffected (Figure S14). This is consistent with a bimolecular reaction of RAE with the ^{3}CSS. Unfortunately, further characterization of the products of this reaction was limited by the time-window available for our TAS experiment.

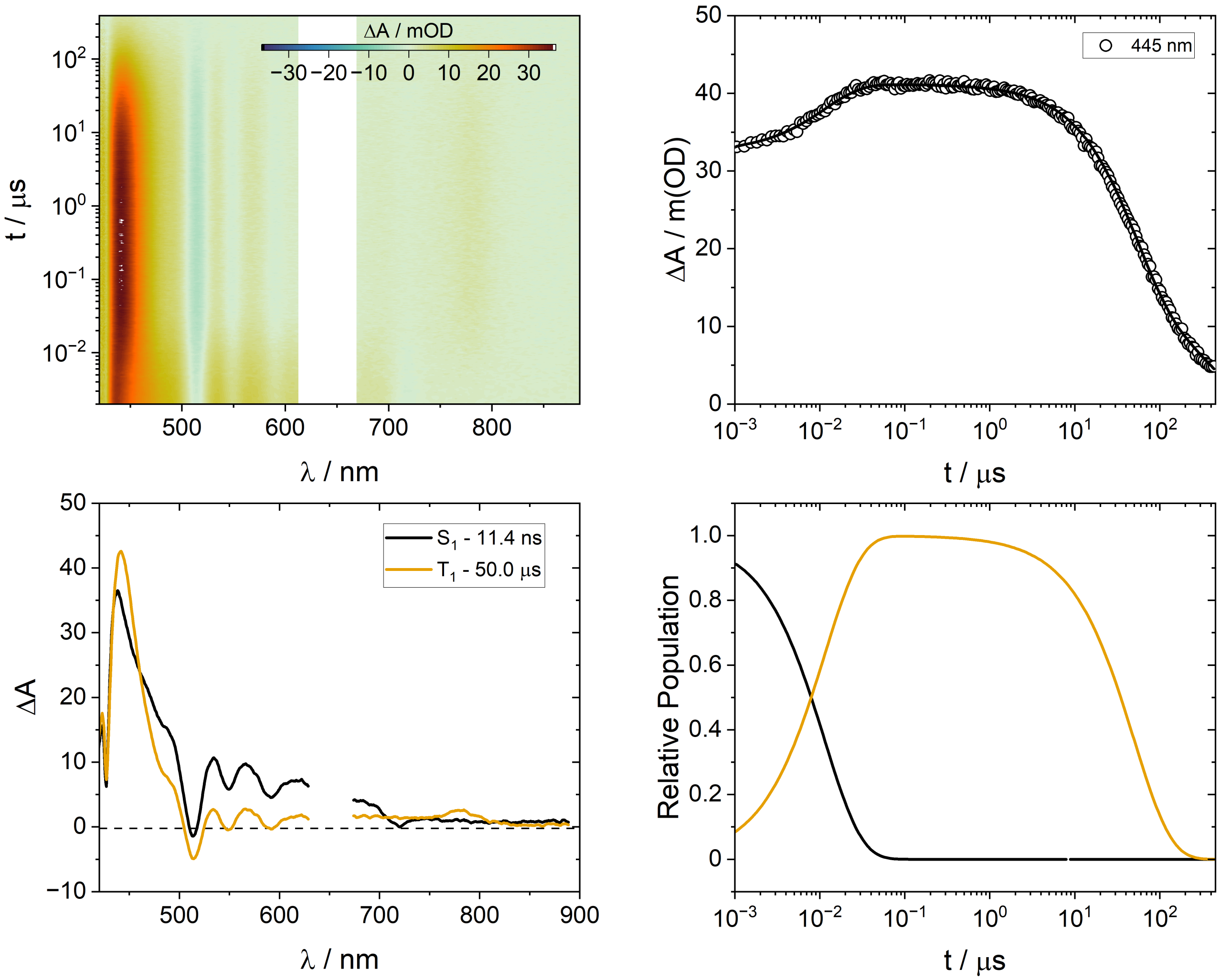

Figure S9. Top left: Nanosecond TAS of TPP in DMSO under 650 nm excitation at room temperature. Top right: Kinetic evolution of the differential absorbance at 445 nm (dots) and corresponding fit (line). Bottom left: Species-associated differential spectra of S_1 (black) and T_1 (orange) states. Bottom right: Population evolution over time.

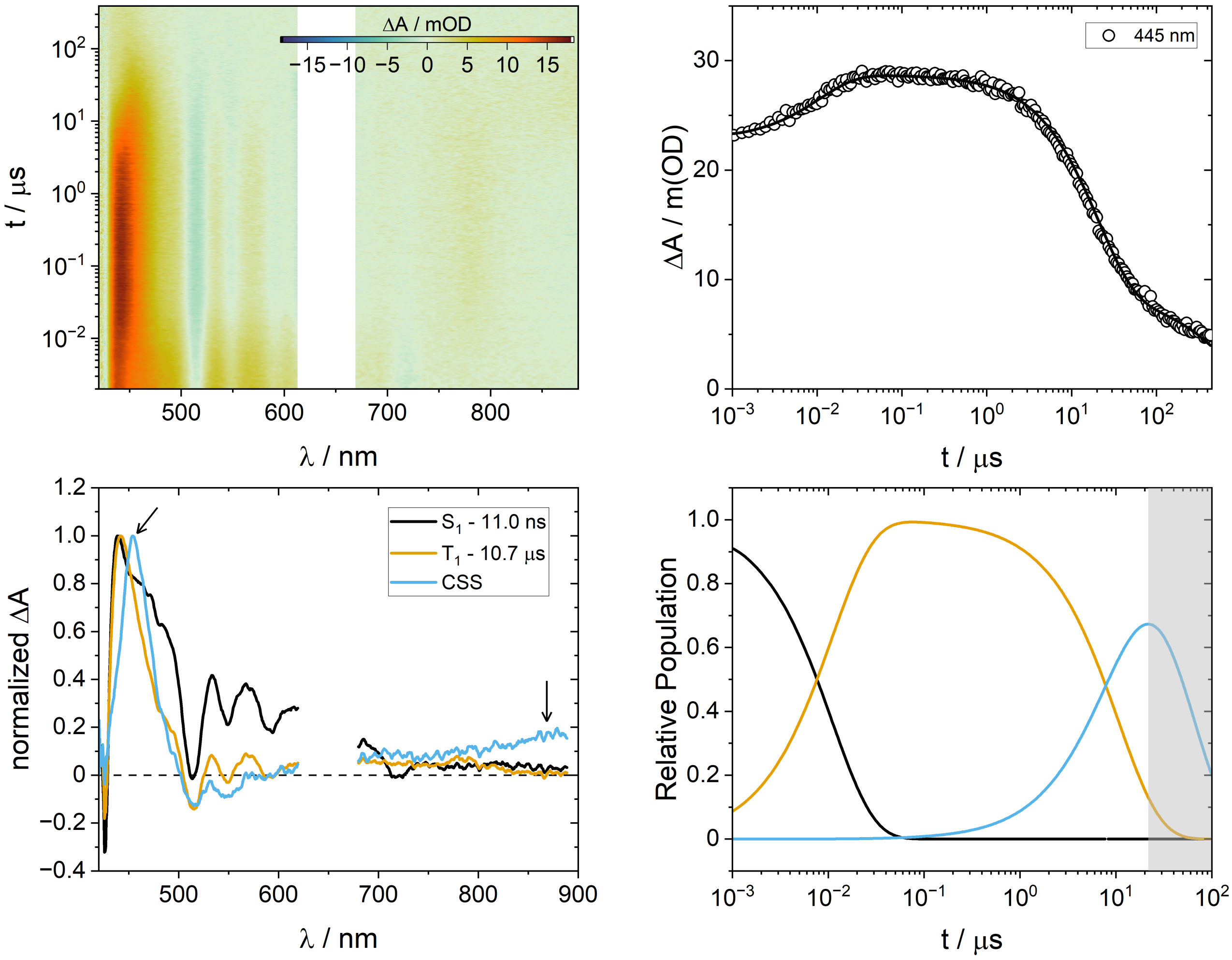

Figure S10. Top left: Nanosecond TAS of TPP in DMSO under 650 nm excitation at room temperature, in the presence of 4 mM BNAH. Top right: Kinetic evolution of the differential absorbance at 445 nm (dots) and corresponding fit (line). Bottom left: Species-associated differential spectra of S_1 (black), T_1 (orange) and CSS (blue) states. Bottom right: Population evolution over time. At long time delays (shaded gray area), bimolecular charge recombination between $TPP^{\bullet -}$ and $BNAH^{\bullet +}$ is not exponential, so the population evolution of the CSS should be taken with care.

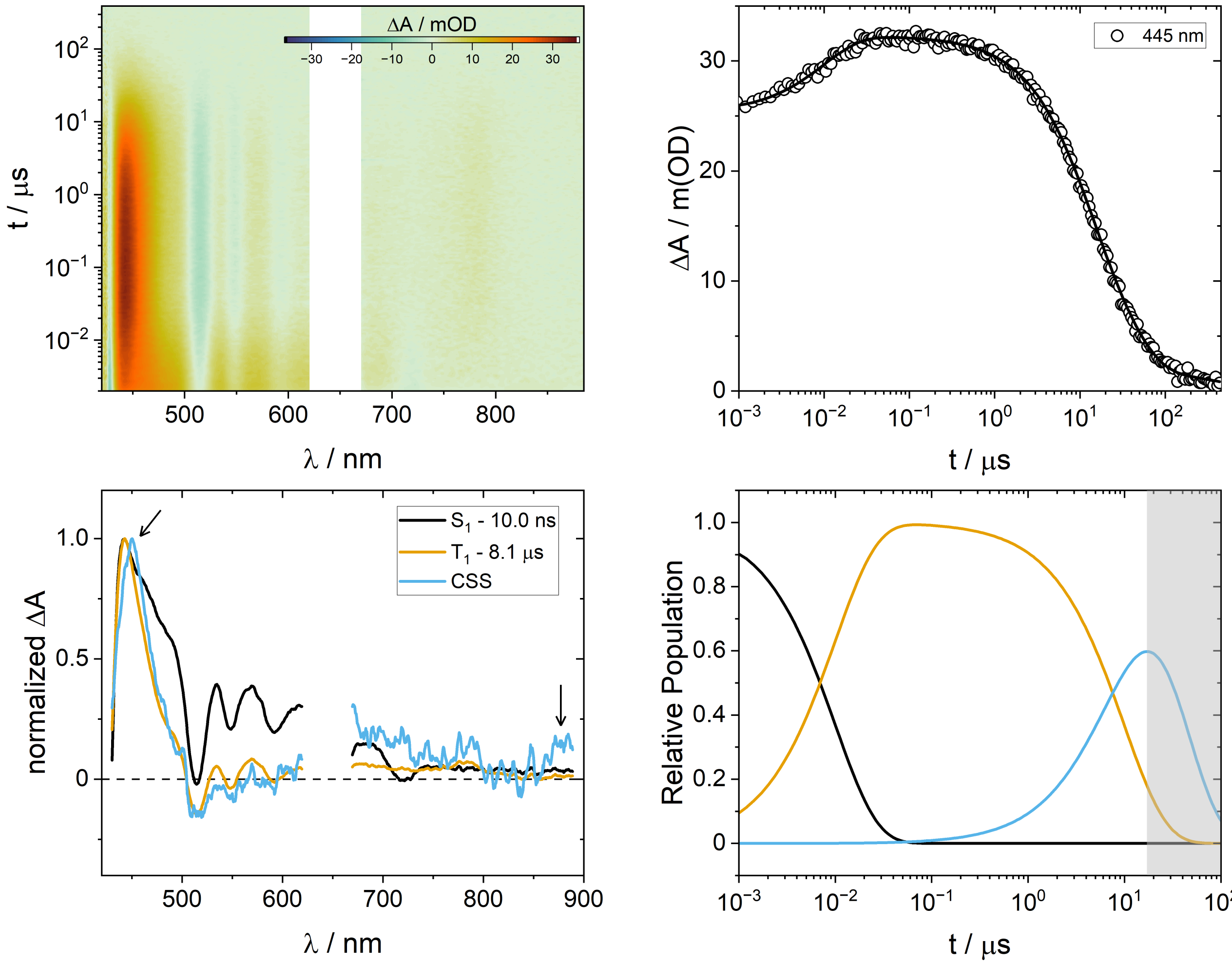

Figure S11. Top left: Nanosecond TAS of TPP in DMSO under 650 nm excitation at room temperature, in the presence of 10 mM NADH. Top right: Kinetic evolution of the differential absorbance at 445 nm (dots) and corresponding fit (line). Bottom left: Species-associated differential spectra of S_1 (black), T_1 (orange) and CSS (blue) states. Bottom right: Population evolution over time. At long time delays (shaded gray area), bimolecular charge recombination between $TPP^{\bullet-}$ and $NADH^{\bullet+}$ is not exponential, so the population evolution of the CSS should be taken with care.

Stern–Volmer quenching experiments of TPP with RAE and BNAH

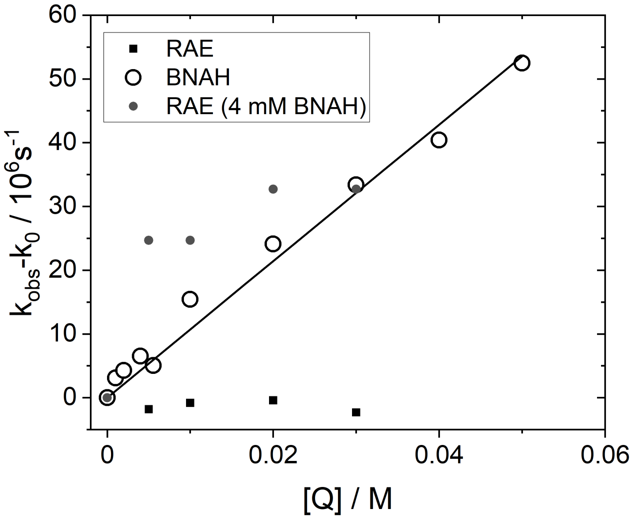

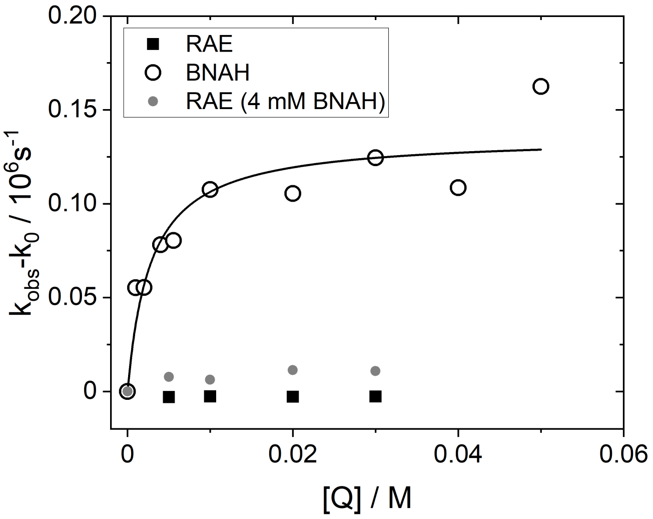

Figure S12. Stern-Volmer analyses for the singlet (left) and triplet (right) states of TPP in the presence of BNAH and RAE.

Curved Stern-Vomer Model with Equilibrium

This model considers that T_1 and ^{3}CSS equilibrate in a faster timescale than their individual decays to the ground state. k_1 was fixed to its value in absence of quencher, 1.55 x 10^4 s^{-1}. This afforded k_3 = 1.52 x 10^5 s^{-1}, and k_2 and k_{-2} around 10^6 s^{-1}. Large uncertainties were obtained, indicating that this modelling might not fully capture the real picture. However, since we are not deriving quantitative information from this experiment, we believe it is still valuable as it shows complex dynamics that go beyond simple diffusional quenching.

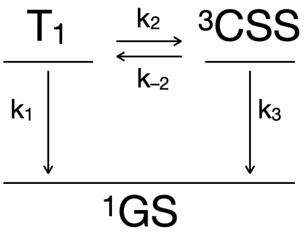

$$k_{obs}([Q]) = \frac{k_{-2}k_1 + k_2[Q]\,k_3}{k_{-2} + k_2[Q]}$$

Figure S13. Equilibrium decay model and corresponding fitting equation.

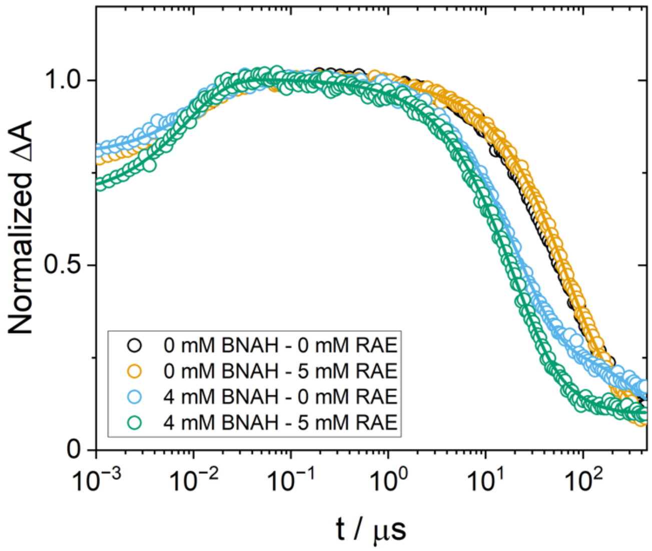

Figure S14. Transient-absorption kinetic traces at 445 nm (dots) and fits (lines) of TPP in DMSO under 650 nm excitation, with different concentrations of BNAH and RAE.

9. Mechanistic experiments

9.1. Effect of reaction components

Unless stated otherwise, all reaction development was carried out using the standard protocols described in General Procedures C, under the correspondingly modified reaction conditions as described in this section. Unless stated otherwise, for product **3a**, in all optimization, development or control reactions, yields were determined by GCMS (for details, see Section 6).

Kinetic analysis

Initially, we built an overall kinetic profile analyzing aliquots of a standard reaction after different reaction times.

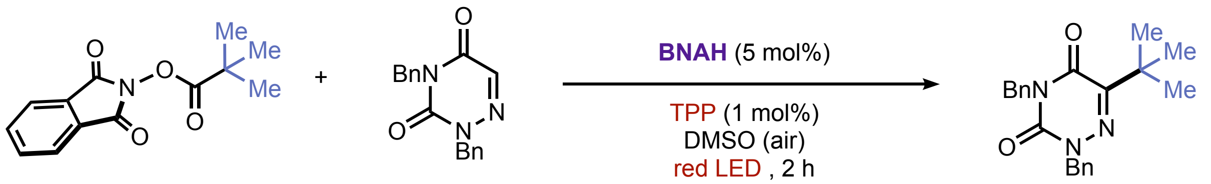

(1.0 equiv, 25 mM) (2.0 equiv)

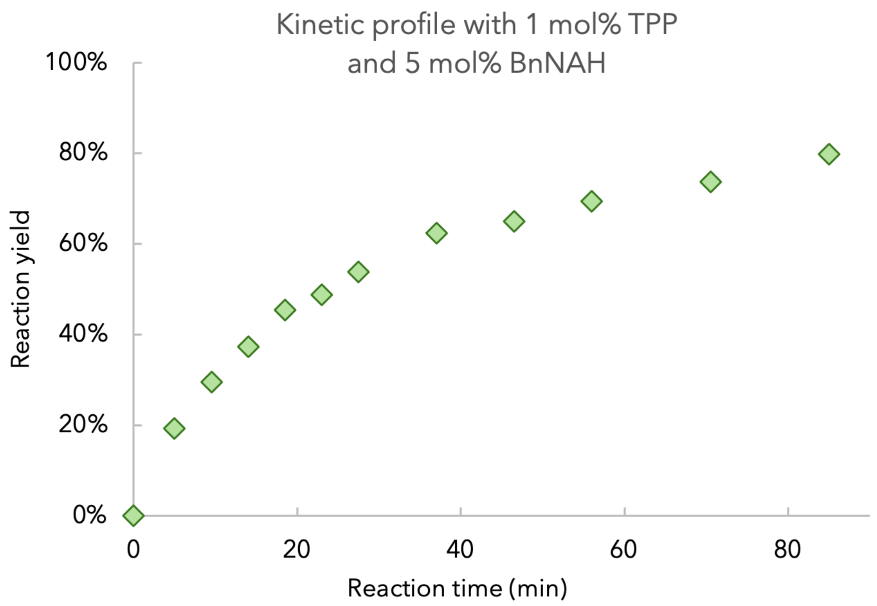

Figure S15. Kinetic profile of the reaction under standard conditions.

Then, we moved on to explore the effect of modulating the concentration of the different key components of the reaction and their effect on the rate. To simplify kinetic analysis, we evaluated reaction rate as a function of the yield of **3a** obtained after 1 h of reaction, always within concentration ranges below high conversion.

Effect of [BNAH] on reaction rate

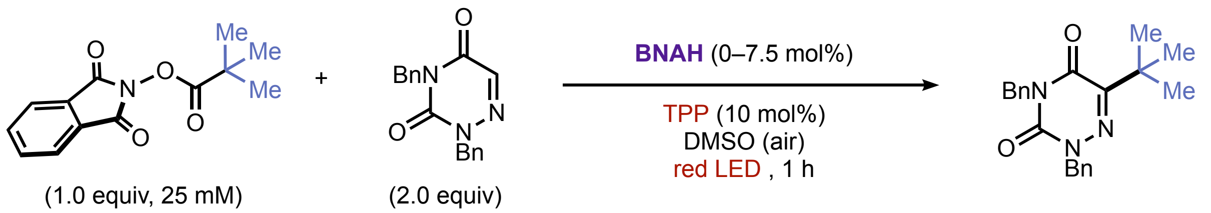

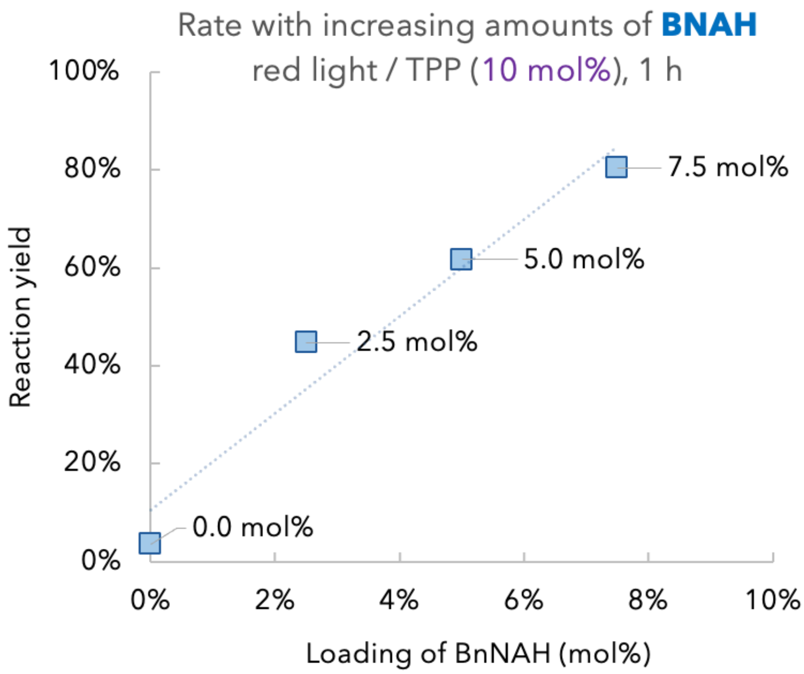

Figure S16. Effect of [BNAH] in reaction yield after 1 h.

Effect of [TPP] on reaction rate

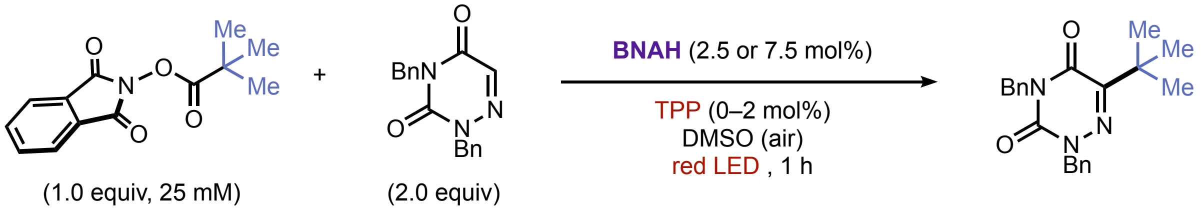

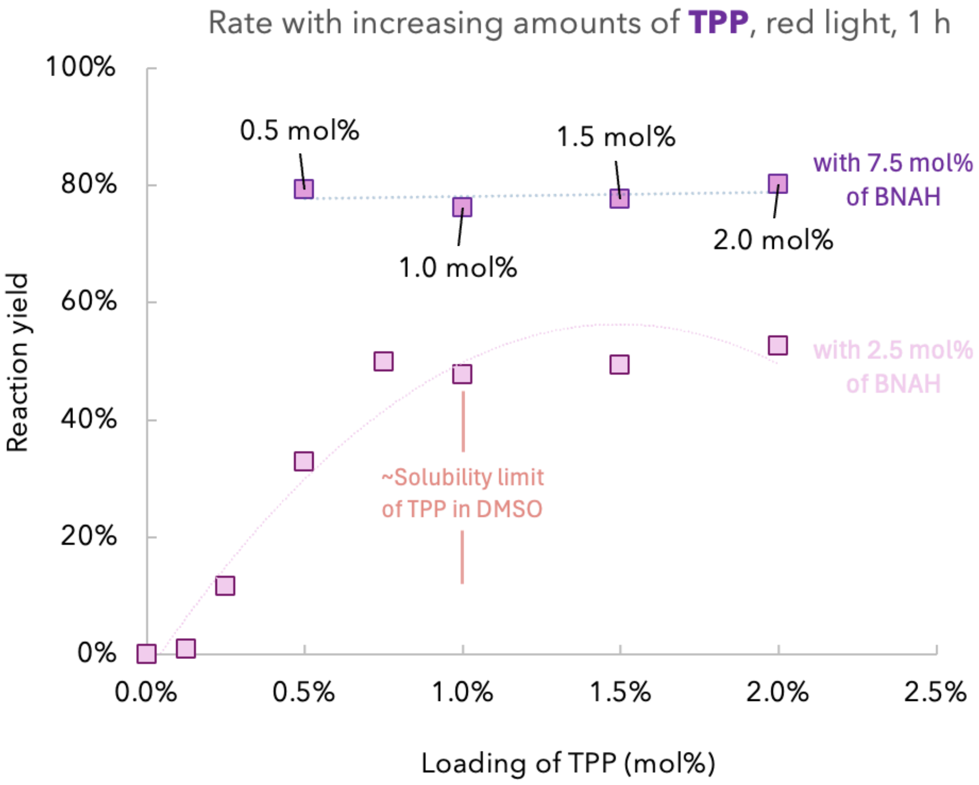

Figure S17. Effect of [TPP] in reaction yield after 1 h. **Note**: at 7.5 mol% of BNAH, the reaction was too fast to evaluate the effect of [TPP].

Effect of light intensity on reaction rate

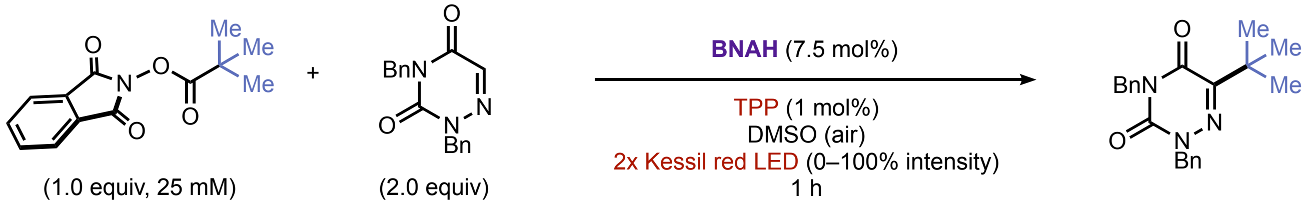

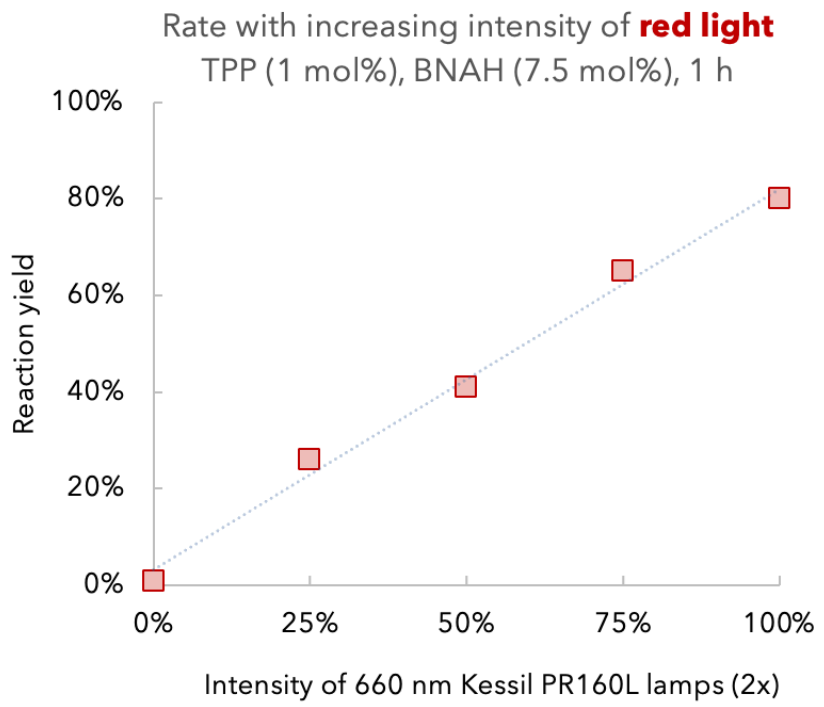

Figure S18. Effect of red-light intensity in reaction yield after 1 h.

Intermittent irradiation (on/off) experiments

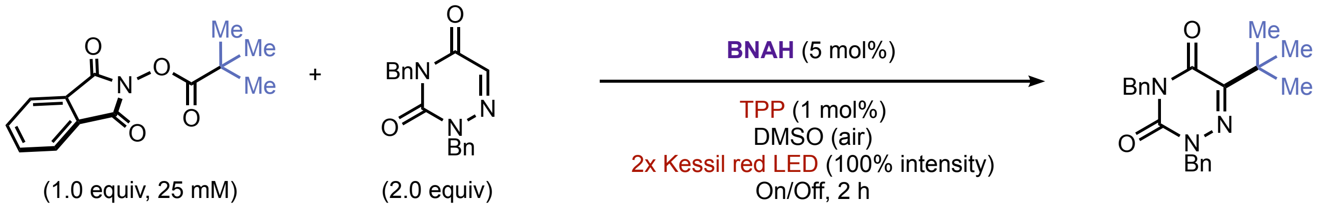

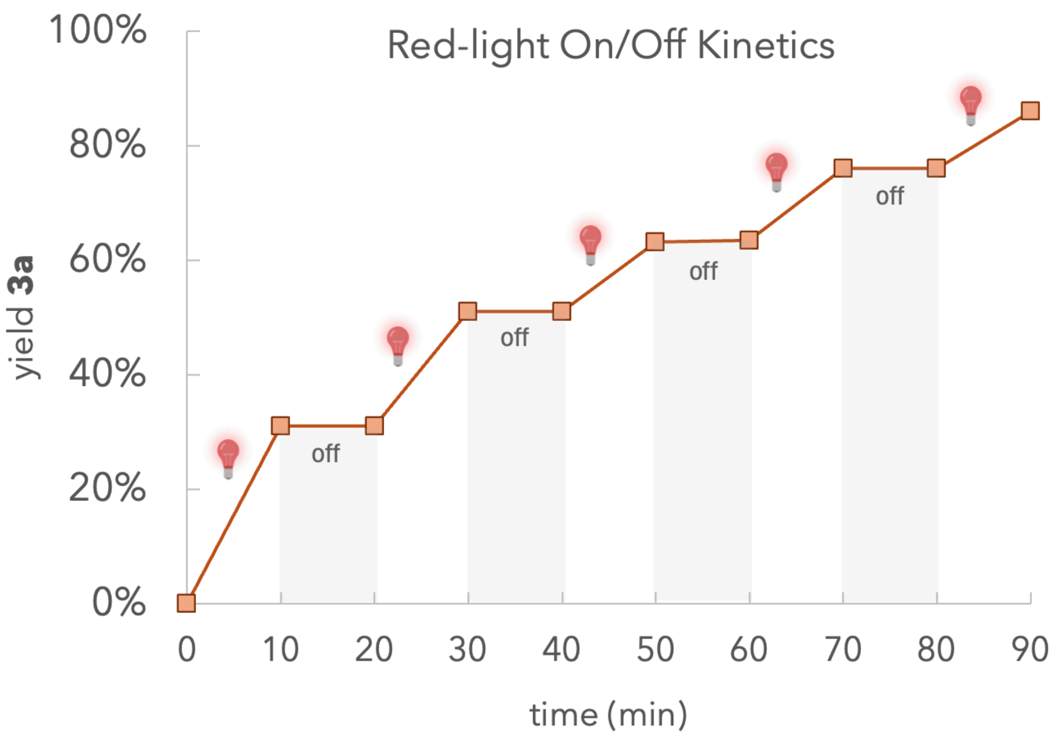

Figure S19. Intermittent irradiation (on/off) experiments.

9.2. Red-light photocatalysis vs blue-light direct excitation

BNAH (a yellow compound) is known to be photoactive under direct blue-light irradiation, and should be able to activate RAEs via SET.[1c] Thus, we tested the model reaction in the absence of photocatalyst, under 456 nm light irradiation, and with several loadings of BNAH. This led to only stoichiometric reactivity, and we did not observe any catalytic or turnover-yielding effect via radical chain propagation.

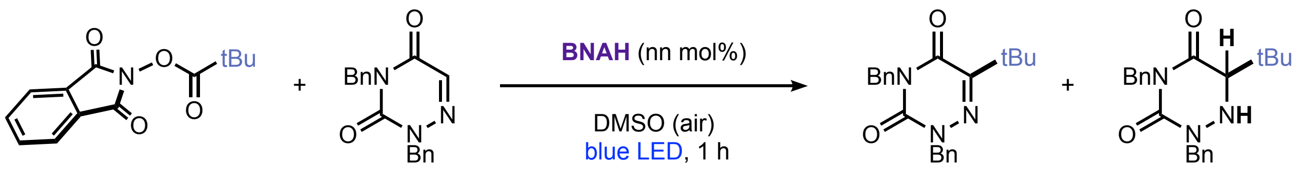

1a (25 mM) **2a** **3a** **4a**

Entry	Reductant co-catalyst	Yield **3a** (**3a**/**4a**)
1	without BNAH	n/d
2	BNAH (10 mol%)	5% (>20:1)
3	BNAH (20 mol%)	23% (>20:1)
4	BNAH (30 mol%)	45% (>20:1)
5	BNAH (40 mol%)	51% (>20:1)
6	BNAH (50 mol%)	61% (>20:1)
7	BNAH (60 mol%)	71% (>20:1)
8	BNAH (150 mol%)	76% (9:1)

Yields of **3a** were determined by GC–MS using 1,3,5-trimethoxybenzene as internal standard.
3a/4a ratios are the relative integrals of both products. n/d = not detected

Table S9. Effect of [BNAH] on the reaction under direct blue-light excitation.

Below we plotted the reaction yield of the model reaction as a function of the amount of BNAH loading, for both the red-light photocatalytic system (left) and the photocatalyst-free blue-light system (right). The latter gives a slope of ~1, meaning that 1 equiv of BNAH leads to only ~1 equiv of product. The former gives a slope an order of magnitude larger, corresponding to at least 10 turnover events.

Even though completely ruling out a simple free-radical chain propagation is very challenging, this data does not suggest this scenario: the blue-light system should also be able to initiate such propagation by itself. However, no turnover is observed, thus suggesting that either the TPP photocatalyst is directly involved in the main mechanistic pathway. Alternatively, the TPP/BNAH combination might be promoting the initiation of short radical chains.

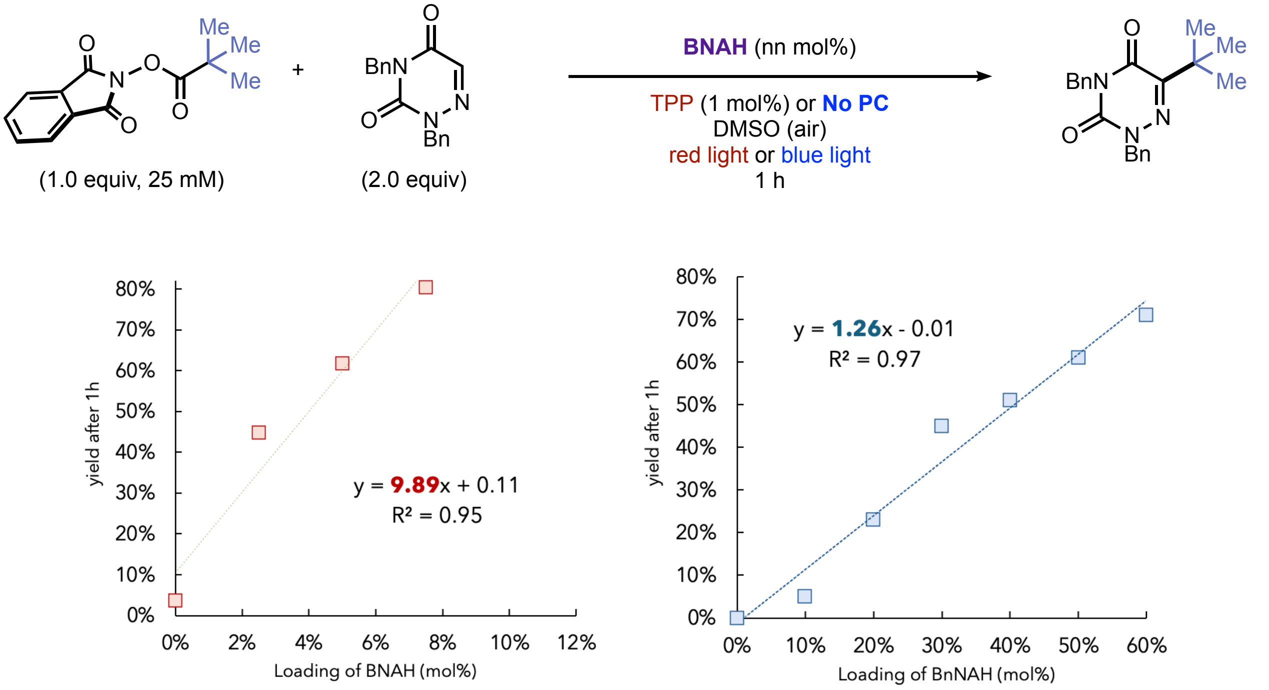

Figure S20. Effect of [BNAH] on rate under either red-light irradiation (in the presence of a photocatalyst, 1 mol% of TPP, top left) vs blue-light irradiation (direct excitation, top right).

Overall, while the present study confirms that substoichiometric amounts of BNAH are able to promote this redox-neutral reactivity under red-light irradiation in the presence of TPP, when monitoring the reaction by ^{1}H NMR in DMSO-d_6 under standard conditions, we did not observe recovery of unreacted BNAH. Instead, complex speciation is observed, and at this point, the exact role of the reductant beyond behaving as substoichiometric reductive quencher is unclear.

While some circumstantial evidence, such as the absence of turnover upon simple blue-light activation of BNAH, points towards some kind of productive co-catalytic interaction between TPP and BNAH, the reductant could also be acting as precatalyst or as a substoichiometric initiator.

11. Crystal data and structure refinement

3-(*tert*-Butyl)-2-phenyl-2*H*-indazole (3n)

Single crystals of **3n** were obtained by slow evaporation (ca. 24 h) of a solution of **3n** (25 mg) in a solvent mixture of $CDCl_3$ (0.6 mL) and MeCN (1.0 mL) in an uncapped 6 mL glass vial at room temperature over 24 h.

CCDC deposit number: 2513057

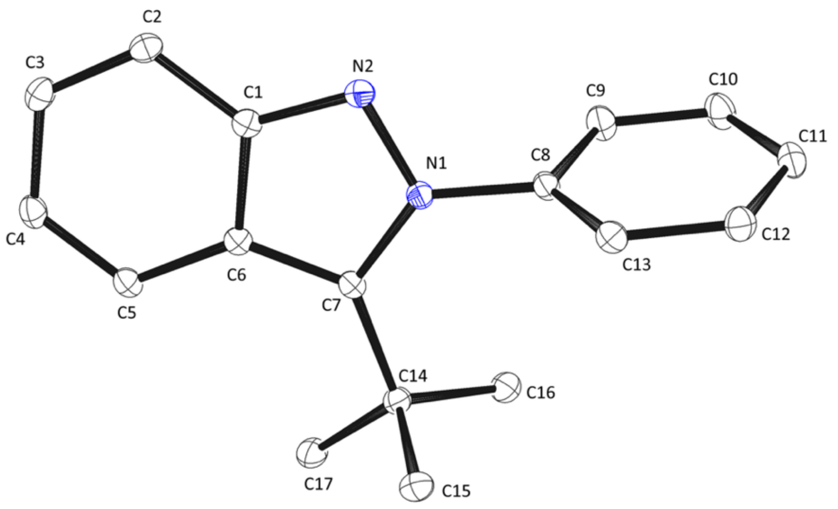

Figure S21. The molecular structure of **3n** from single-crystal X-ray diffraction analysis. H atoms have been removed for clarity. Ellipsoids drawn at 50% probability level.

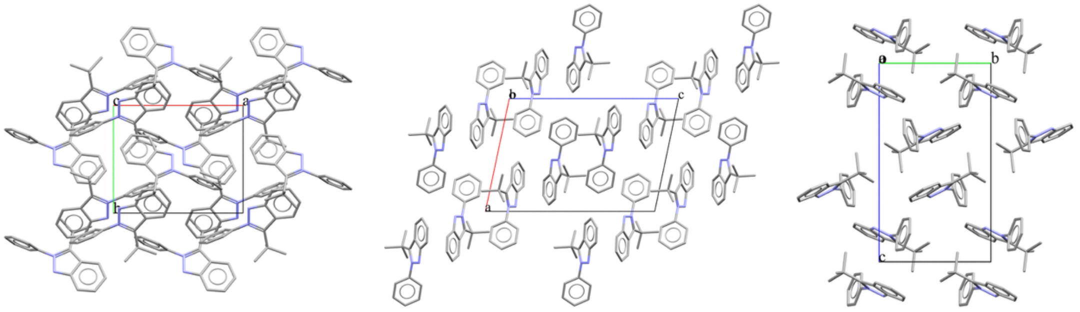

Figure S22. Packing of **3n** (ab left; ac middle; bc right).

Experimental details for x-ray analysis of **3n**

Experimental details

Crystal data	
Chemical formula	$C_{17}H_{18}N_2$
M_r	250.33
Crystal system, space group	Monoclinic, $P2_1/n$
Temperature (K)	100
a, *b*, *c* (Å)	10.5265 (1), 8.5385 (1), 15.4907 (2)
β (°)	101.963 (1)
V (Å^3)	1362.07 (3)
Z	4
Radiation type	Cu *K*α
μ (mm^{-1})	0.55
Crystal size (mm)	0.11 × 0.09 × 0.01
Data collection	
Diffractometer	XtaLAB Synergy, Dualflex, HyPix-Arc 150
Absorption correction	Multi-scan<br>*CrysAlis PRO* 1.171.44.118a (Rigaku Oxford Diffraction, 2025) Empirical absorption correction using spherical harmonics, implemented in SCALE3 ABSPACK scaling algorithm.
T_{min}, T_{max}	0.950, 1
No. of measured, independent and observed [$I > 2\sigma(I)$] reflections	21129, 2561, 2372
R_{int}	0.027
$(\sin \theta/\lambda)_{max}$ (Å^{-1})	0.610
Refinement	
$R[F^2 > 2\sigma(F^2)]$, $wR(F^2)$, S	0.035, 0.087, 1.06
No. of reflections	2561
No. of parameters	175
H-atom treatment	H-atom parameters constrained
$\Delta\rho_{max}$, $\Delta\rho_{min}$ (e Å^{-3})	0.21, −0.2

Computer programs: *CrysAlis PRO* 1.171.44.118a (Rigaku OD, 2025), *SHELXT* 2018/2 (Sheldrick, 2018), *SHELXL* 2019/3 (Sheldrick, 2015), Olex2 1.5-ac7-014 (Dolomanov *et al.*, 2009).

Computing details

Data collection: *CrysAlis PRO* 1.171.44.118a (Rigaku OD, 2025); cell refinement: *CrysAlis PRO* 1.171.44.118a (Rigaku OD, 2025); data reduction: *CrysAlis PRO* 1.171.44.118a (Rigaku OD, 2025); program(s) used to solve structure: *SHELXT* 2018/2 (Sheldrick, 2018); program(s) used to refine structure: *SHELXL* 2019/3 (Sheldrick, 2015); molecular graphics: Olex2 1.5-ac7-014 (Dolomanov *et al.*, 2009); software used to prepare material for publication: Olex2 1.5-ac7-014 (Dolomanov *et al.*, 2009).

Crystal data

$C_{17}H_{18}N_2$
M_r = 250.33
Monoclinic, $P2_1/n$
Hall symbol: -P 2yn
a = 10.5265 (1) Å
b = 8.5385 (1) Å
c = 15.4907 (2) Å
β = 101.963 (1)°
V = 1362.07 (3) Å^3
Z = 4
$F(000)$ = 536
D_x = 1.221 Mg m^{-3}
Cu $K\alpha$ radiation, λ = 1.54184 Å
Cell parameters from 13235 reflections
θ = 4.7–74.4°
μ = 0.55 mm^{-1}
T = 100 K
Plate, clear light colourless
0.11 × 0.09 × 0.01 mm

Data collection

XtaLAB Synergy, Dualflex, HyPix-Arc 150 diffractometer
Radiation source: micro-focus sealed X-ray tube, PhotonJet (Cu) X-ray Source
Mirror monochromator
Detector resolution: 10 pixels mm^{-1}
ω scans
Absorption correction: multi-scan *CrysAlis PRO* 1.171.44.118a (Rigaku Oxford Diffraction, 2025) Empirical absorption correction using spherical harmonics, implemented in SCALE3 ABSPACK scaling algorithm.
T_{min} = 0.950, T_{max} = 1
21129 measured reflections
2561 independent reflections
2372 reflections with $I > 2\sigma(I)$
R_{int} = 0.027
θ_{max} = 70.1°, θ_{min} = 4.7°
h = −12→12
k = −9→7
l = −18→18

Refinement

Refinement on F^2
Least-squares matrix: full
$R[F^2 > 2\sigma(F^2)]$ = 0.035
$wR(F^2)$ = 0.087
S = 1.06
2561 reflections
175 parameters
0 restraints
0 constraints
Primary atom site location: dual
Hydrogen site location: inferred from neighbouring sites
H-atom parameters constrained
$w = 1/[\sigma^2(F_o^2) + (0.0407P)^2 + 0.454P]$ where $P = (F_o^2 + 2F_c^2)/3$
$(\Delta/\sigma)_{max}$ = 0.001
$\Delta\rho_{max}$ = 0.21 e Å^{-3}
$\Delta\rho_{min}$ = −0.2 e Å^{-3}

Special details

Geometry. All e.s.d.'s (except the e.s.d. in the dihedral angle between two l.s. planes) are estimated using the full covariance matrix. The cell e.s.d.'s are taken into account individually in the estimation of e.s.d.'s in distances, angles and torsion angles; correlations between e.s.d.'s in cell parameters are only used when they are defined by crystal symmetry. An approximate (isotropic) treatment of cell e.s.d.'s is used for estimating e.s.d.'s involving l.s. planes.

Table S10. Fractional atomic coordinates and isotropic or equivalent isotropic displacement parameters ($Å^2$)

	x	*y*	*z*	U_{iso}*/U_{eq}
N1	0.45649 (8)	0.59895 (10)	0.63185 (6)	0.0170 (2)
N2	0.45560 (9)	0.45646 (10)	0.67153 (6)	0.0193 (2)
C1	0.32854 (10)	0.42486 (12)	0.66298 (7)	0.0177 (2)
C2	0.27480 (11)	0.28774 (12)	0.69273 (7)	0.0205 (2)
H2	0.329017	0.207046	0.722359	0.025*
C3	0.14310 (11)	0.27538 (13)	0.67751 (7)	0.0226 (2)
H3	0.104761	0.184286	0.696401	0.027*
C4	0.06183 (11)	0.39710 (13)	0.63363 (7)	0.0223 (2)
H4	−0.029749	0.385546	0.62448	0.027*
C5	0.11184 (10)	0.52992 (13)	0.60435 (7)	0.0201 (2)
H5	0.055905	0.609409	0.575062	0.024*
C6	0.24901 (10)	0.54721 (12)	0.61843 (7)	0.0171 (2)
C7	0.33636 (10)	0.66095 (12)	0.59925 (6)	0.0167 (2)
C8	0.58454 (10)	0.66099 (12)	0.63377 (7)	0.0182 (2)
C9	0.65507 (11)	0.71773 (14)	0.71329 (7)	0.0245 (3)
H9	0.618609	0.716521	0.764483	0.029*
C10	0.77899 (11)	0.77620 (14)	0.71759 (8)	0.0268 (3)
H10	0.827361	0.816821	0.771579	0.032*
C11	0.83237 (11)	0.77524 (13)	0.64267 (8)	0.0246 (3)
H11	0.916904	0.816538	0.645368	0.03*
C12	0.76278 (11)	0.71431 (13)	0.56412 (7)	0.0224 (2)
H12	0.800337	0.712366	0.513409	0.027*
C13	0.63799 (10)	0.65591 (12)	0.55920 (7)	0.0202 (2)
H13	0.590208	0.61325	0.505572	0.024*
C14	0.31177 (10)	0.81838 (12)	0.55216 (7)	0.0193 (2)
C15	0.31990 (11)	0.79834 (13)	0.45466 (7)	0.0241 (3)
H15A	0.408831	0.770094	0.450918	0.036*
H15B	0.295791	0.896871	0.423054	0.036*
H15C	0.260247	0.715303	0.427961	0.036*
C16	0.40802 (11)	0.94513 (13)	0.59565 (8)	0.0243 (3)
H16A	0.409421	0.948834	0.659073	0.037*
H16B	0.380838	1.047165	0.569174	0.037*
H16C	0.495072	0.920111	0.586402	0.037*
C17	0.17455 (11)	0.87715 (13)	0.55532 (8)	0.0253 (3)
H17A	0.109832	0.80907	0.518952	0.038*
H17B	0.163743	0.984511	0.532615	0.038*
H17C	0.162728	0.875291	0.616425	0.038*

Atomic displacement parameters ($Å^2$)

	U^{11}	U^{22}	U^{33}	U^{12}	U^{13}	U^{23}
N1	0.0160 (4)	0.0184 (4)	0.0166 (4)	−0.0006 (3)	0.0031 (3)	0.0003 (3)
N2	0.0200 (5)	0.0192 (5)	0.0183 (4)	0.0009 (3)	0.0032 (4)	0.0020 (3)

C1	0.0194 (5)	0.0197 (5)	0.0144 (5)	0.0008 (4)	0.0047 (4)	−0.0015 (4)
C2	0.0246 (6)	0.0195 (5)	0.0181 (5)	0.0014 (4)	0.0062 (4)	0.0012 (4)
C3	0.0261 (6)	0.0220 (6)	0.0214 (5)	−0.0044 (4)	0.0089 (4)	0.0004 (4)
C4	0.0175 (5)	0.0286 (6)	0.0214 (5)	−0.0035 (4)	0.0050 (4)	−0.0015 (4)
C5	0.0172 (5)	0.0235 (5)	0.0194 (5)	0.0009 (4)	0.0031 (4)	−0.0007 (4)
C6	0.0183 (5)	0.0189 (5)	0.0146 (5)	0.0005 (4)	0.0043 (4)	−0.0017 (4)
C7	0.0157 (5)	0.0195 (5)	0.0151 (5)	0.0009 (4)	0.0034 (4)	−0.0014 (4)
C8	0.0147 (5)	0.0191 (5)	0.0203 (5)	0.0003 (4)	0.0027 (4)	0.0002 (4)
C9	0.0209 (6)	0.0340 (6)	0.0187 (5)	−0.0028 (4)	0.0046 (4)	−0.0034 (4)
C10	0.0210 (6)	0.0359 (7)	0.0219 (6)	−0.0047 (5)	0.0008 (4)	−0.0049 (5)
C11	0.0167 (5)	0.0275 (6)	0.0294 (6)	−0.0023 (4)	0.0045 (4)	−0.0010 (4)
C12	0.0208 (5)	0.0252 (6)	0.0232 (6)	0.0016 (4)	0.0089 (4)	0.0000 (4)
C13	0.0200 (5)	0.0209 (5)	0.0193 (5)	0.0002 (4)	0.0032 (4)	−0.0020 (4)
C14	0.0195 (5)	0.0187 (5)	0.0200 (5)	0.0005 (4)	0.0046 (4)	0.0021 (4)
C15	0.0262 (6)	0.0262 (6)	0.0198 (6)	0.0004 (4)	0.0046 (4)	0.0040 (4)
C16	0.0261 (6)	0.0197 (6)	0.0266 (6)	−0.0014 (4)	0.0039 (5)	0.0012 (4)
C17	0.0227 (6)	0.0219 (6)	0.0320 (6)	0.0040 (4)	0.0074 (5)	0.0060 (4)

Geometric parameters (Å, º)

N1—N2	1.3640 (12)	C10—H10	0.95
N1—C7	1.3673 (13)	C10—C11	1.3903 (16)
N1—C8	1.4429 (13)	C11—H11	0.95
N2—C1	1.3438 (14)	C11—C12	1.3850 (16)
C1—C2	1.4182 (15)	C12—H12	0.95
C1—C6	1.4243 (14)	C12—C13	1.3923 (15)
C2—H2	0.95	C13—H13	0.95
C2—C3	1.3614 (16)	C14—C15	1.5398 (15)
C3—H3	0.95	C14—C16	1.5391 (15)
C3—C4	1.4258 (16)	C14—C17	1.5395 (15)
C4—H4	0.95	C15—H15A	0.98
C4—C5	1.3667 (16)	C15—H15B	0.98
C5—H5	0.95	C15—H15C	0.98
C5—C6	1.4225 (15)	C16—H16A	0.98
C6—C7	1.4107 (14)	C16—H16B	0.98
C7—C14	1.5258 (14)	C16—H16C	0.98
C8—C9	1.3871 (15)	C17—H17A	0.98
C8—C13	1.3865 (15)	C17—H17B	0.98
C9—H9	0.95	C17—H17C	0.98
C9—C10	1.3854 (16)		
N2—N1—C7	114.81 (8)	C10—C11—H11	119.9
N2—N1—C8	114.32 (8)	C12—C11—C10	120.26 (10)
C7—N1—C8	130.85 (9)	C12—C11—H11	119.9
C1—N2—N1	103.55 (8)	C11—C12—H12	119.9
N2—C1—C2	126.13 (10)	C11—C12—C13	120.26 (10)
N2—C1—C6	111.93 (9)	C13—C12—H12	119.9
C2—C1—C6	121.94 (10)	C8—C13—C12	118.97 (10)
C1—C2—H2	121	C8—C13—H13	120.5
C3—C2—C1	117.91 (10)	C12—C13—H13	120.5
C3—C2—H2	121	C7—C14—C15	109.43 (8)
C2—C3—H3	119.5	C7—C14—C16	112.28 (9)

C2—C3—C4	120.99 (10)	C7—C14—C17	109.83 (9)
C4—C3—H3	119.5	C16—C14—C15	110.01 (9)
C3—C4—H4	119	C16—C14—C17	107.08 (9)
C5—C4—C3	121.92 (10)	C17—C14—C15	108.10 (9)
C5—C4—H4	119	C14—C15—H15A	109.5
C4—C5—H5	120.5	C14—C15—H15B	109.5
C4—C5—C6	118.93 (10)	C14—C15—H15C	109.5
C6—C5—H5	120.5	H15A—C15—H15B	109.5
C5—C6—C1	118.30 (9)	H15A—C15—H15C	109.5
C7—C6—C1	105.29 (9)	H15B—C15—H15C	109.5
C7—C6—C5	136.41 (10)	C14—C16—H16A	109.5
N1—C7—C6	104.42 (9)	C14—C16—H16B	109.5
N1—C7—C14	124.75 (9)	C14—C16—H16C	109.5
C6—C7—C14	130.83 (9)	H16A—C16—H16B	109.5
C9—C8—N1	118.19 (9)	H16A—C16—H16C	109.5
C13—C8—N1	120.66 (9)	H16B—C16—H16C	109.5
C13—C8—C9	121.06 (10)	C14—C17—H17A	109.5
C8—C9—H9	120.2	C14—C17—H17B	109.5
C10—C9—C8	119.60 (10)	C14—C17—H17C	109.5
C10—C9—H9	120.2	H17A—C17—H17B	109.5
C9—C10—H10	120.1	H17A—C17—H17C	109.5
C9—C10—C11	119.81 (10)	H17B—C17—H17C	109.5
C11—C10—H10	120.1		

N1—N2—C1—C2	179.17 (10)	C4—C5—C6—C1	−0.23 (15)
N1—N2—C1—C6	−0.30 (11)	C4—C5—C6—C7	179.83 (11)
N1—C7—C14—C15	82.17 (12)	C5—C6—C7—N1	−179.47 (11)
N1—C7—C14—C16	−40.29 (14)	C5—C6—C7—C14	−0.4 (2)
N1—C7—C14—C17	−159.31 (10)	C6—C1—C2—C3	0.00 (15)
N1—C8—C9—C10	179.16 (10)	C6—C7—C14—C15	−96.74 (13)
N1—C8—C13—C12	−178.81 (9)	C6—C7—C14—C16	140.81 (11)
N2—N1—C7—C6	−0.85 (11)	C6—C7—C14—C17	21.79 (15)
N2—N1—C7—C14	−179.99 (9)	C7—N1—N2—C1	0.73 (11)
N2—N1—C8—C9	−73.31 (12)	C7—N1—C8—C9	104.55 (13)
N2—N1—C8—C13	103.23 (11)	C7—N1—C8—C13	−78.90 (14)
N2—C1—C2—C3	−179.43 (10)	C8—N1—N2—C1	178.95 (8)
N2—C1—C6—C5	179.86 (9)	C8—N1—C7—C6	−178.71 (10)
N2—C1—C6—C7	−0.19 (12)	C8—N1—C7—C14	2.15 (16)
C1—C2—C3—C4	−0.48 (16)	C8—C9—C10—C11	−1.02 (18)
C1—C6—C7—N1	0.59 (11)	C9—C8—C13—C12	−2.37 (16)
C1—C6—C7—C14	179.66 (10)	C9—C10—C11—C12	−0.81 (18)
C2—C1—C6—C5	0.36 (15)	C10—C11—C12—C13	1.06 (17)
C2—C1—C6—C7	−179.69 (9)	C11—C12—C13—C8	0.51 (16)
C2—C3—C4—C5	0.62 (17)	C13—C8—C9—C10	2.63 (17)
C3—C4—C5—C6	−0.24 (16)		

11. NMR spectra

1,3-Dioxoisoindolin-2-yl pivalate (1a)

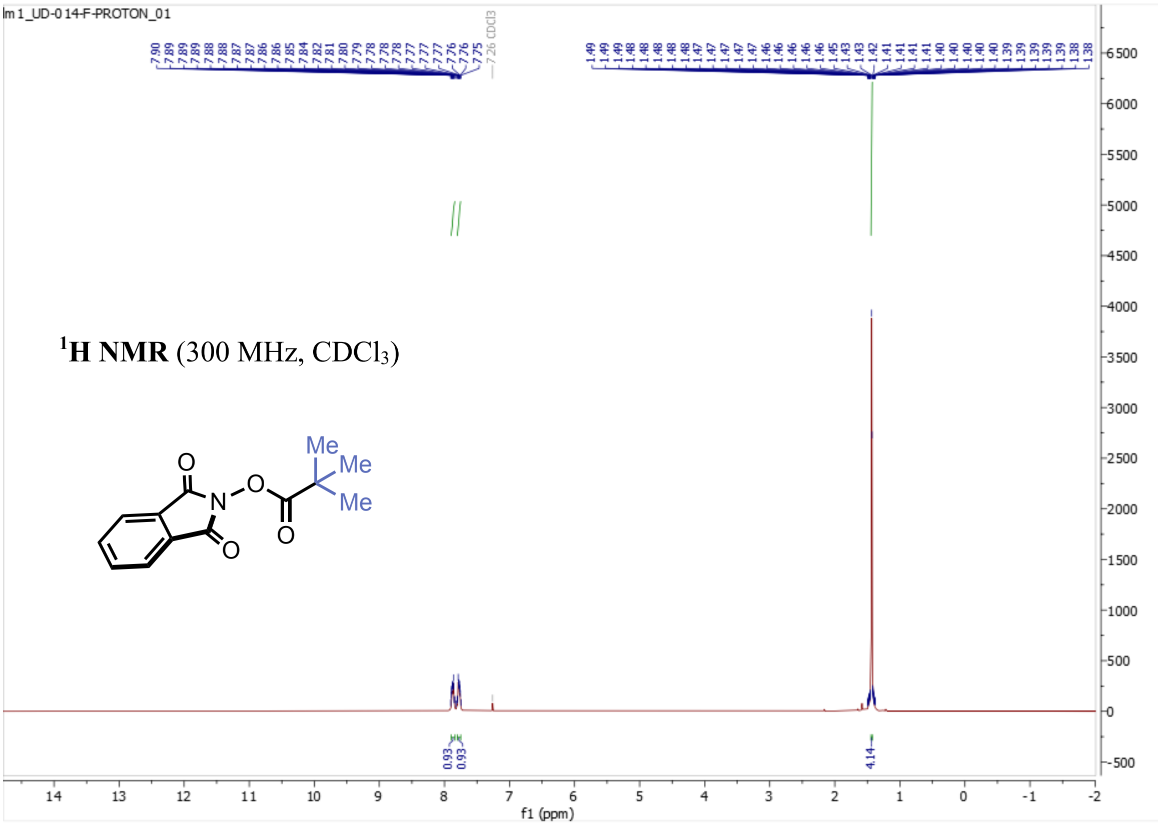

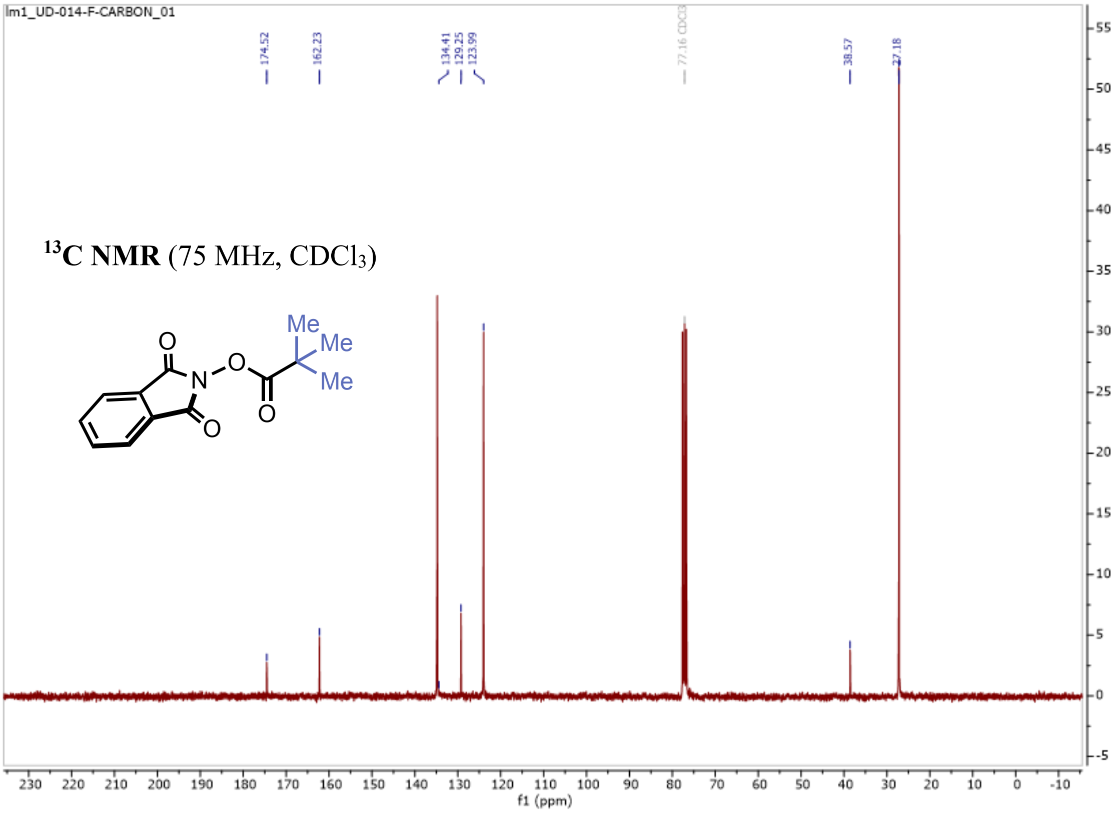

1,3-Dioxoisoindolin-2-yl adamantane-1-carboxylate (1b)

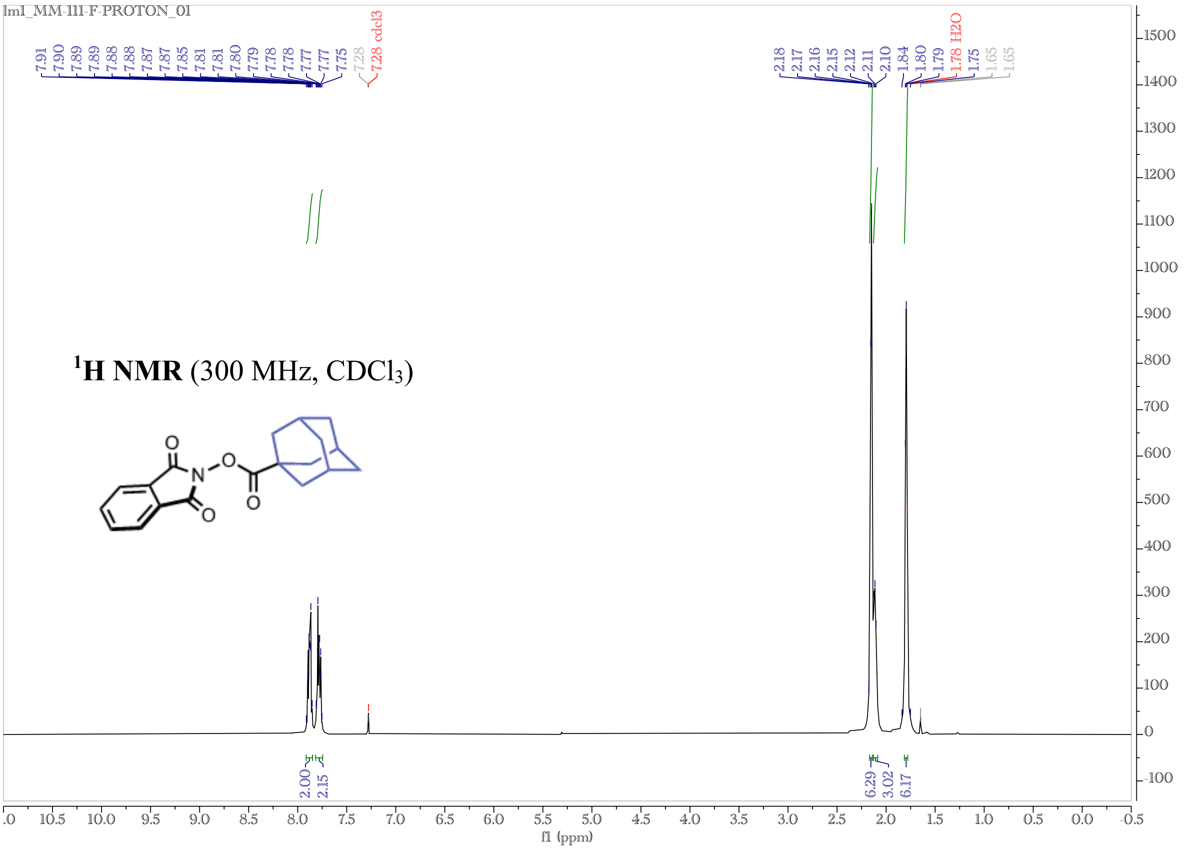

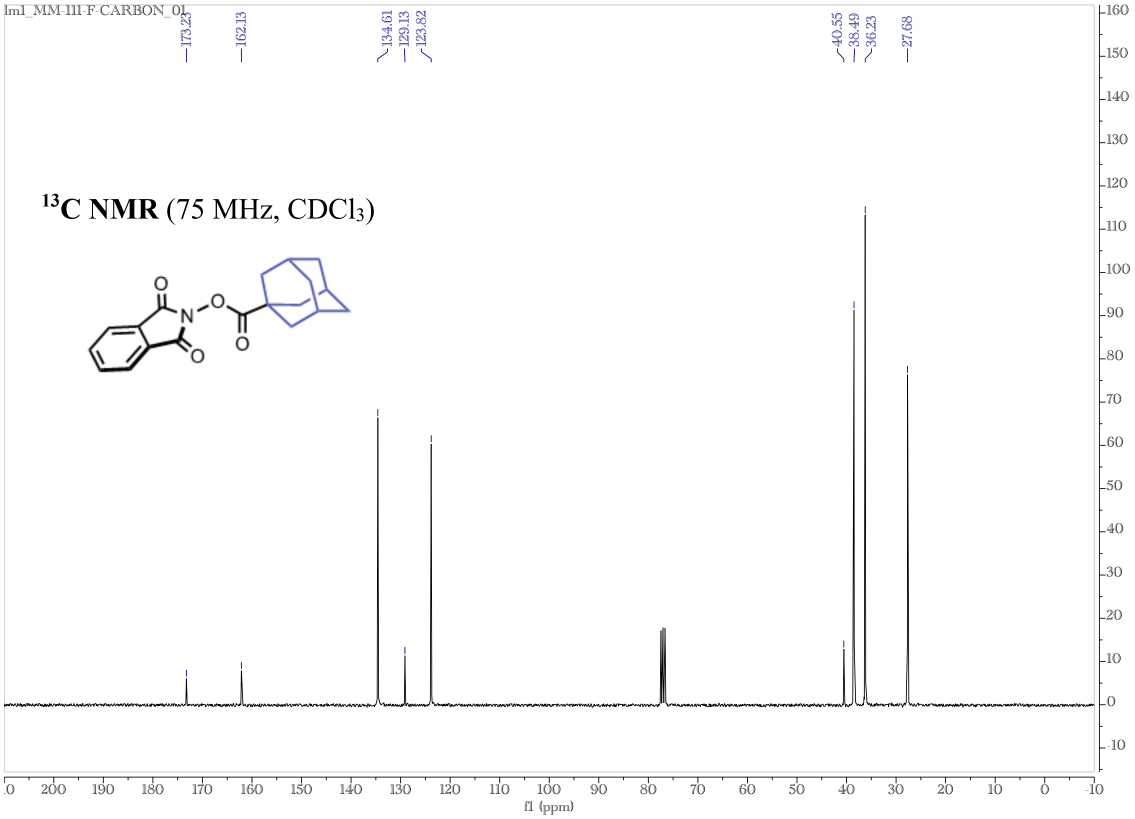

1,3-Dioxoisoindolin-2-yl 5-(2,5-dimethylphenoxy)-2,2-dimethylpentanoate (1c)

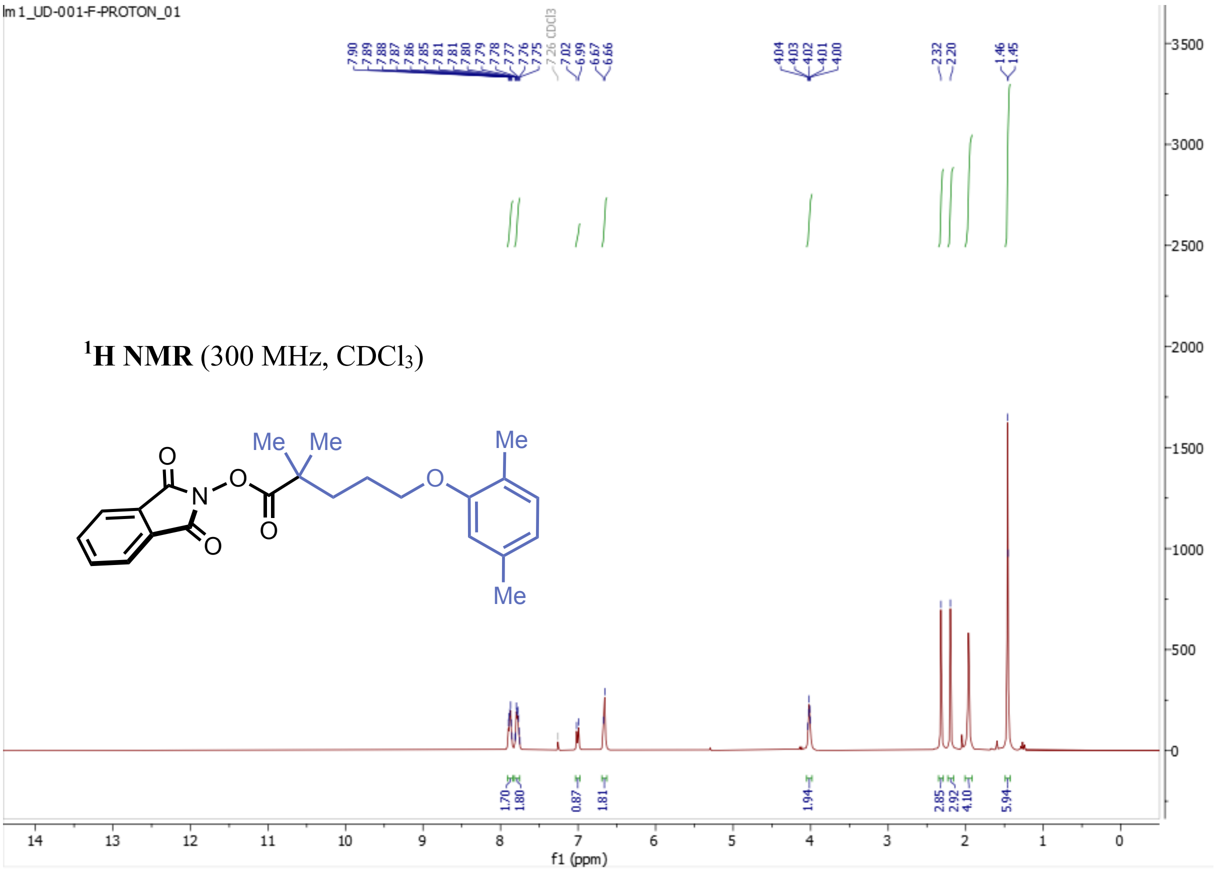

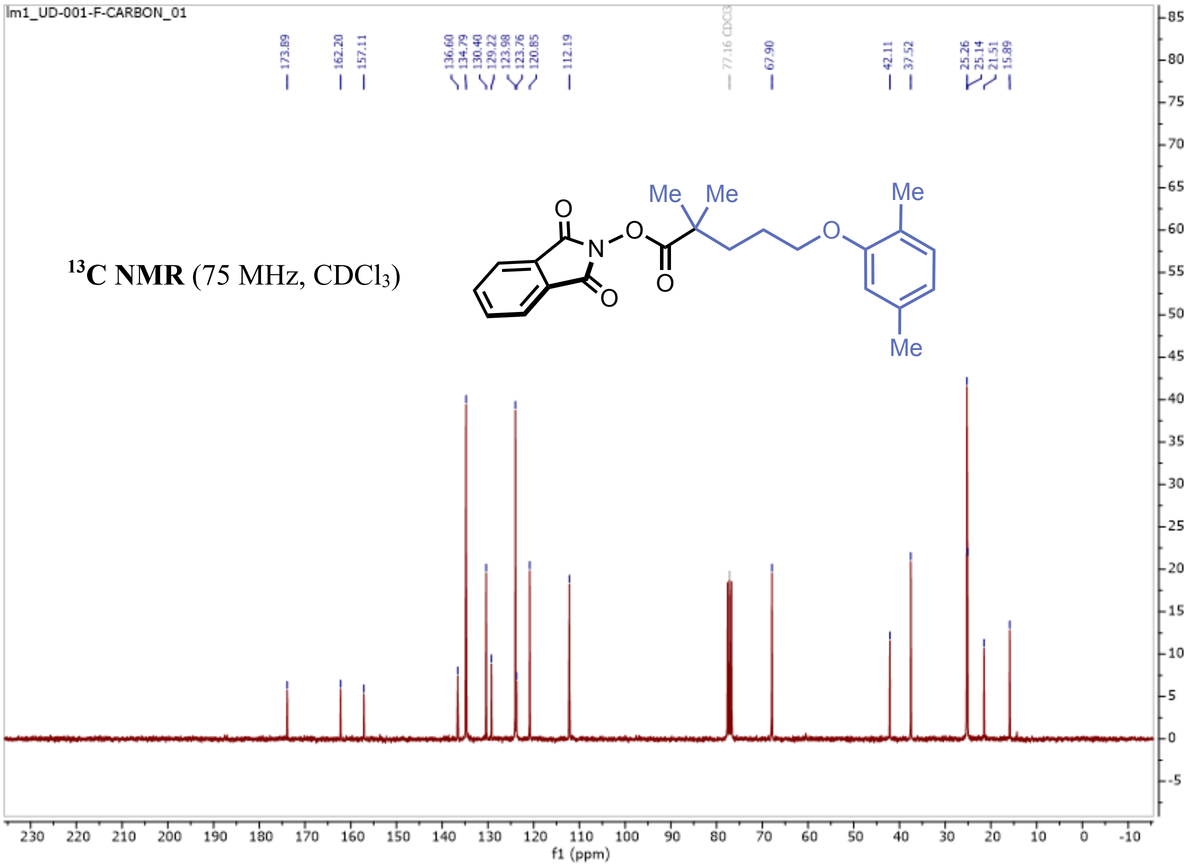

1,3-Dioxoisoindolin-2-yl 1-methylcyclohexane-1-carboxylate (1d)

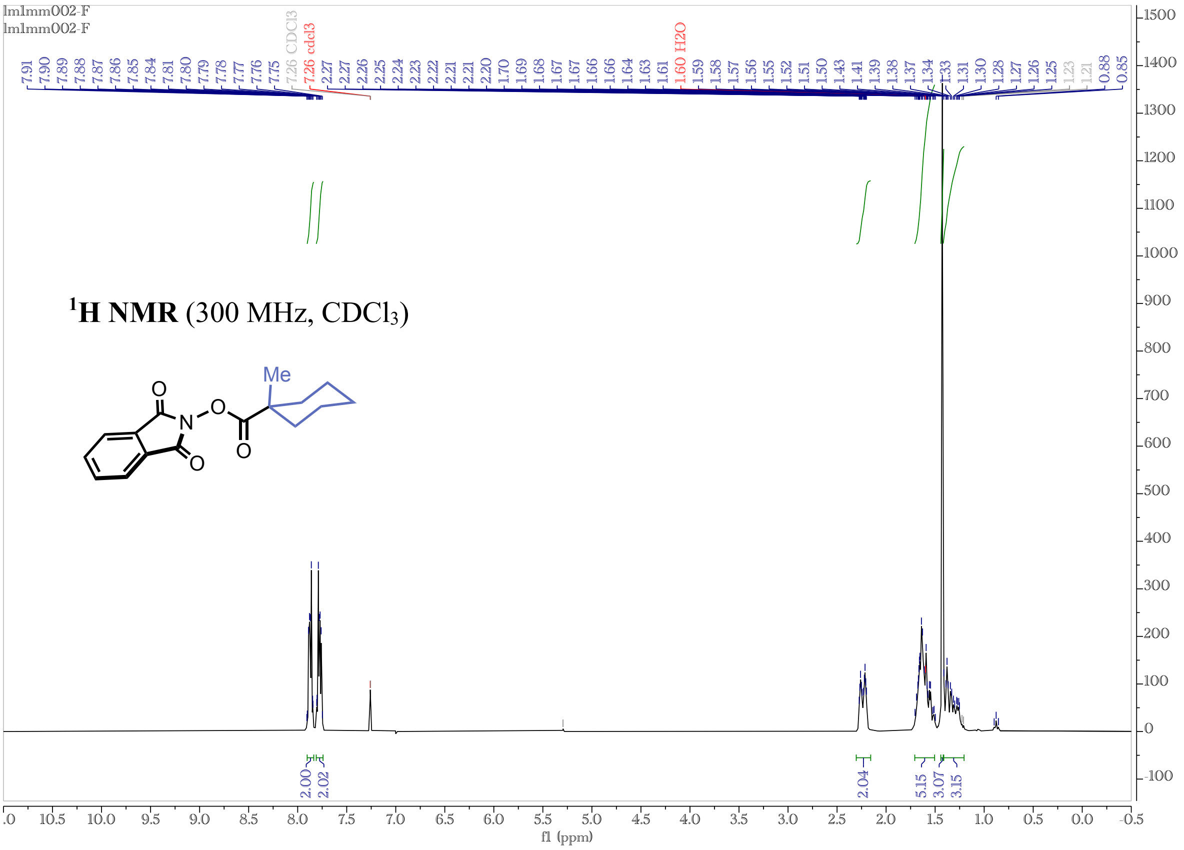

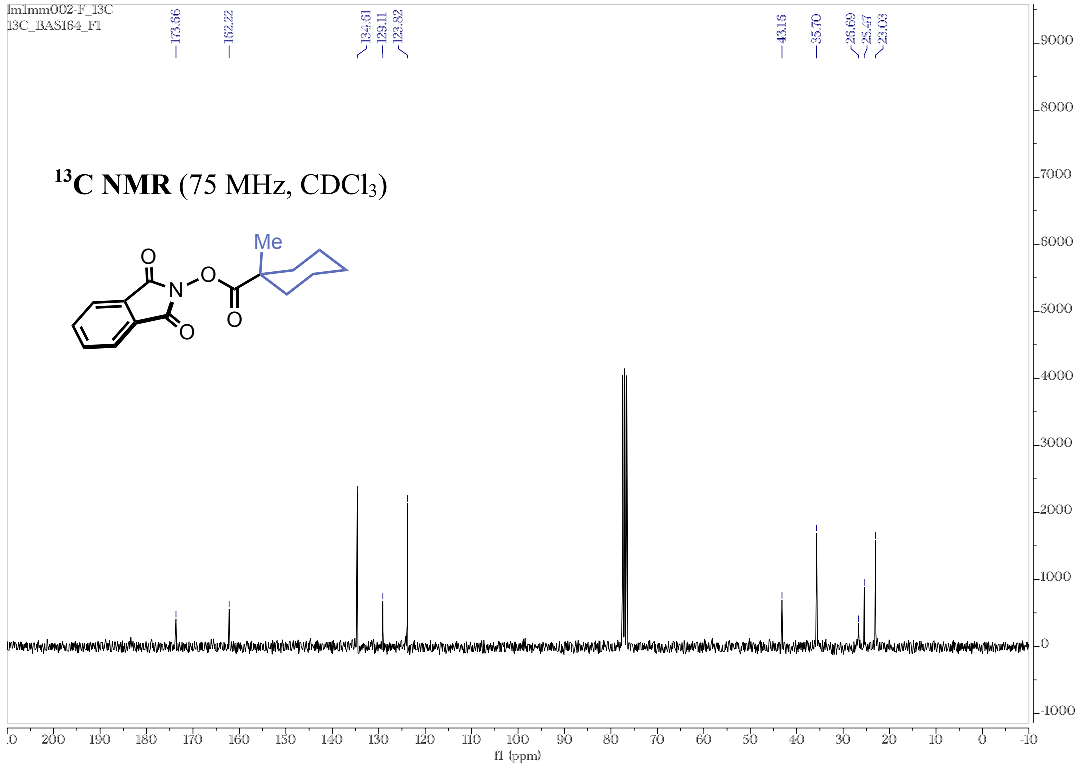

1-(*tert*-Butyl) 2-(1,3-dioxoisoindolin-2-yl) pyrrolidine-1,2-dicarboxylate (1e) – some signals are broad and split due to the presence of diastereomeric rotamers.

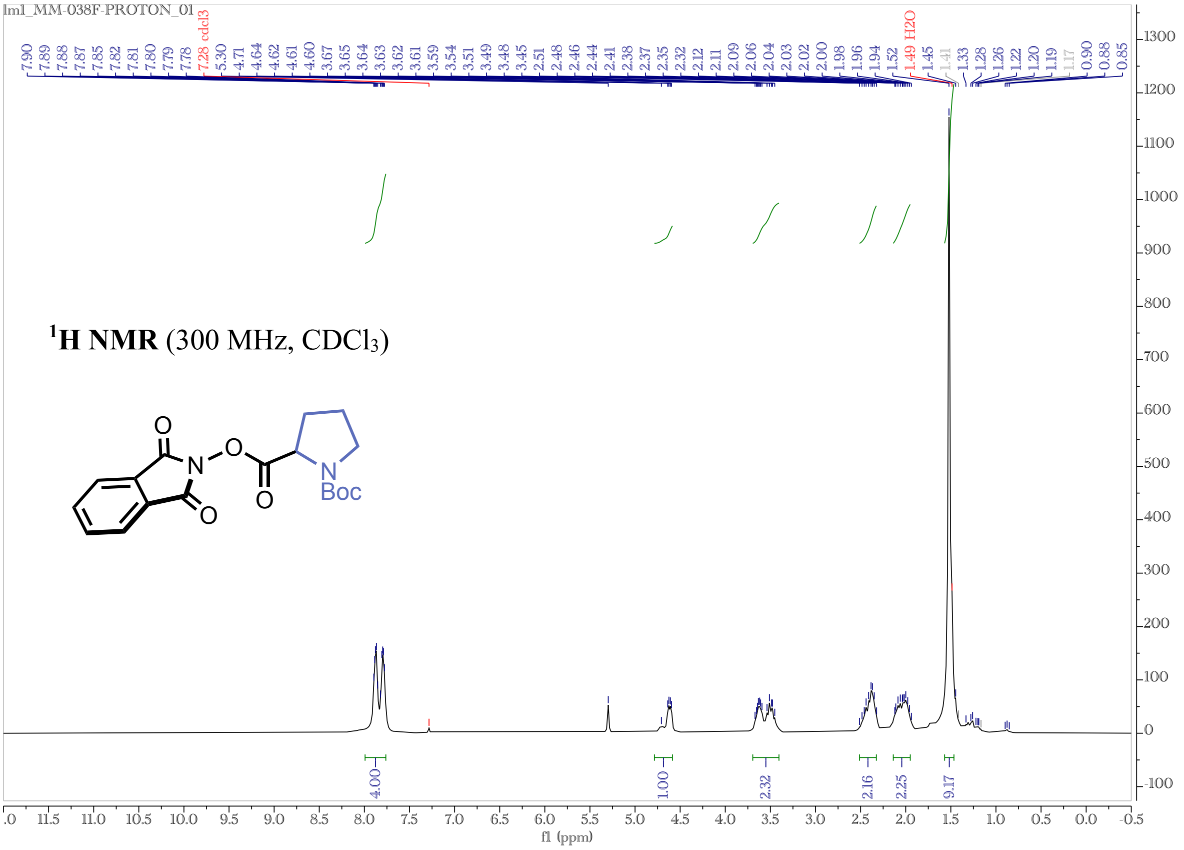

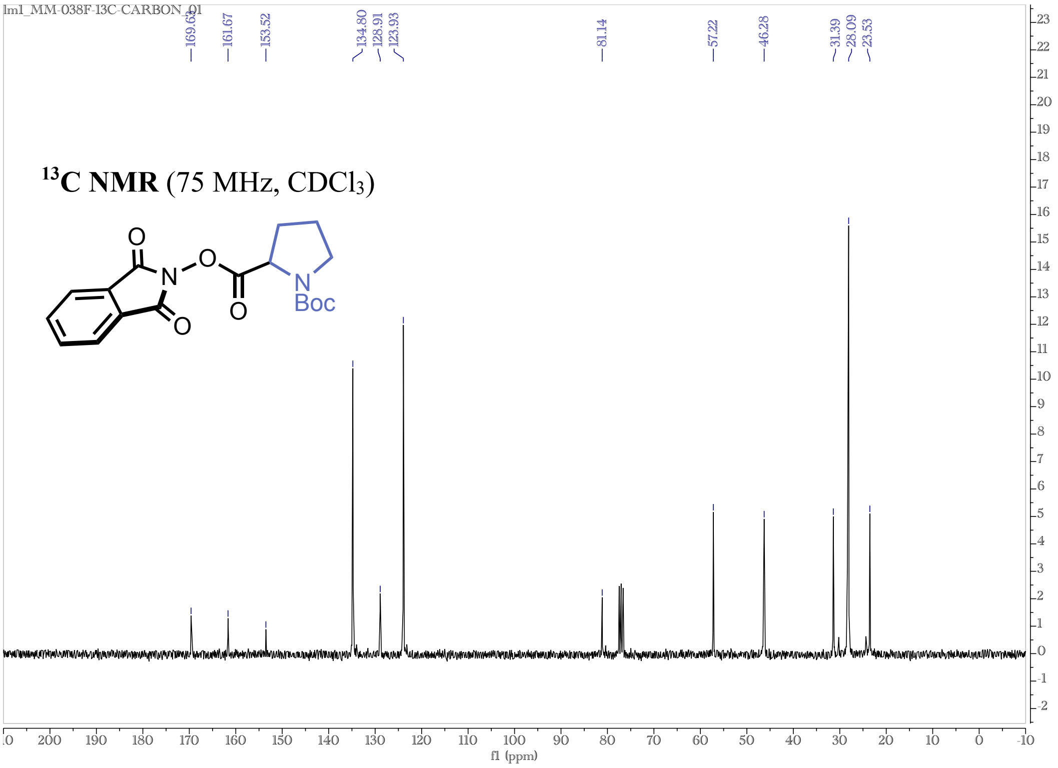

1,3-Dioxoisoindolin-2-yl cyclohexanecarboxylate (1f)

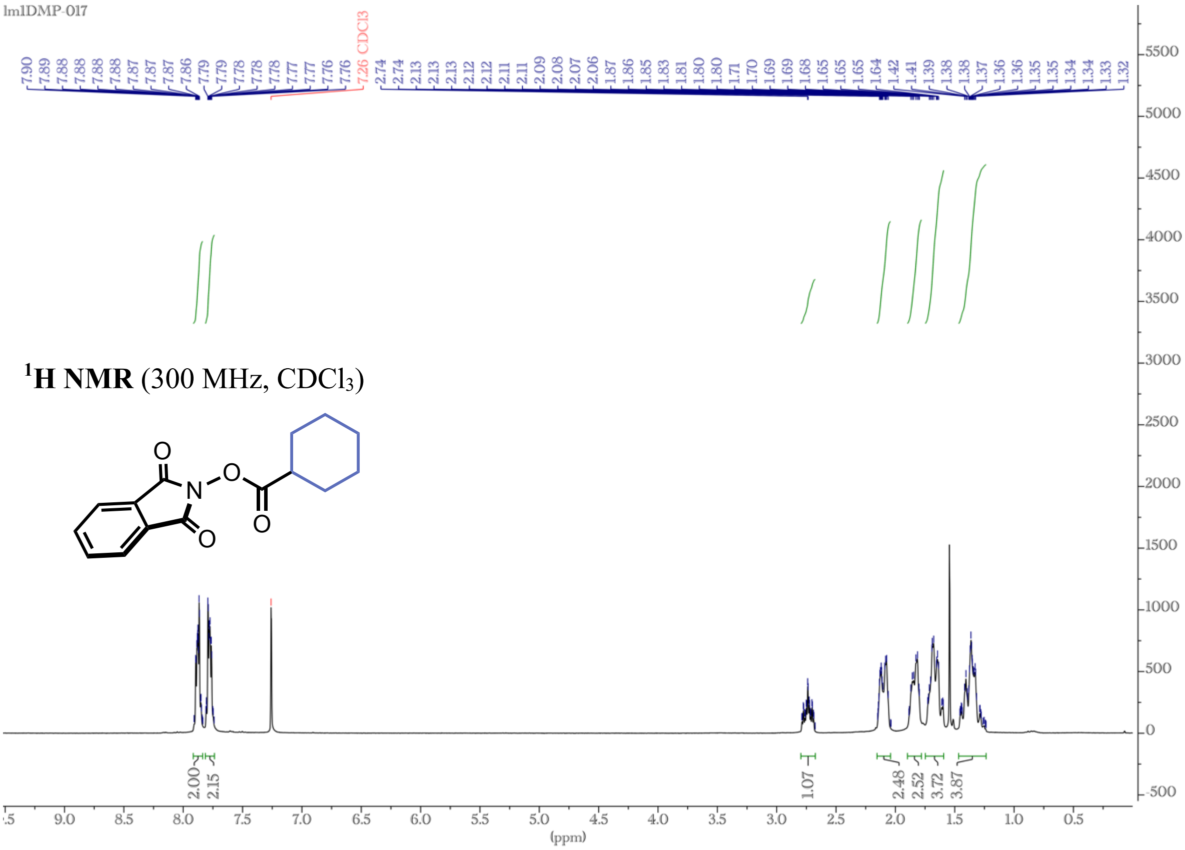

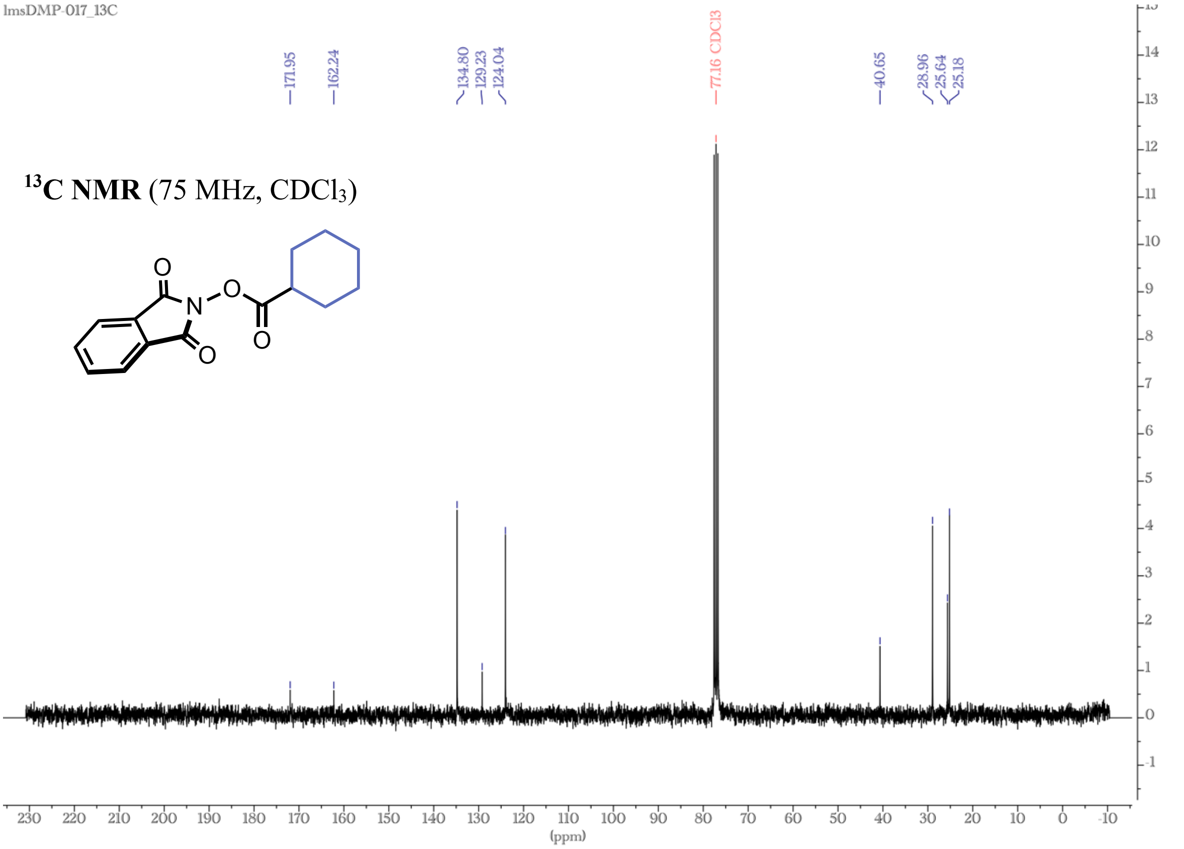

1,3-Dioxoisoindolin-2-yl 6-oxo-6-phenylhexanoate (1g)

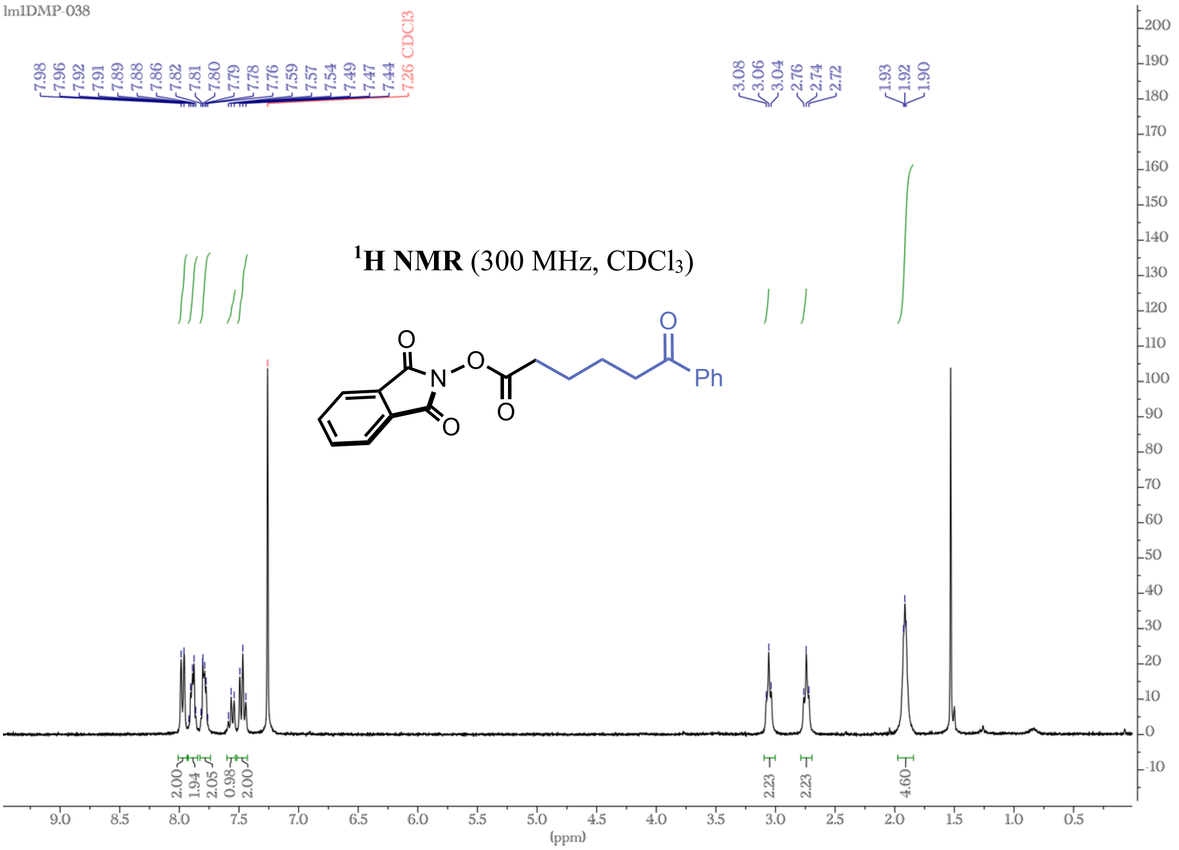

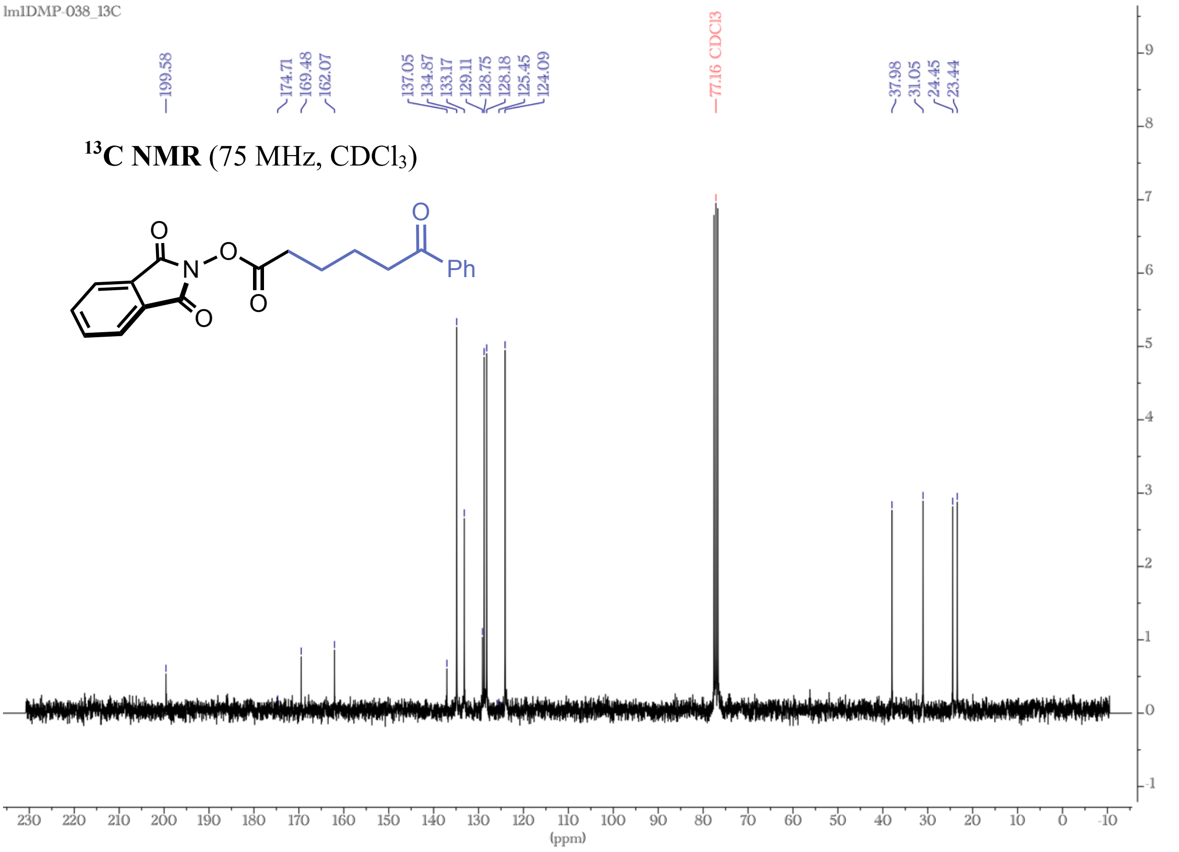

1,3-Dioxoisoindolin-2-yl 3-(3,4-dimethoxyphenyl) propanoate (1h)

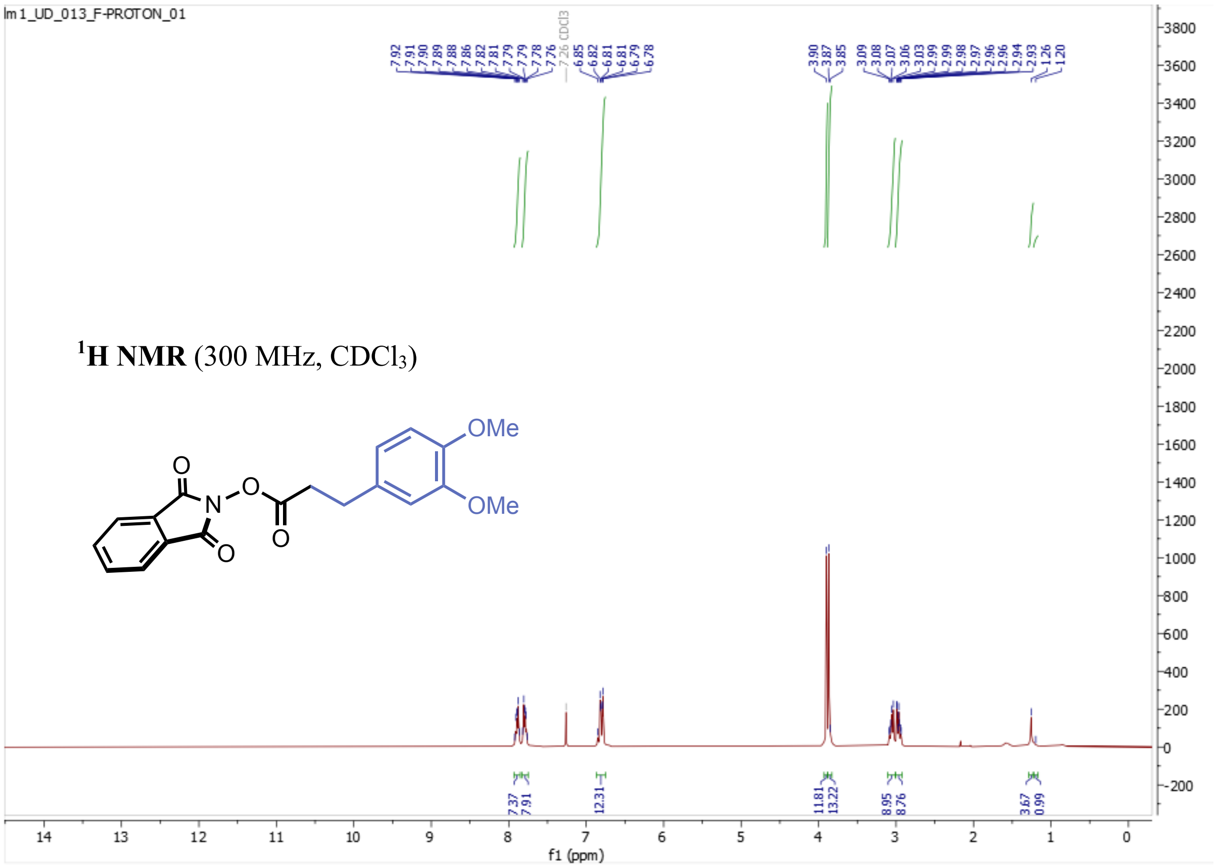

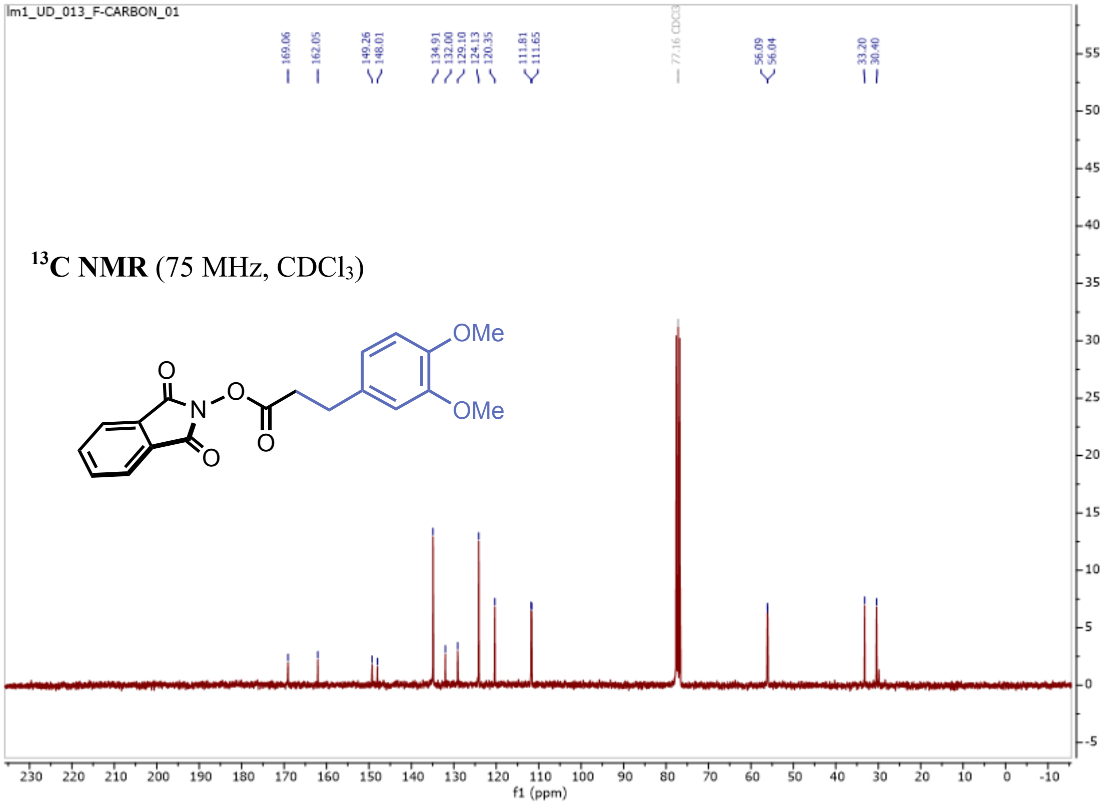

2,4-Dibenzyl-1,2,4-triazine-3,5(2*H*,4*H*)-dione (2a)

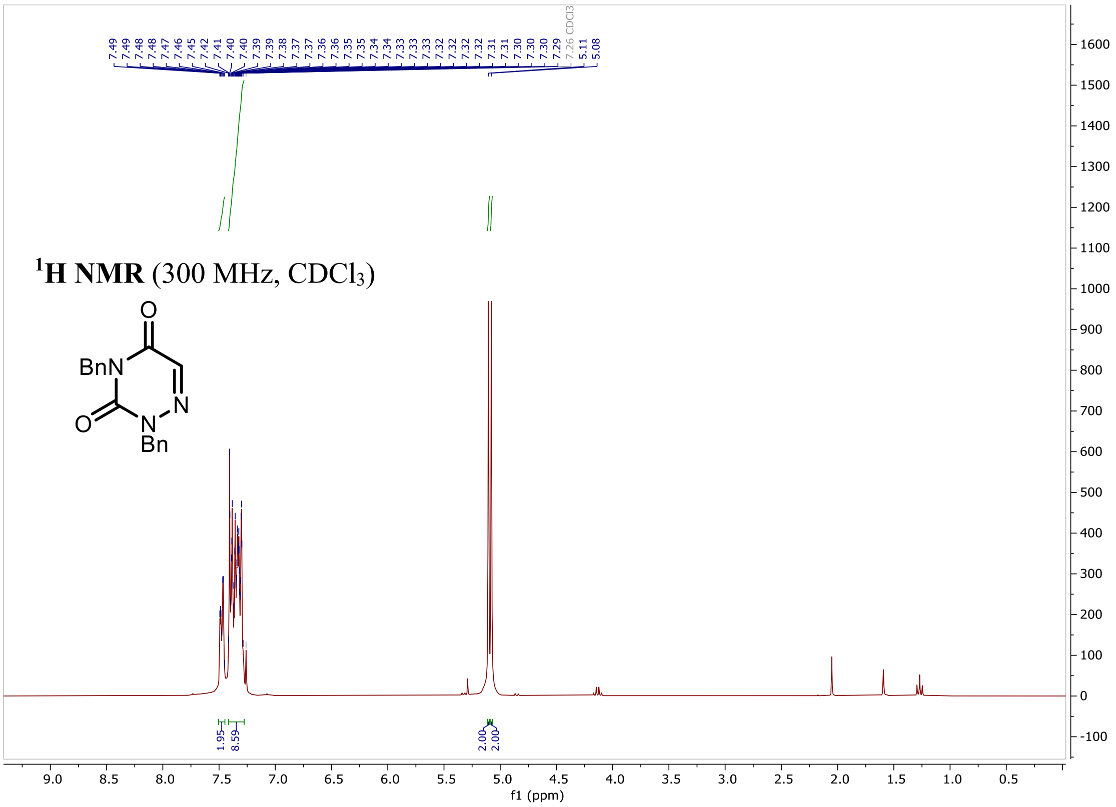

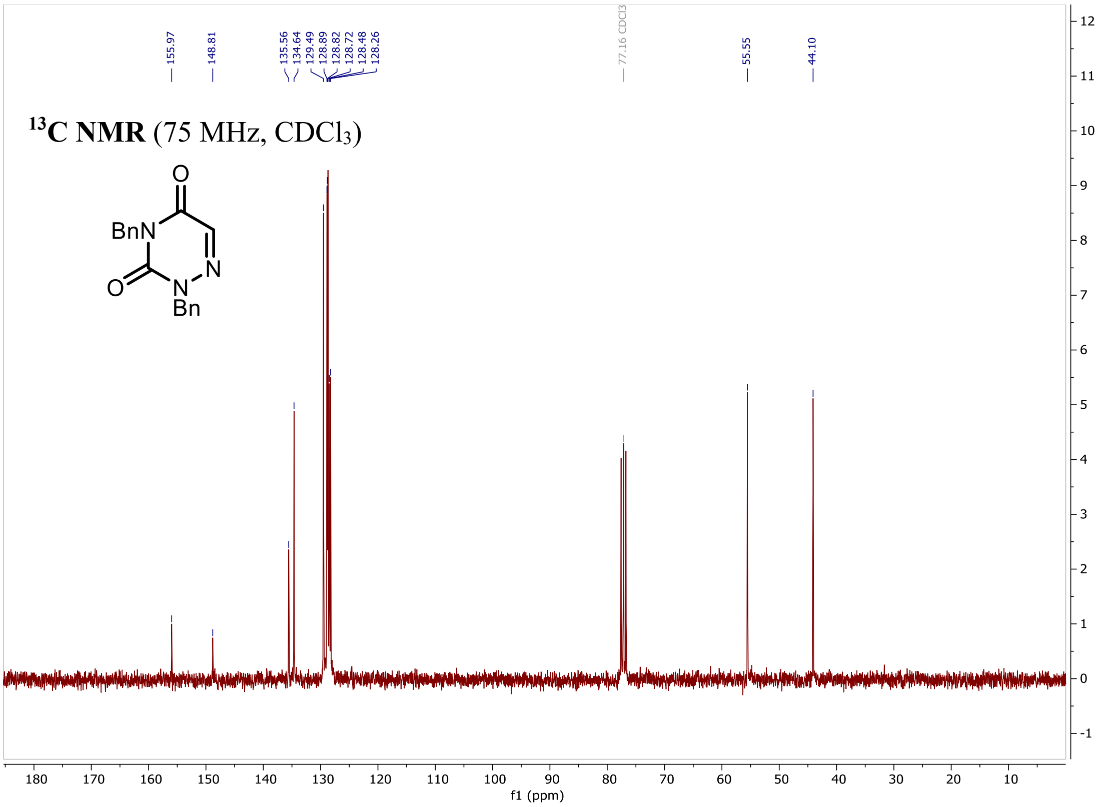

2,4-Dimethyl-1,2,4-triazine-3,5(2*H*,4*H*)-dione (2b)

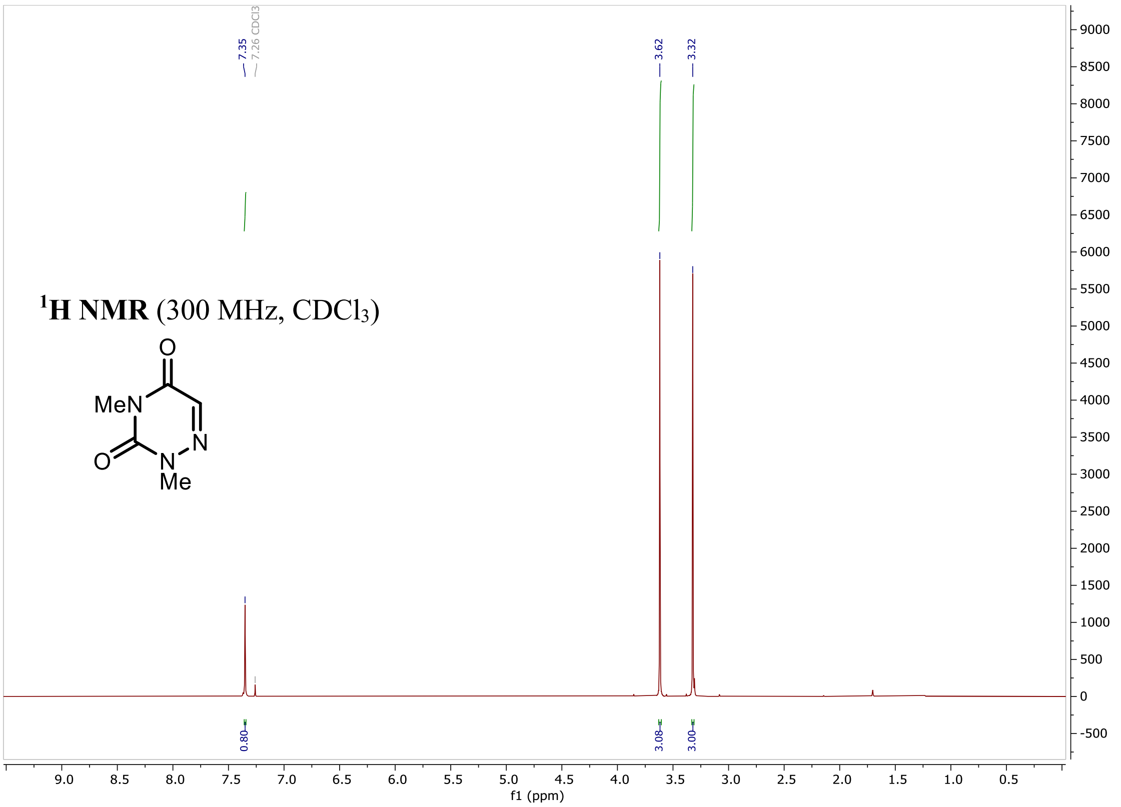

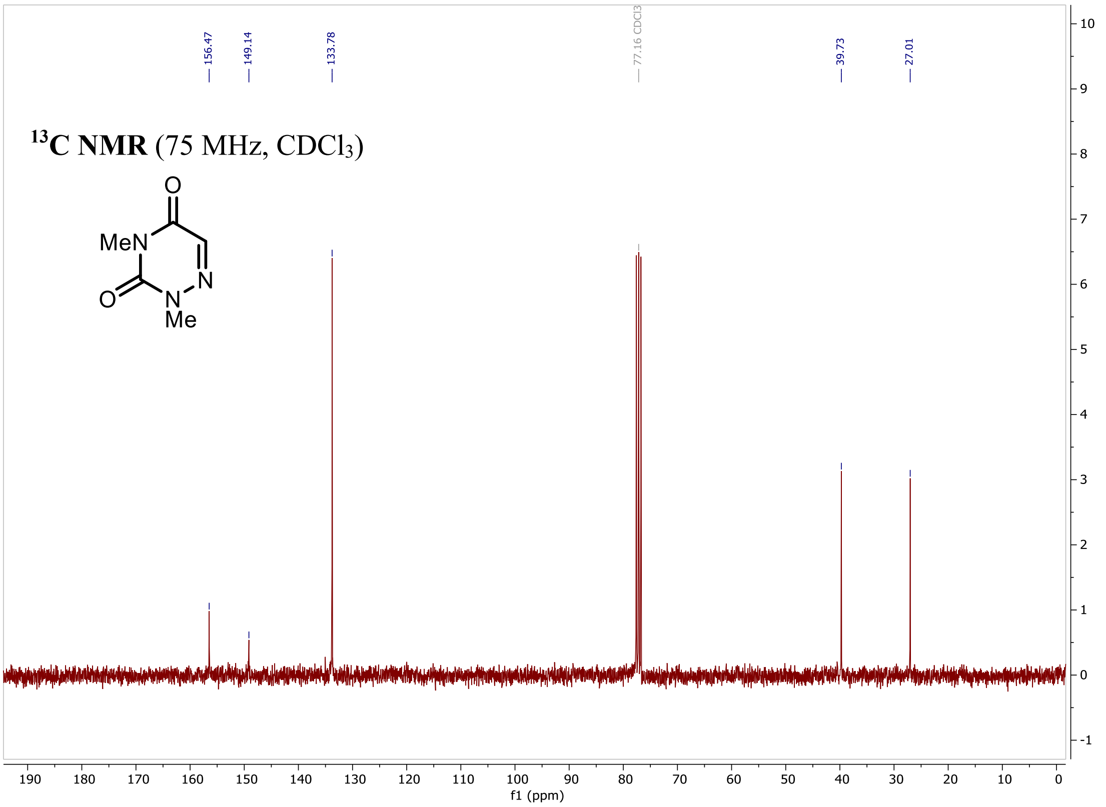

2-Benzyl-1,2,4-triazine-3,5(2*H*,4*H*)-dione (2c)

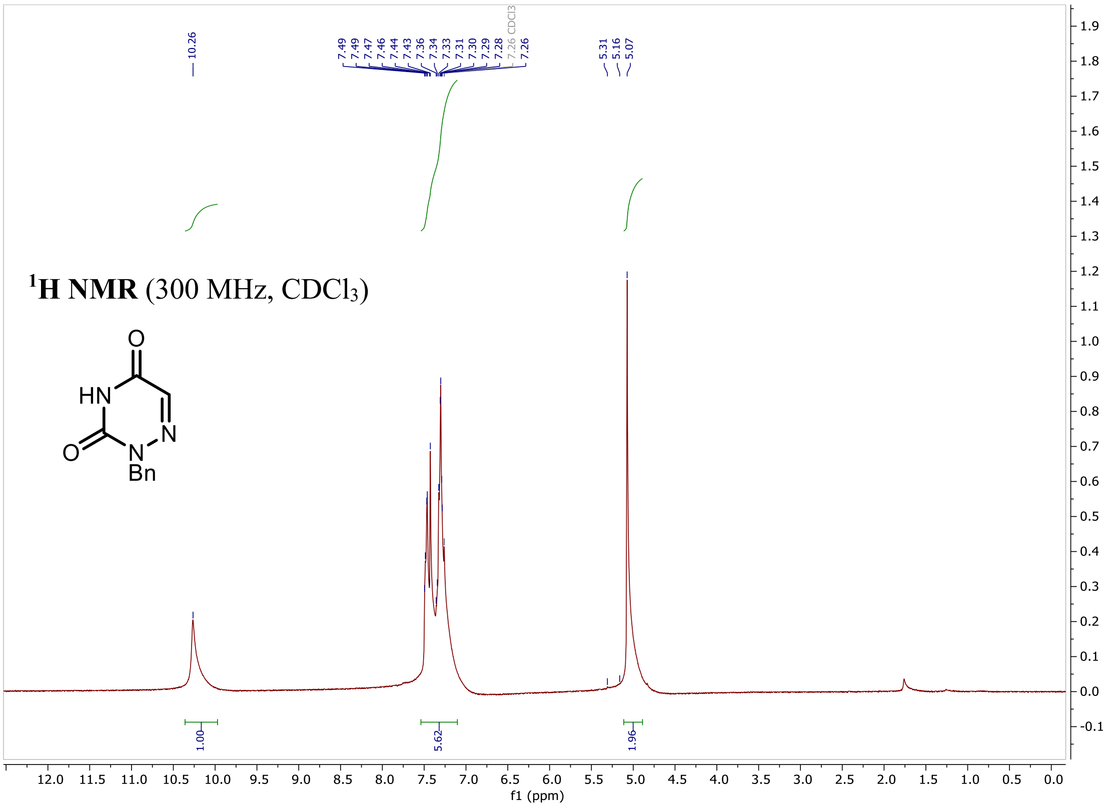

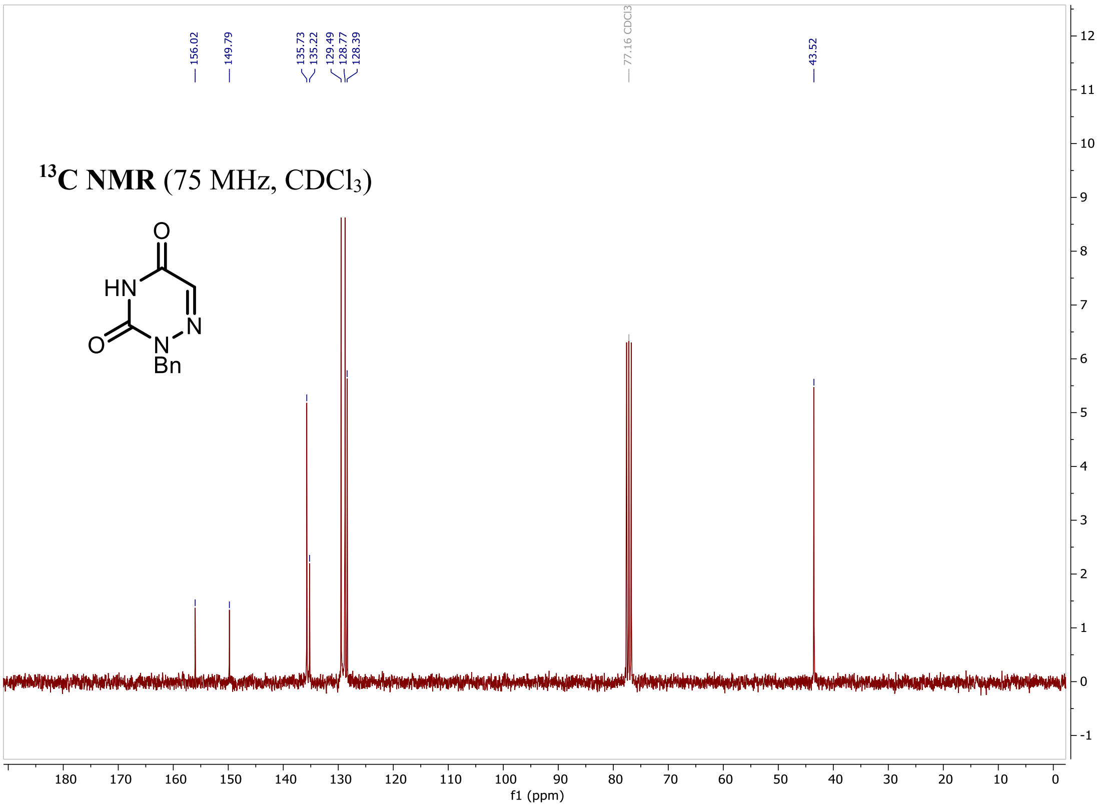

Ethyl 2-(2-benzyl-3,5-dioxo-2,5-dihydro-1,2,4-triazin-4(3*H*)-yl) acetate (2d)

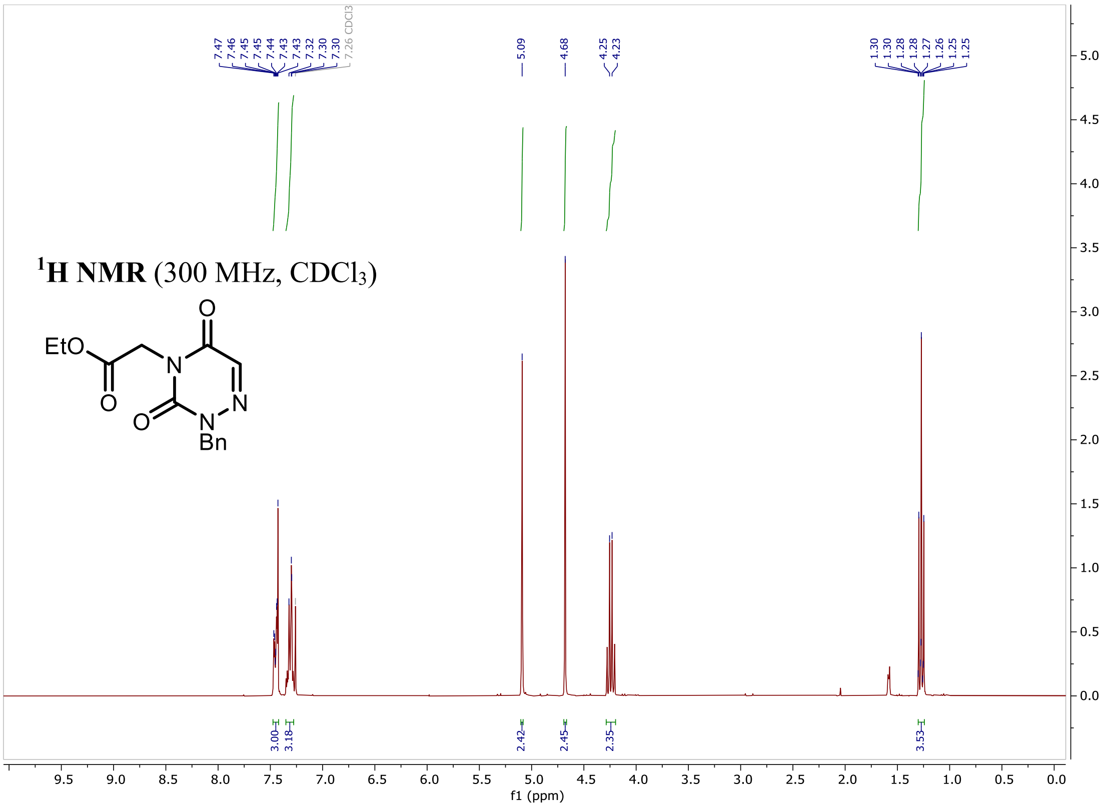

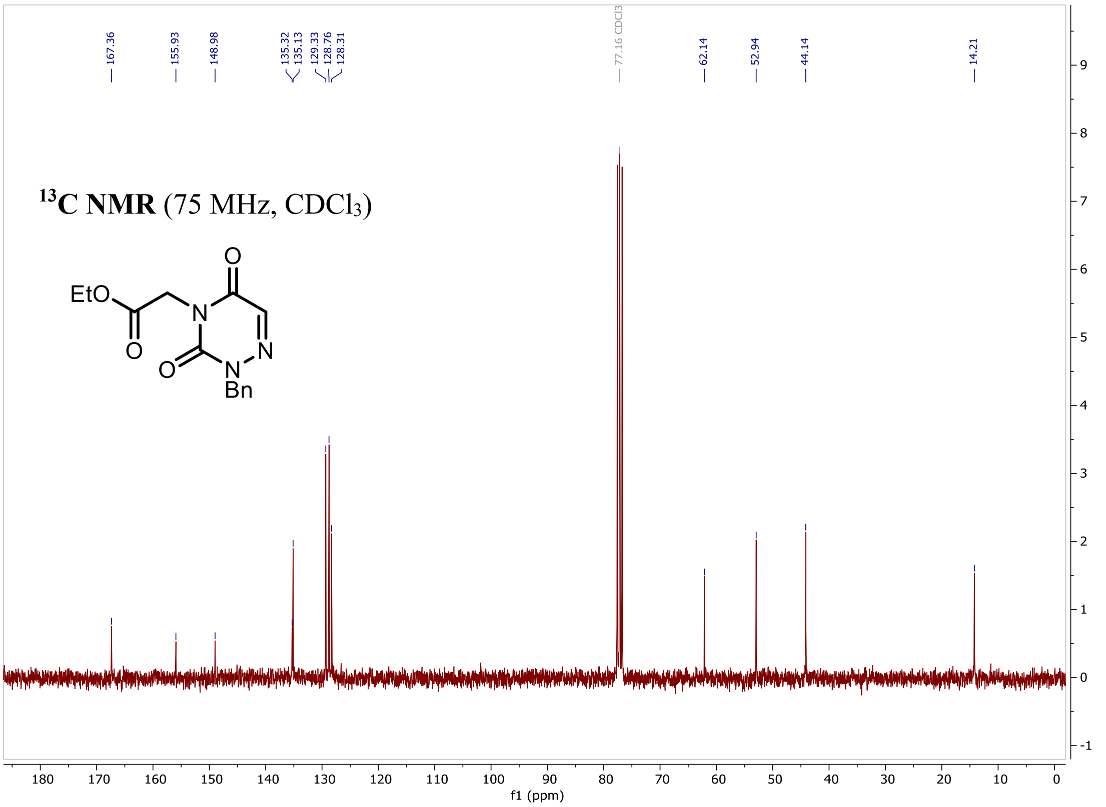

2-Benzyl-4-(prop-2-yn-1-yl)-1,2,4-triazine-3,5(2*H*,4*H*)-dione (2e)

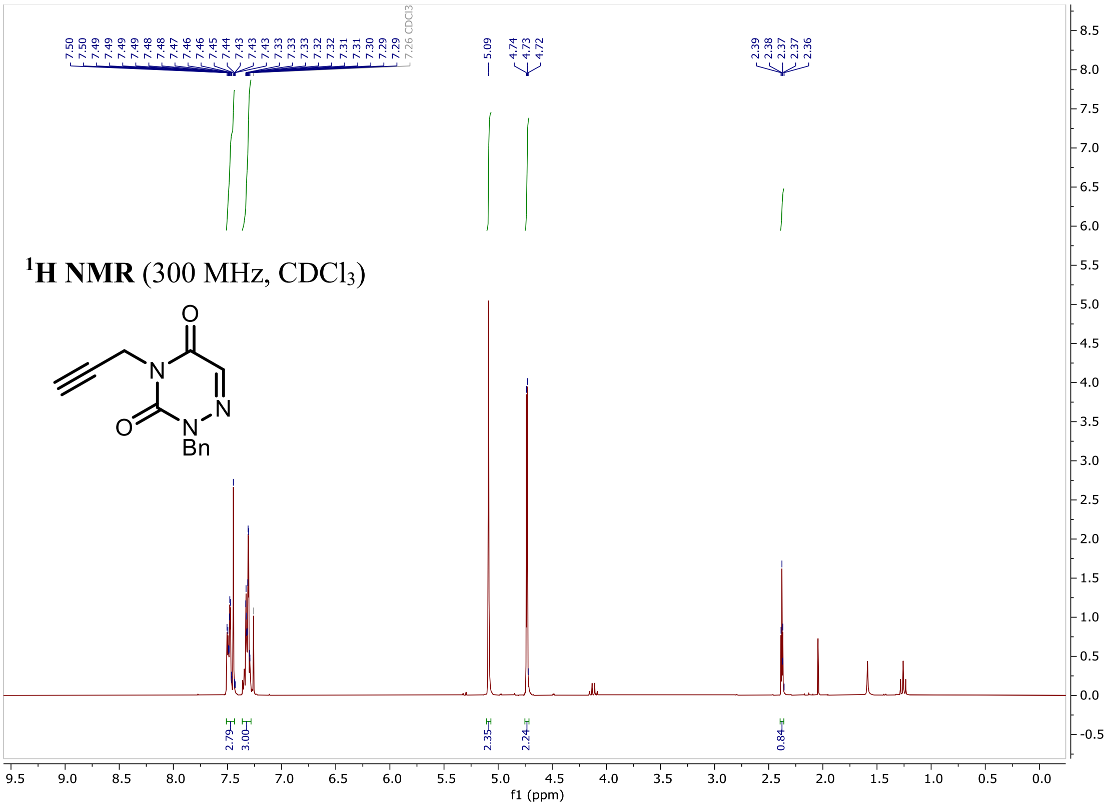

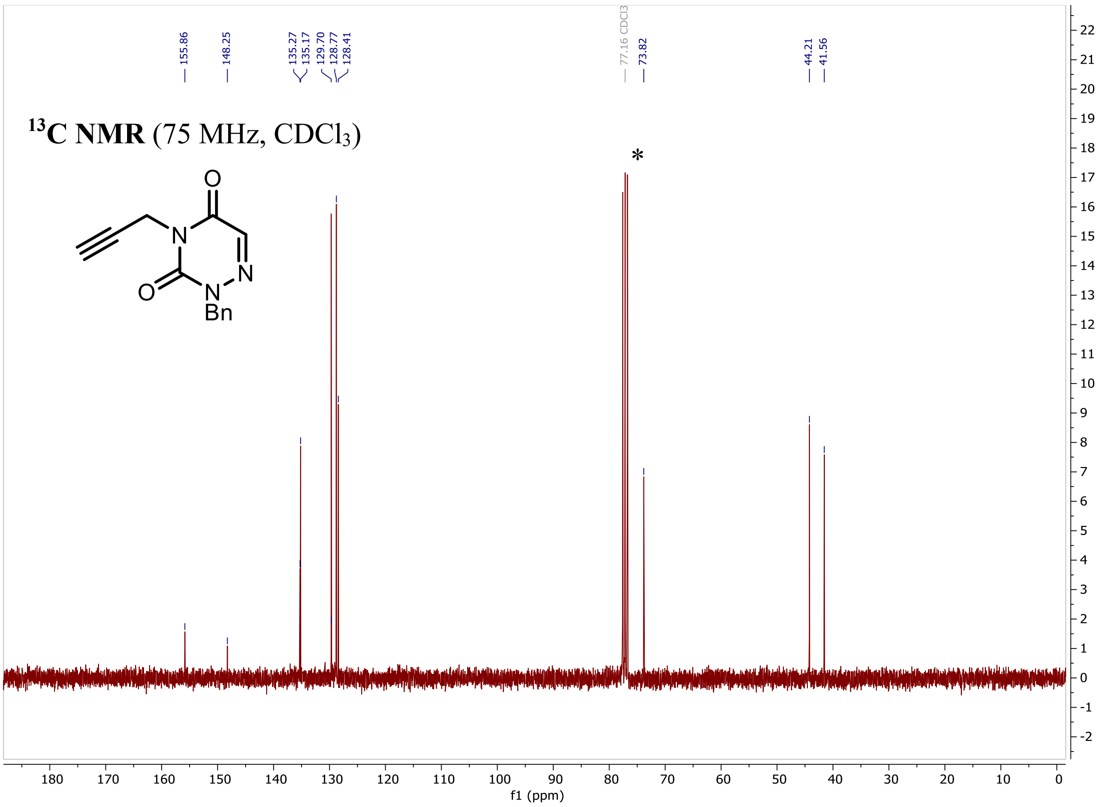

* The signal at δ 77.26 ppm is overlapped with the solvent peak.

1-Benzylquinoxalin-2(1*H*)-one (2f)

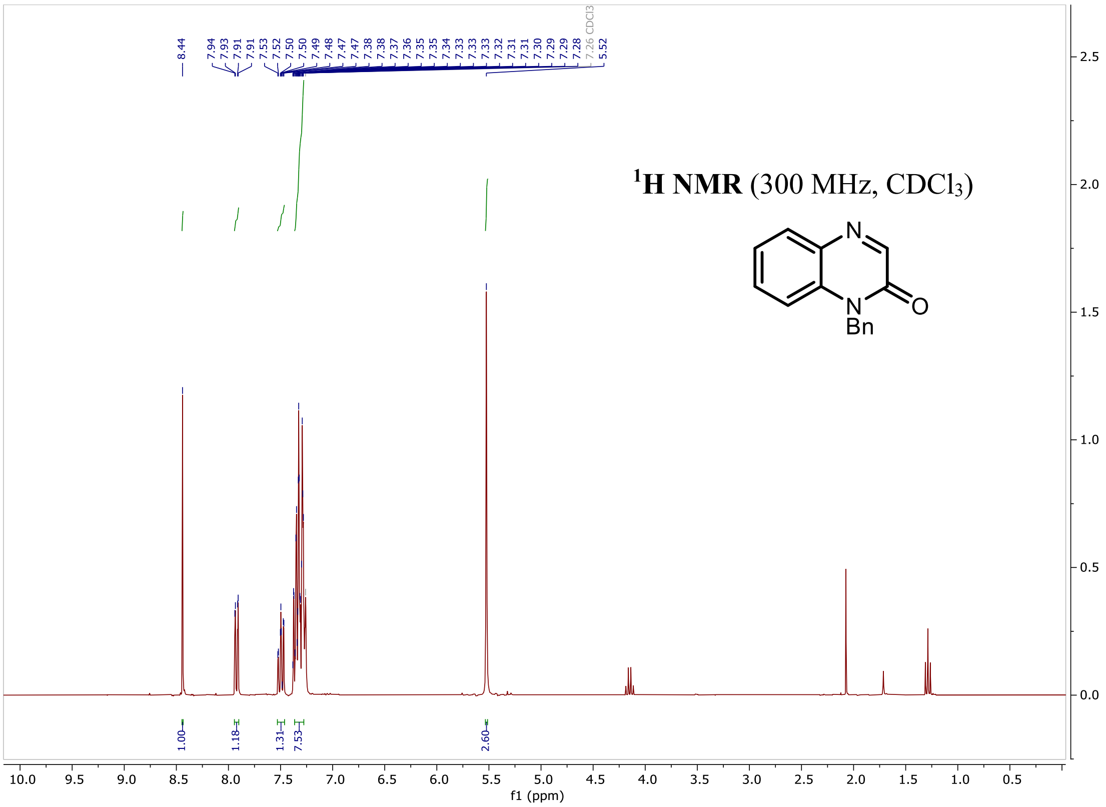

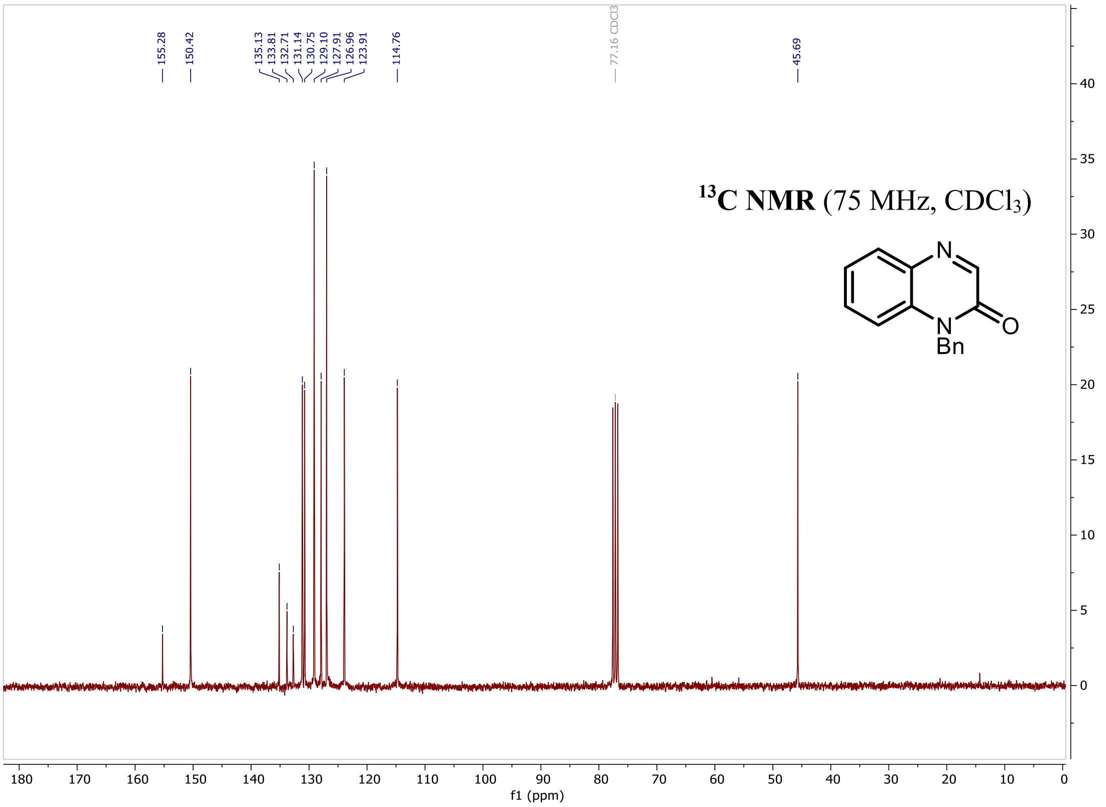

2,4-Dibenzyl-6-(*tert*-butyl)-1,2,4-triazine-3,5(2*H*,4*H*)-dione (3a)

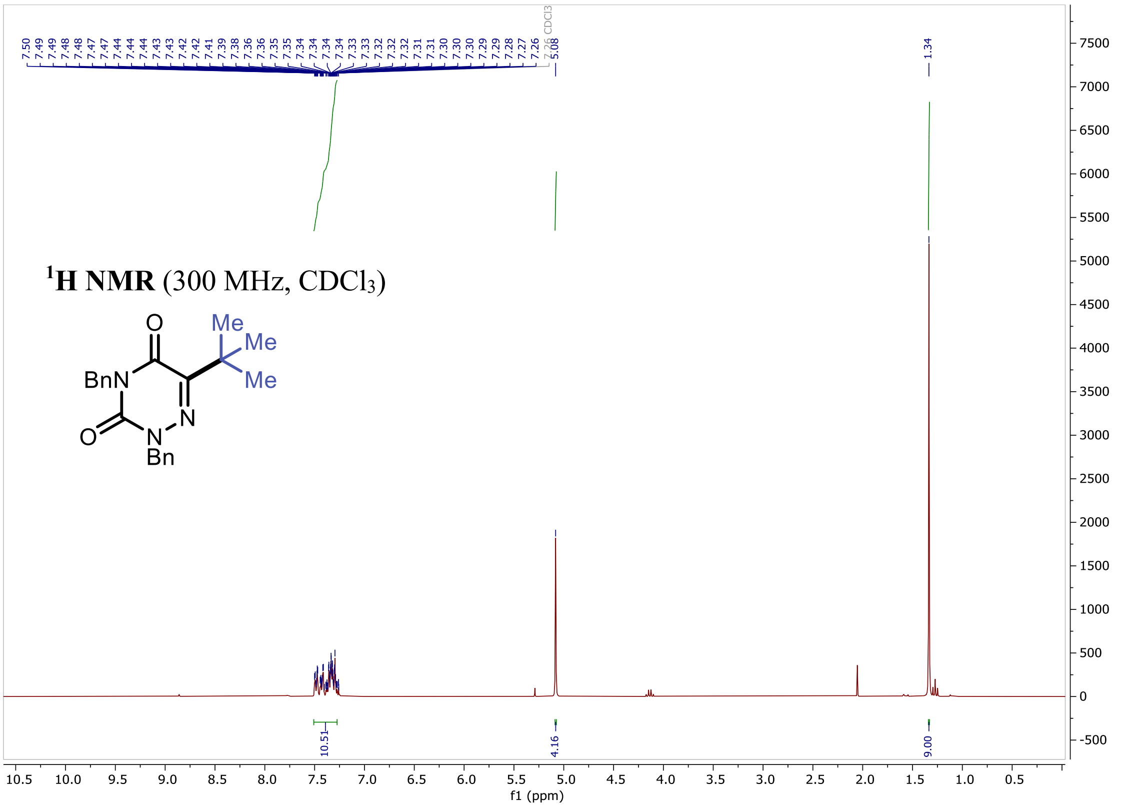

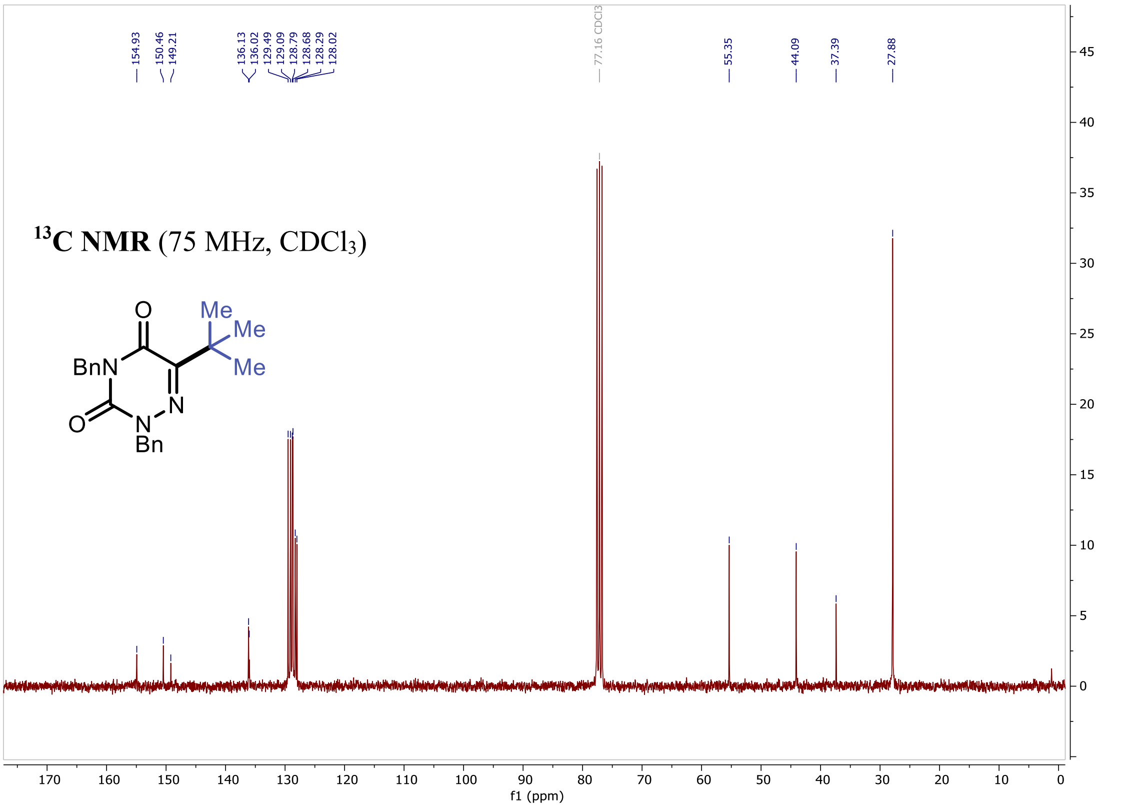

6-(Adamantan-1-yl)-2,4-dibenzyl-1,2,4-triazine-3,5(2*H*,4*H*)-dione (3b)

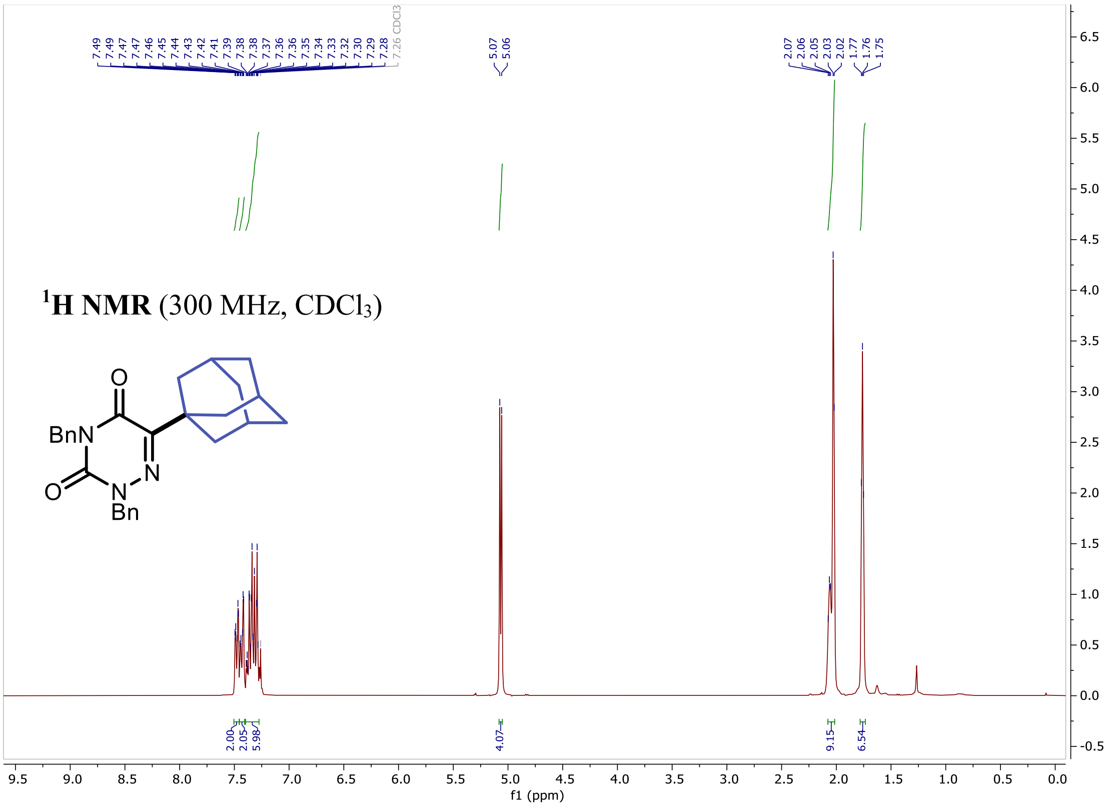

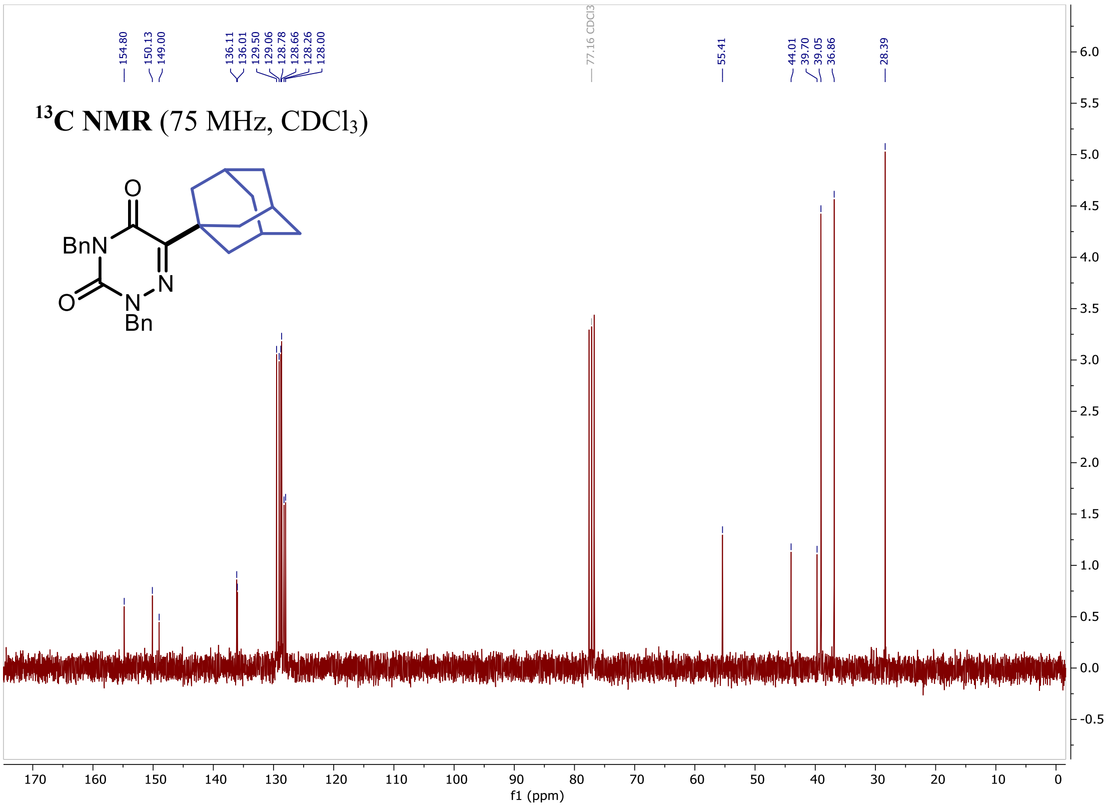

2,4-Dibenzyl-6-(5-(2,5-dimethylphenoxy)-2-methylpentan-2-yl)-1,2,4-triazine-3,5(2*H*,4*H*)-dione (3c)

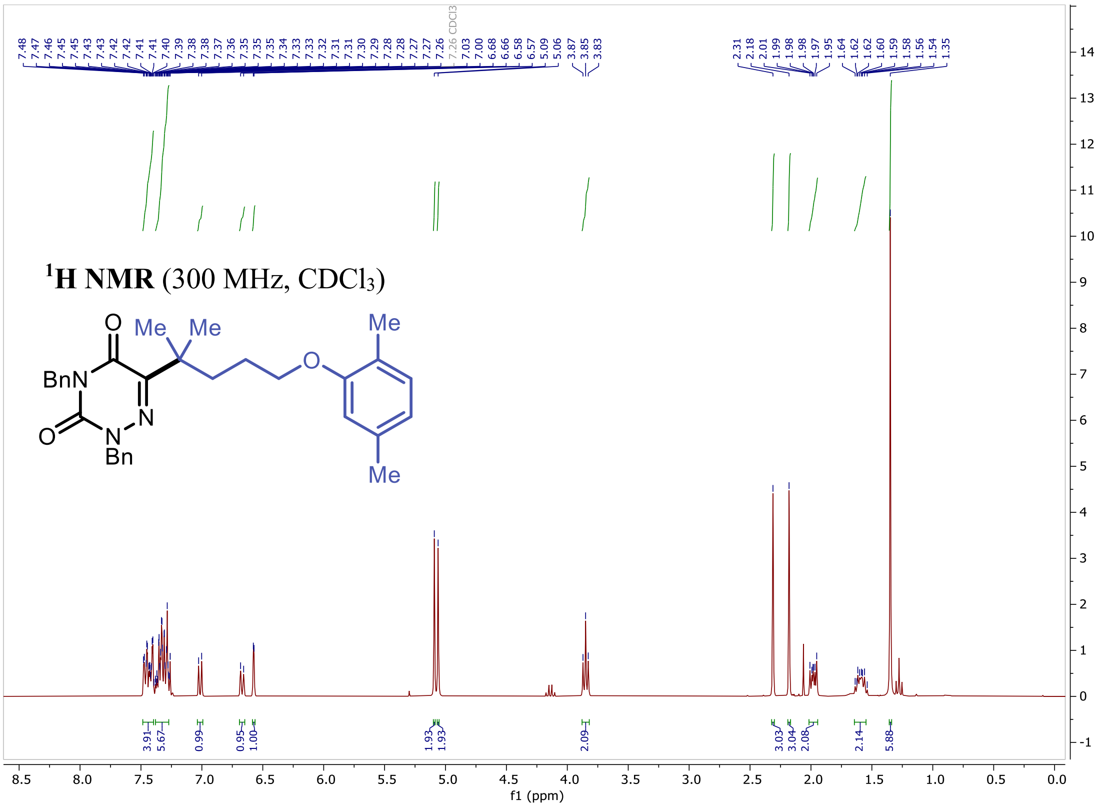

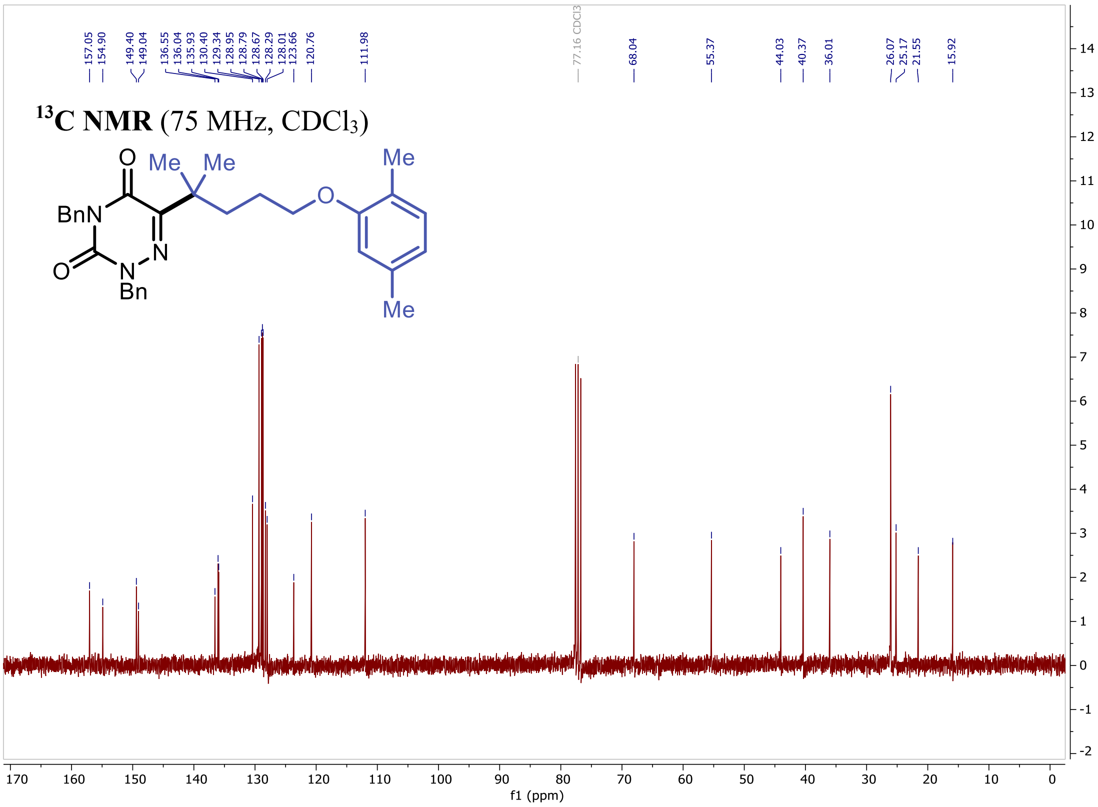

2,4-Dibenzyl-6-(1-methylcyclohexyl)-1,2,4-triazine-3,5(2*H*,4*H*)-dione (3d)

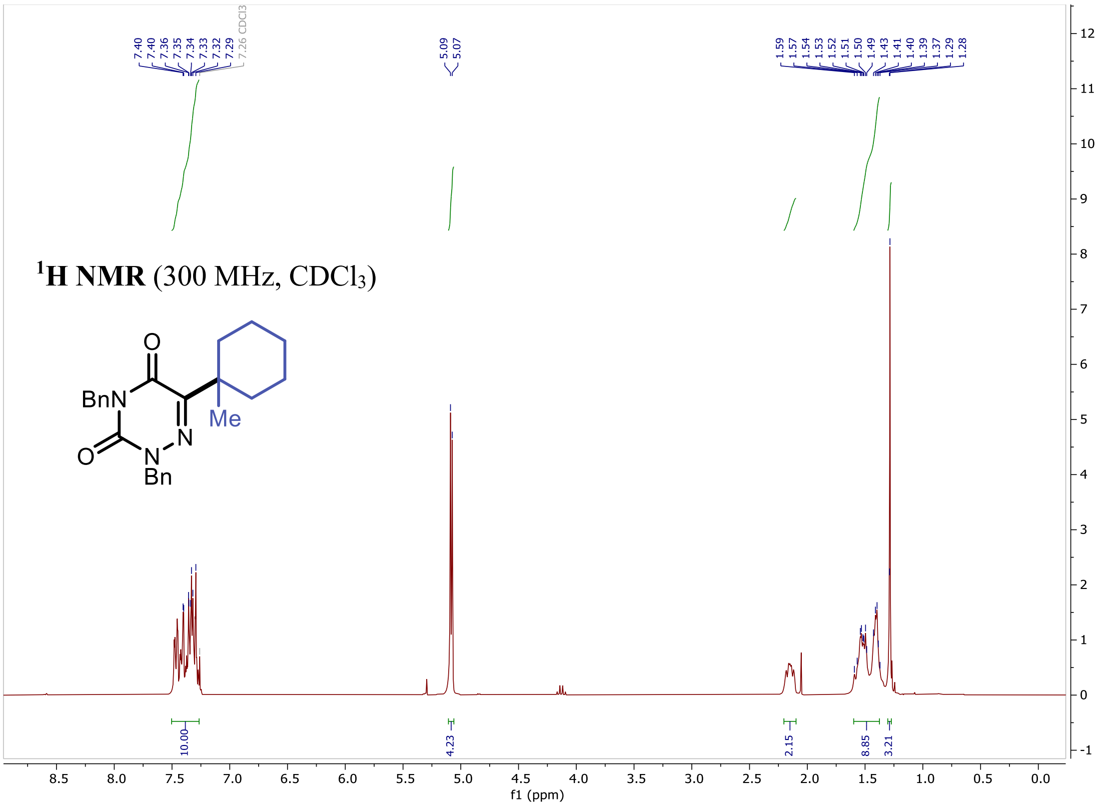

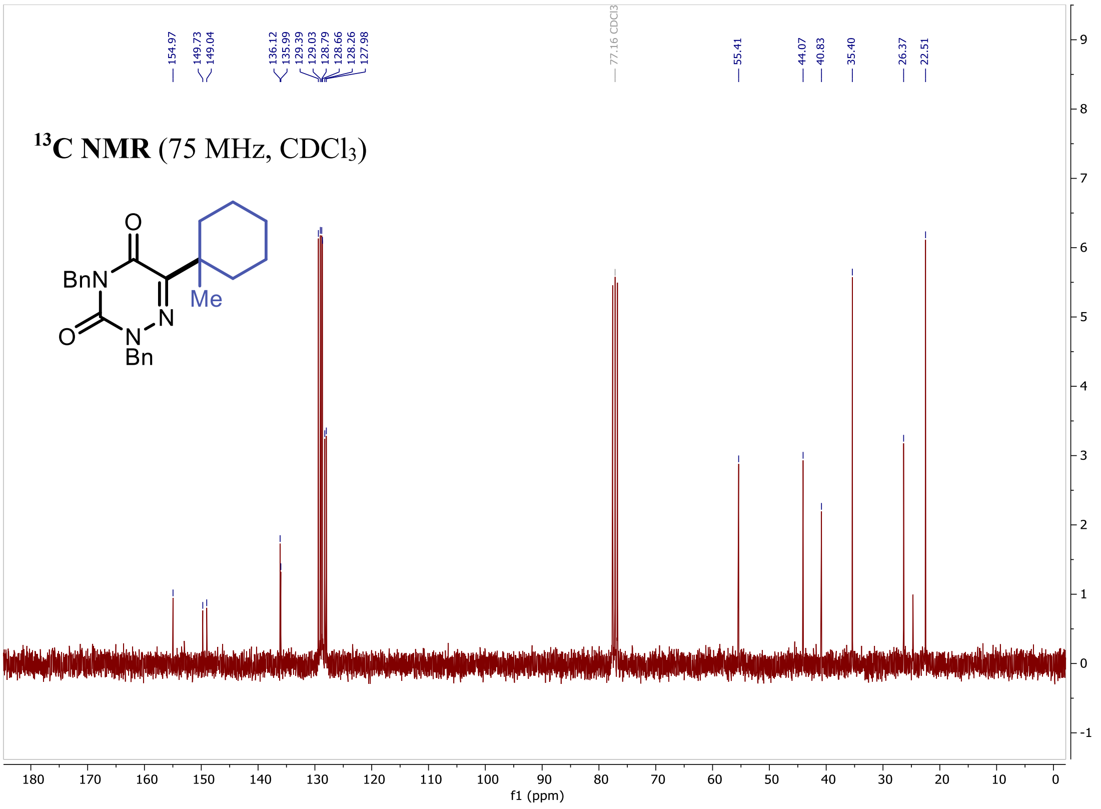

***tert*-Butyl-2-(2,4-dibenzyl-3,5-dioxo-2,3,4,5-tetrahydro-1,2,4-triazin-6-yl) pyrrolidine-1-carboxylate (3e)** – some signals are broad and split due to the presence of diastereomeric rotamers.

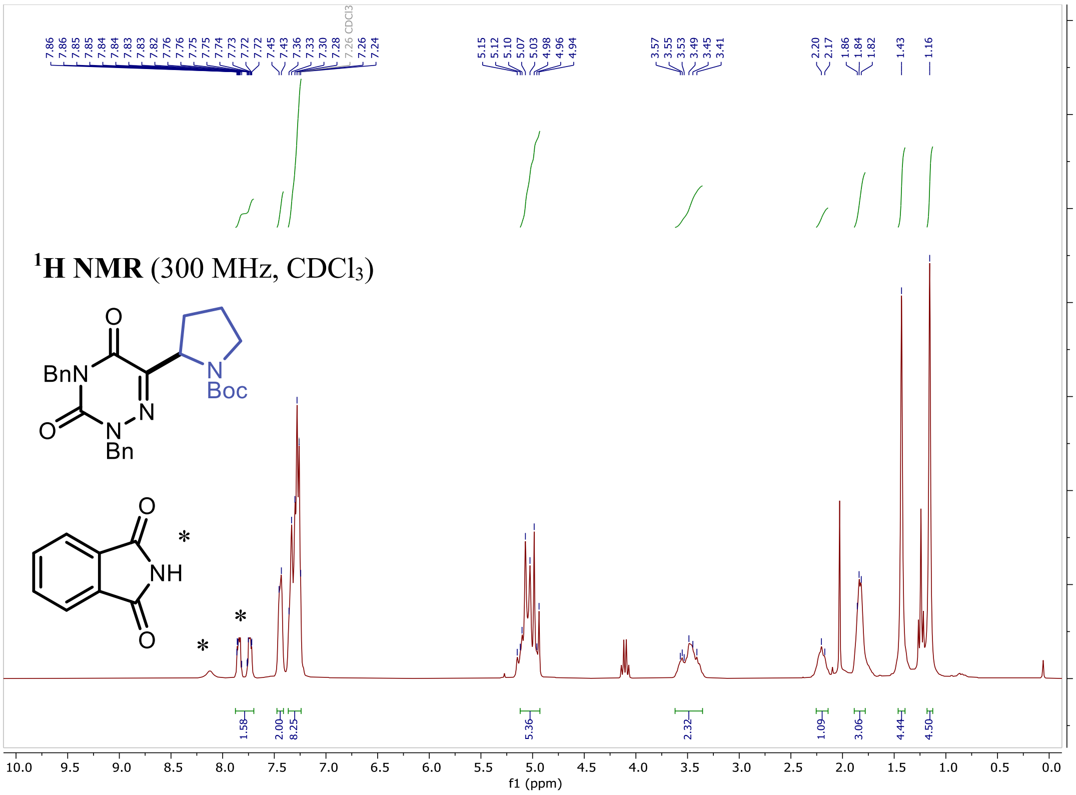

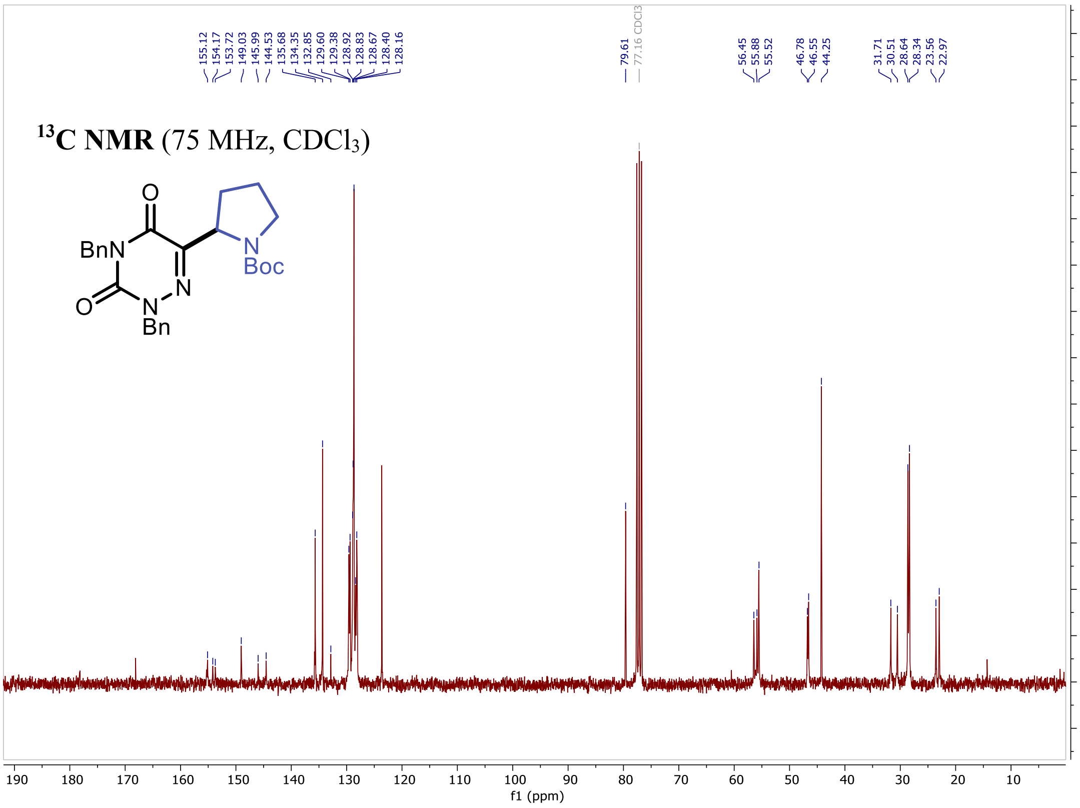

*Together with a small amount of inseparable phthalimide; yield was corrected accordingly using an internal standard.

2,4-Dibenzyl-6-cyclohexyl-1,2,4-triazine-3,5(2*H*,4*H*)-dione (3f)

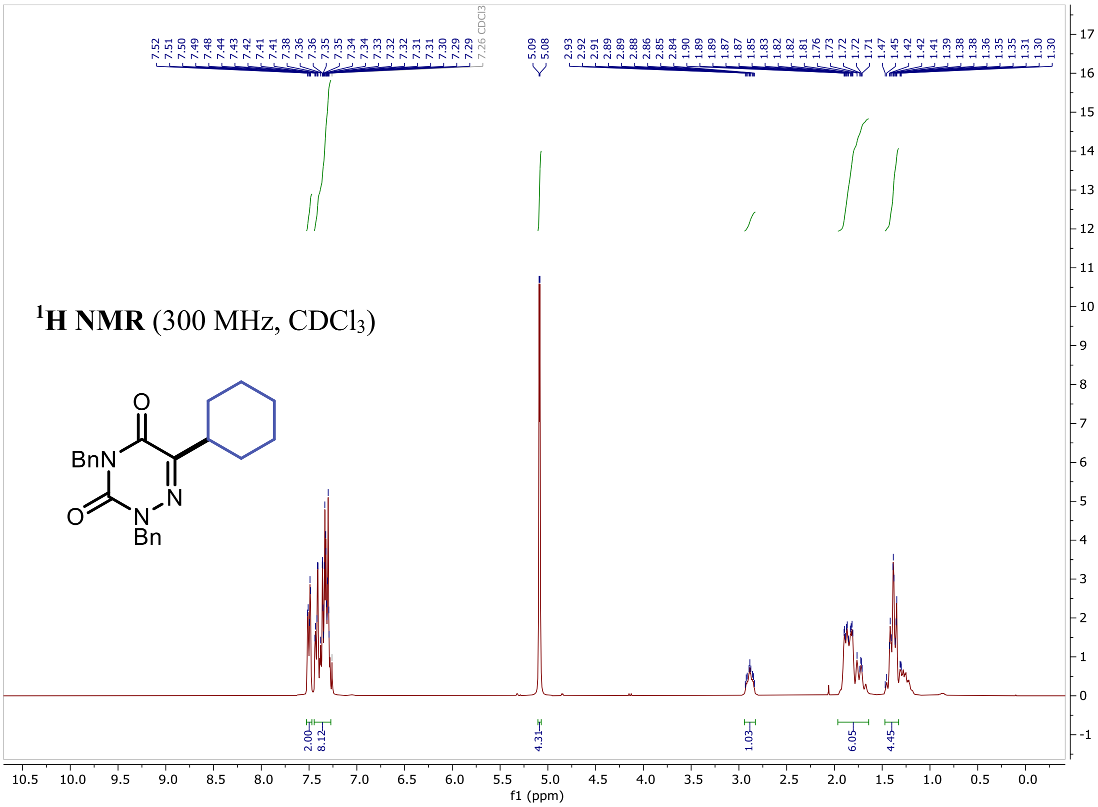

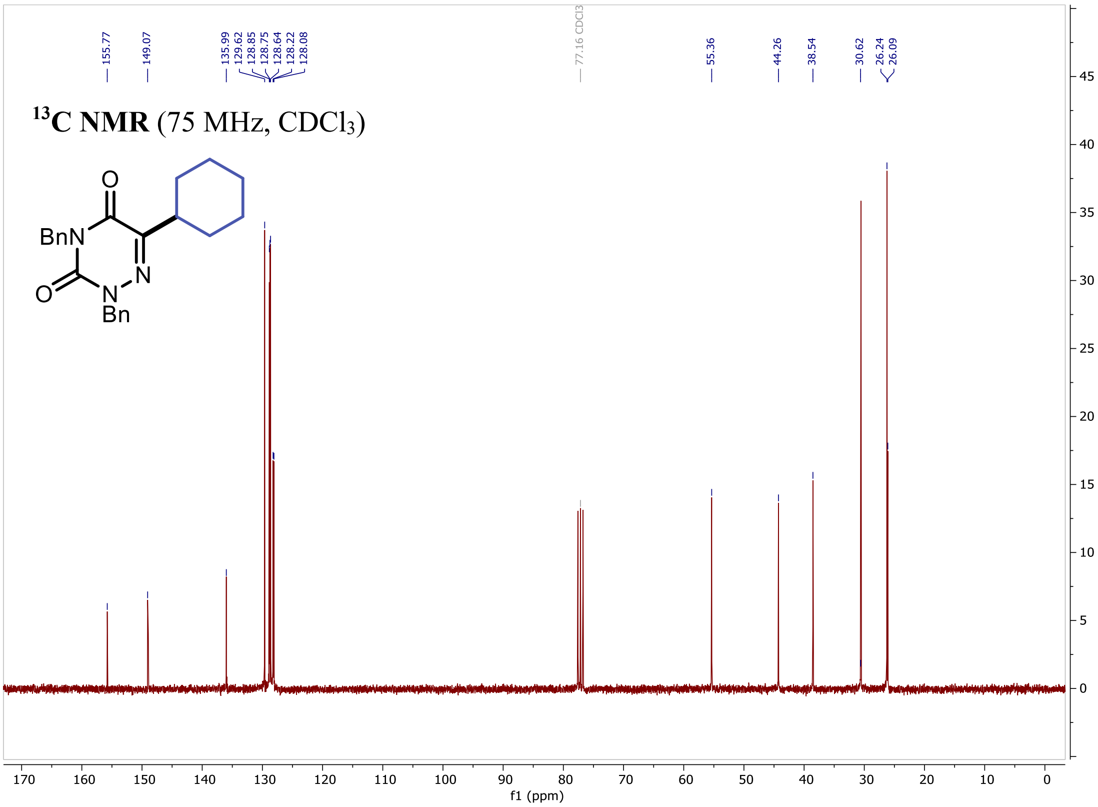

2,4-Dibenzyl-6-(5-oxo-5-phenylpentyl)-1,2,4-triazine-3,5(2*H*,4*H*)-dione (3g)

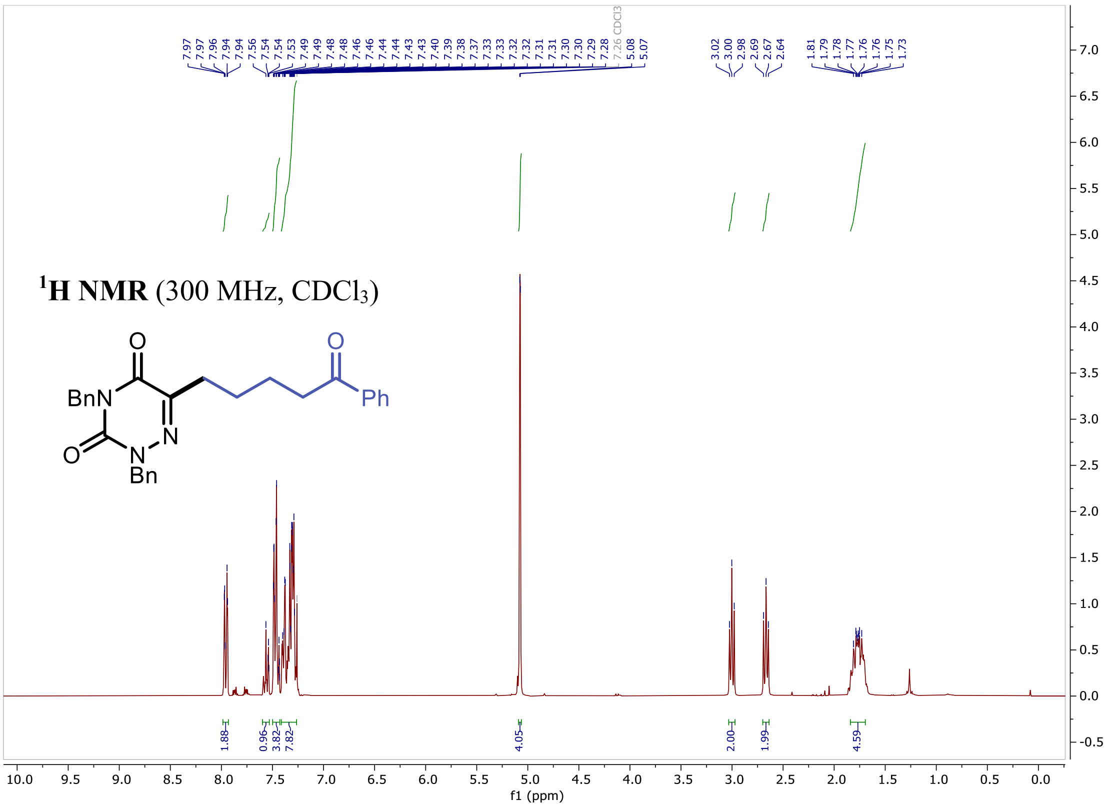

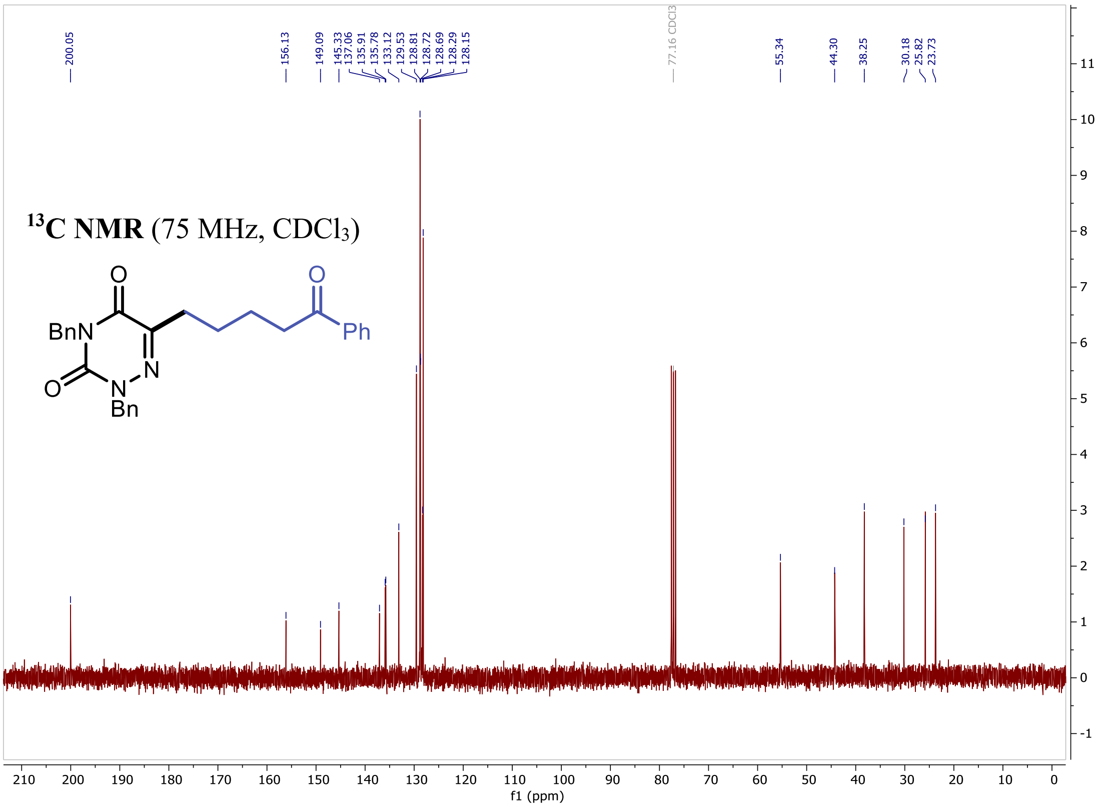

2,4-Dibenzyl-6-(3,4-dimethoxyphenethyl)-1,2,4-triazine-3,5(2*H*,4*H*)-dione (3h)

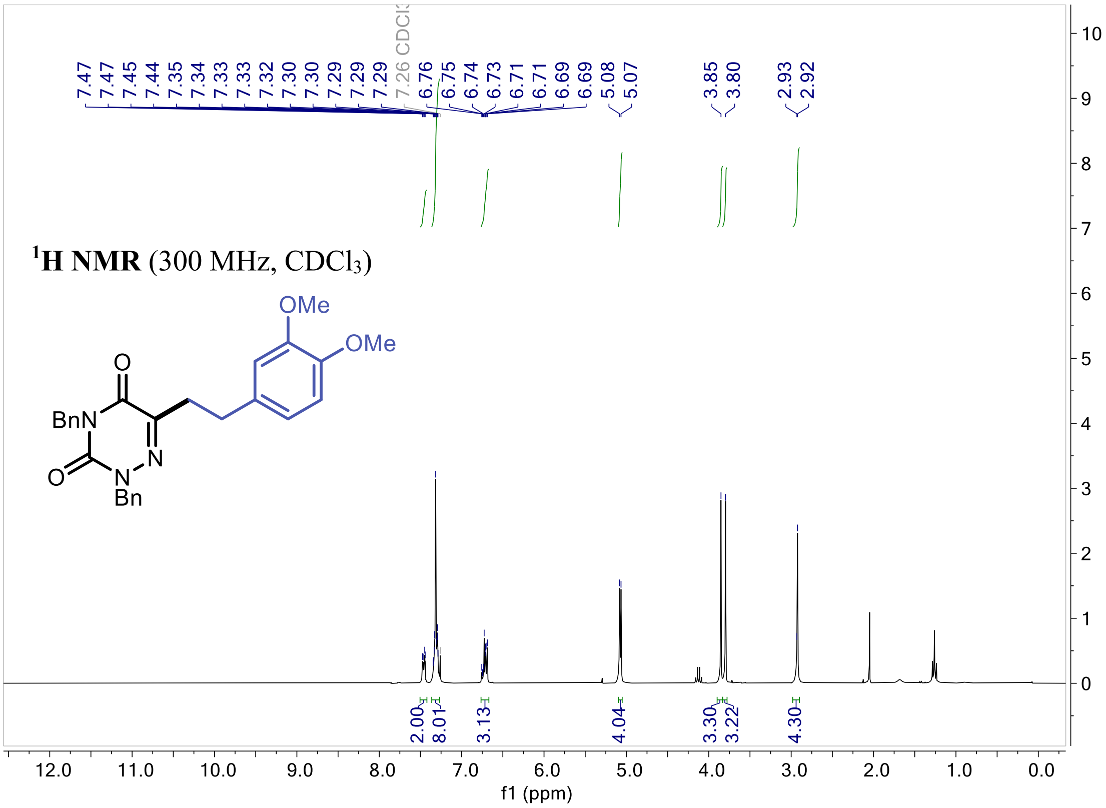

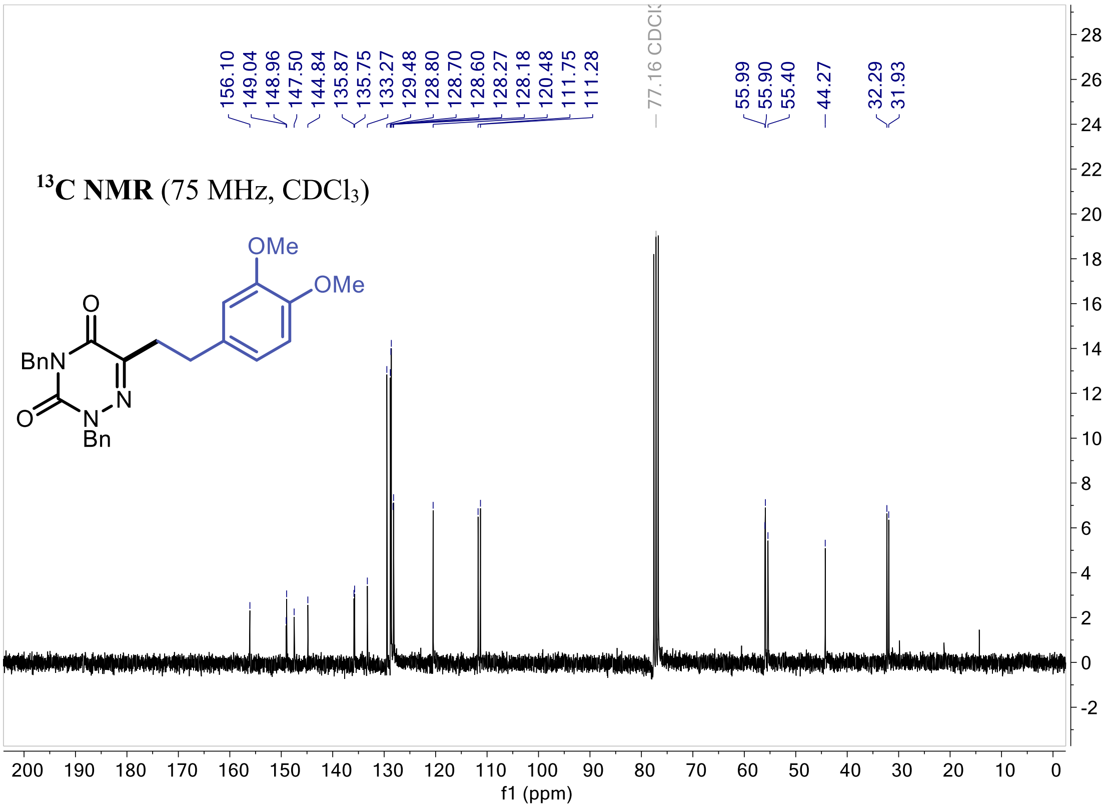

6-(*tert*-Butyl)-2,4-dimethyl-1,2,4-triazine-3,5(2*H*,4*H*)-dione (3i)

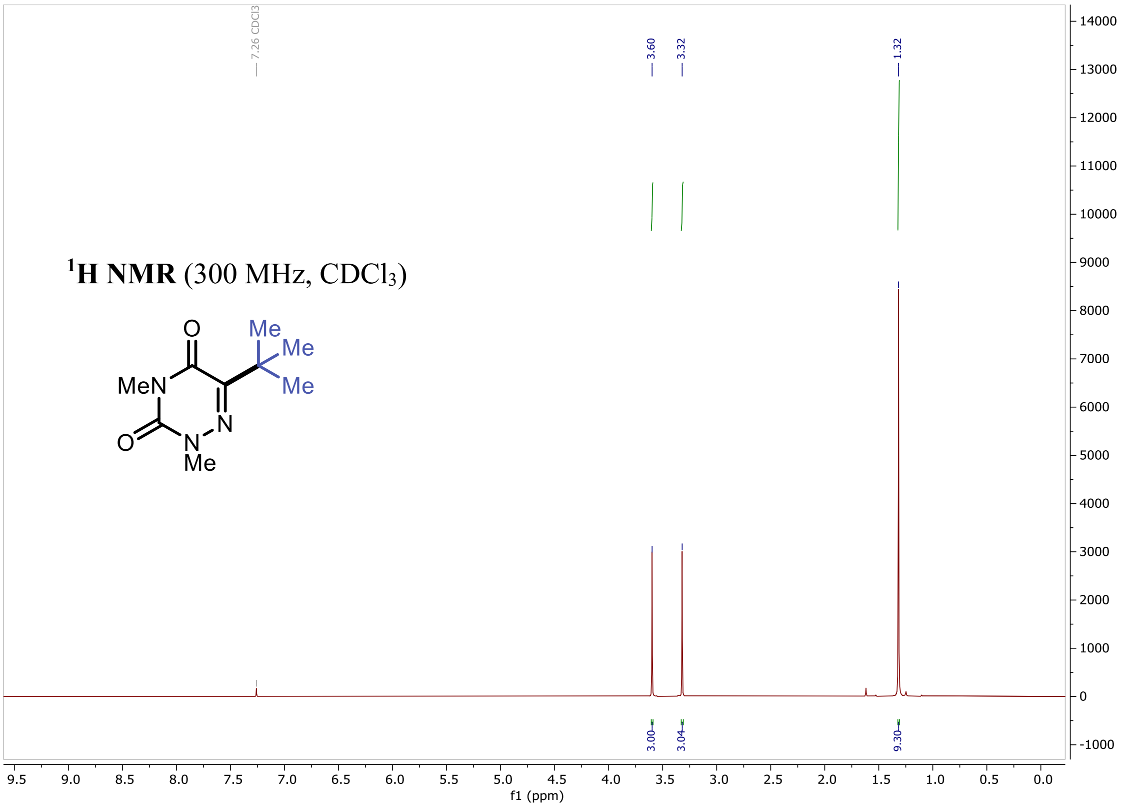

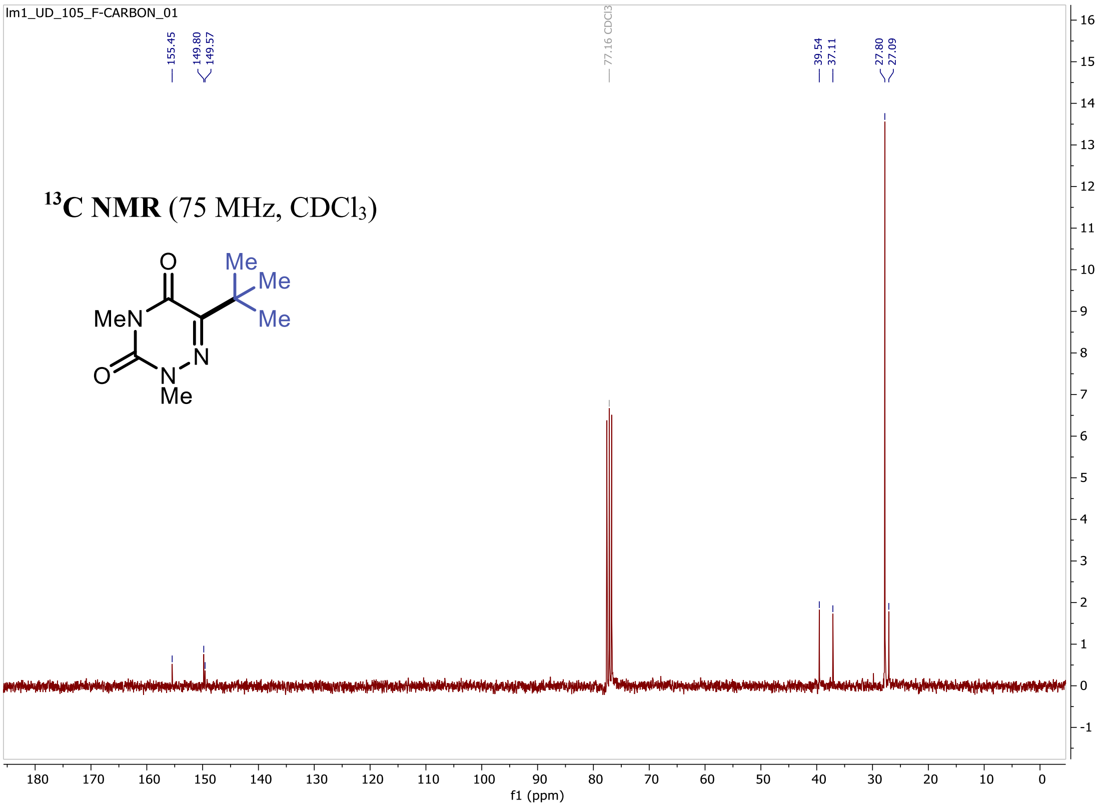

2-Benzyl-6-(tert-butyl)-1,2,4-triazine-3,5(2*H*,4*H*)-dione (3j)

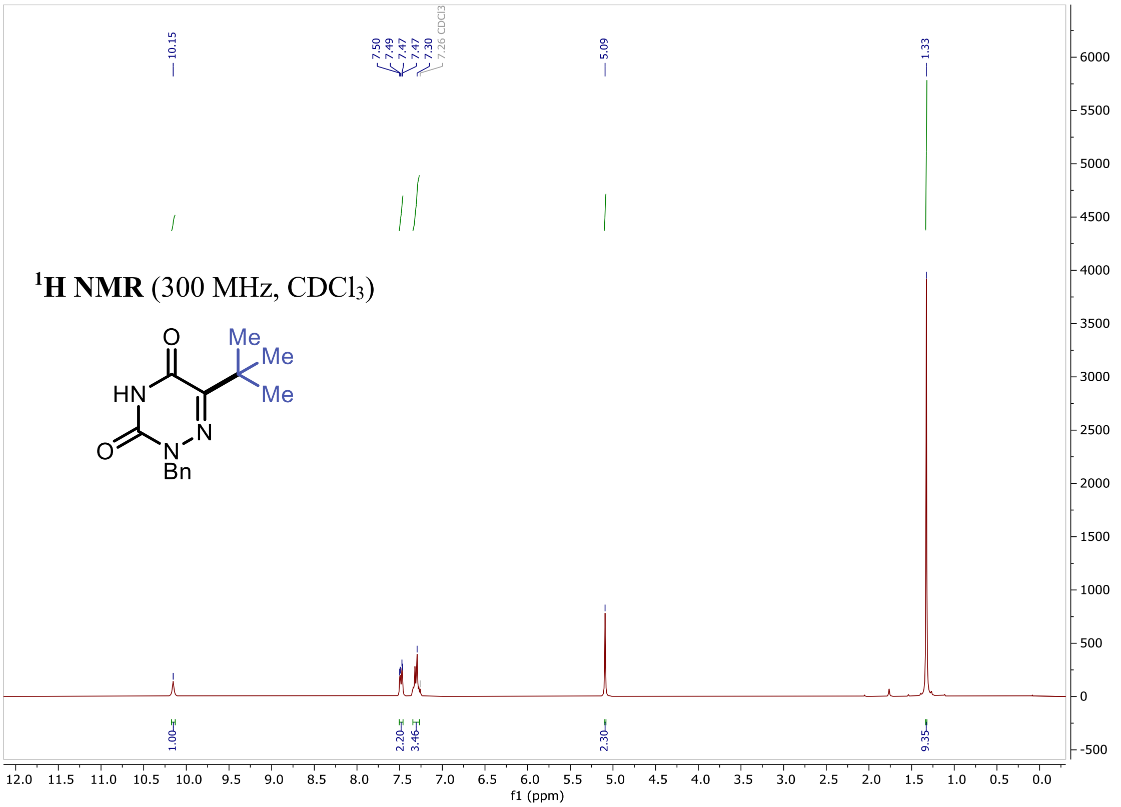

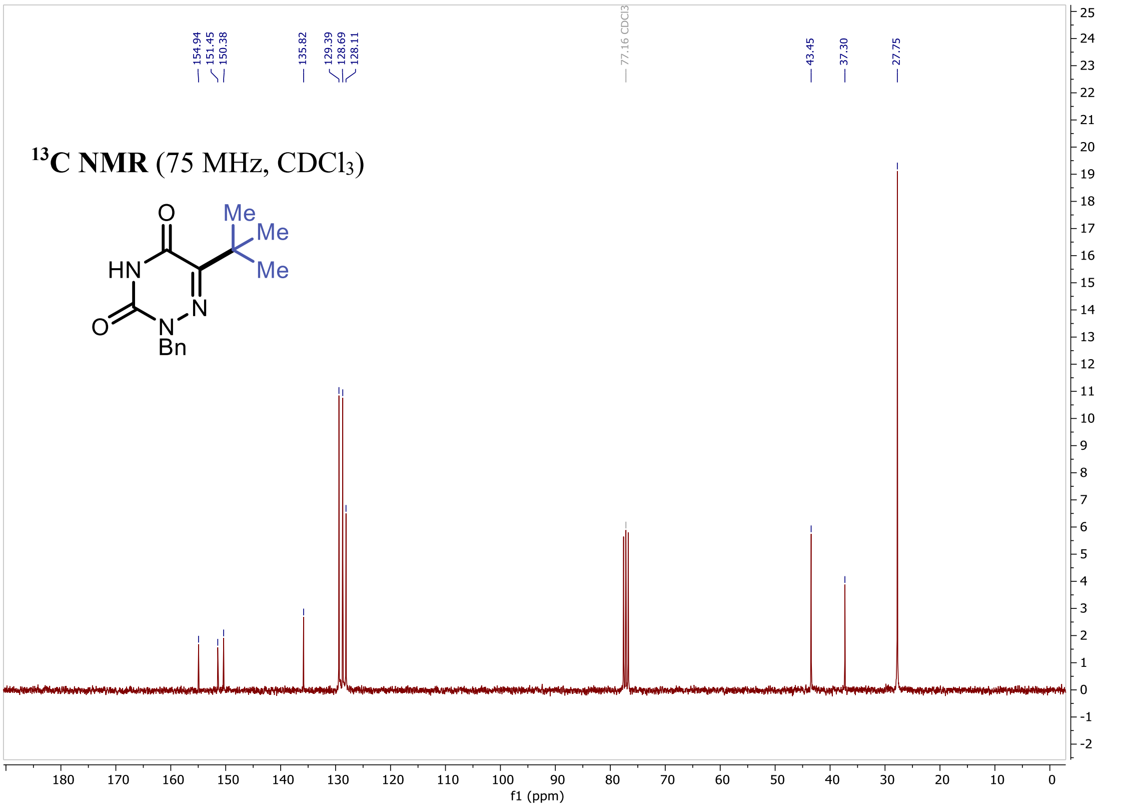

Ethyl 2-(2-benzyl-6-(tert-butyl)-3,5-dioxo-2,5-dihydro-1,2,4-triazin-4(3*H*)-yl) acetate (3k)

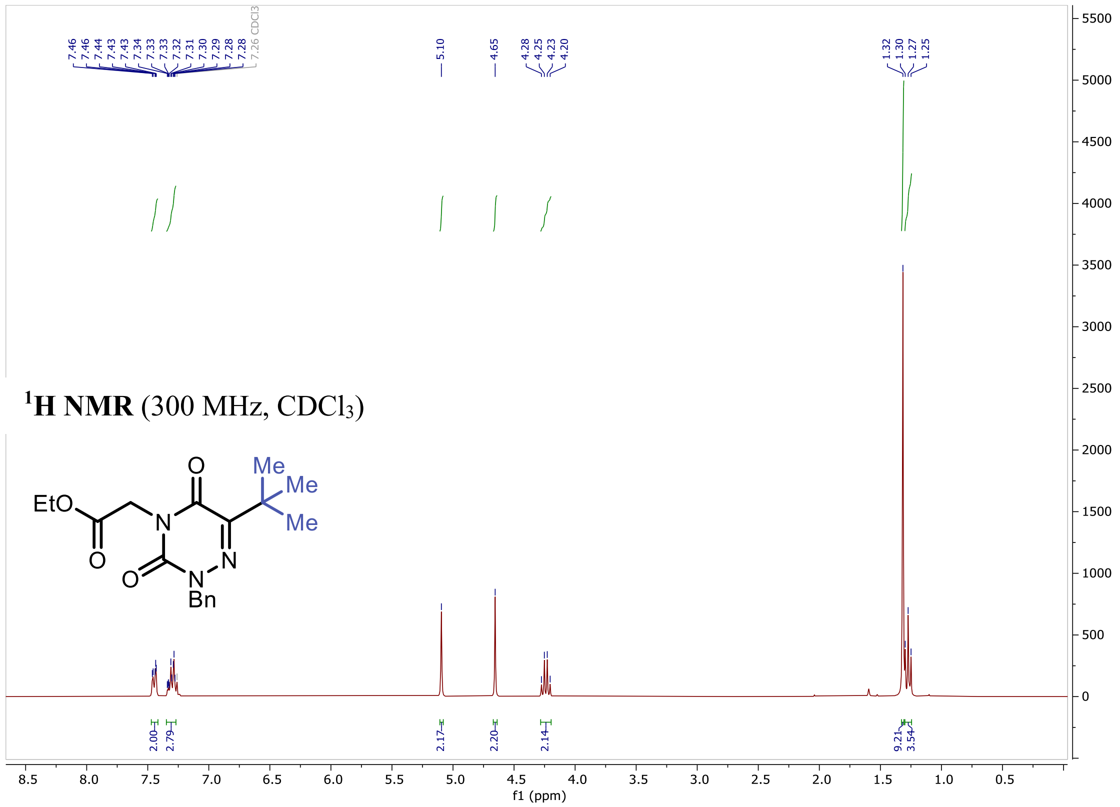

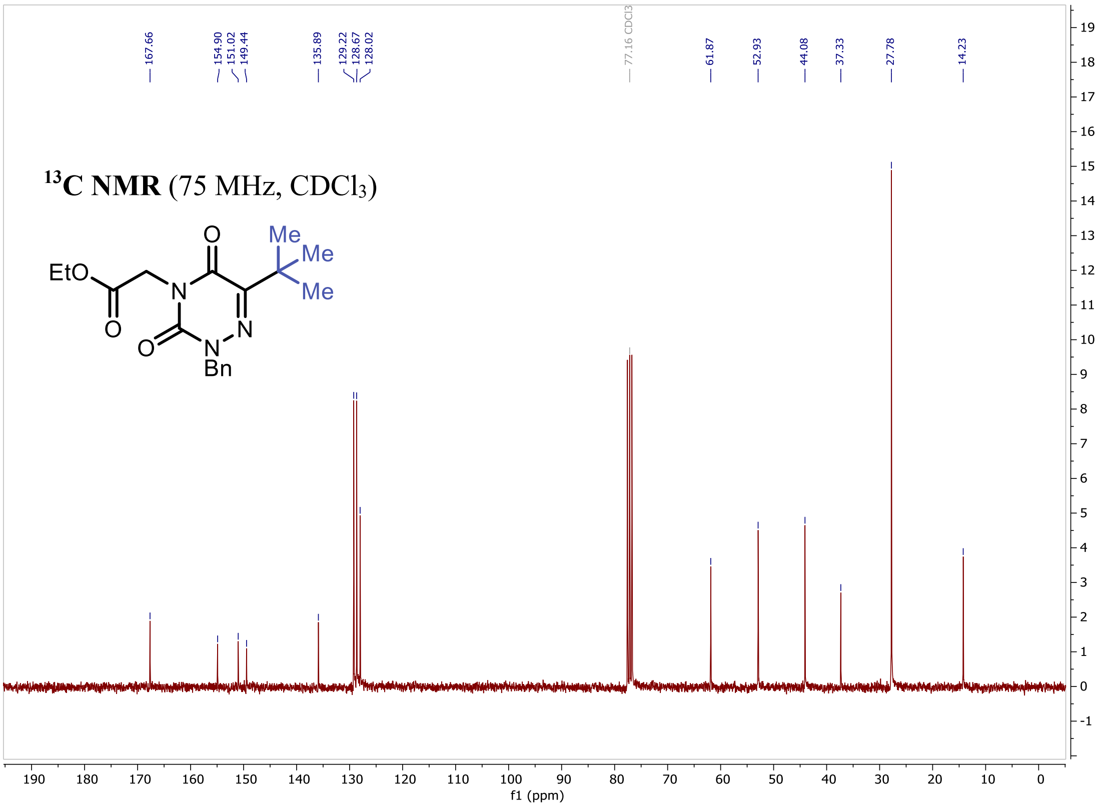

2-Benzyl-6-(tert-butyl)-4-(prop-2-yn-1-yl)-1,2,4-triazine-3,5(2*H*,4*H*)-dione (3l)

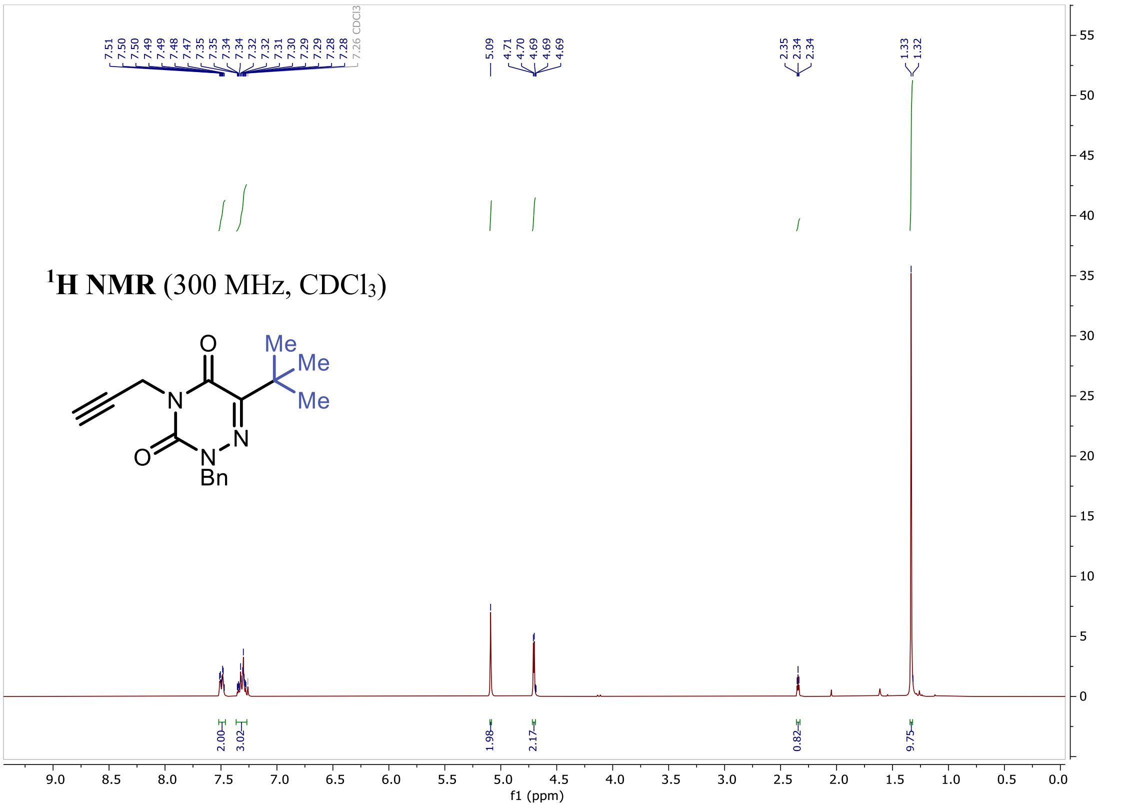

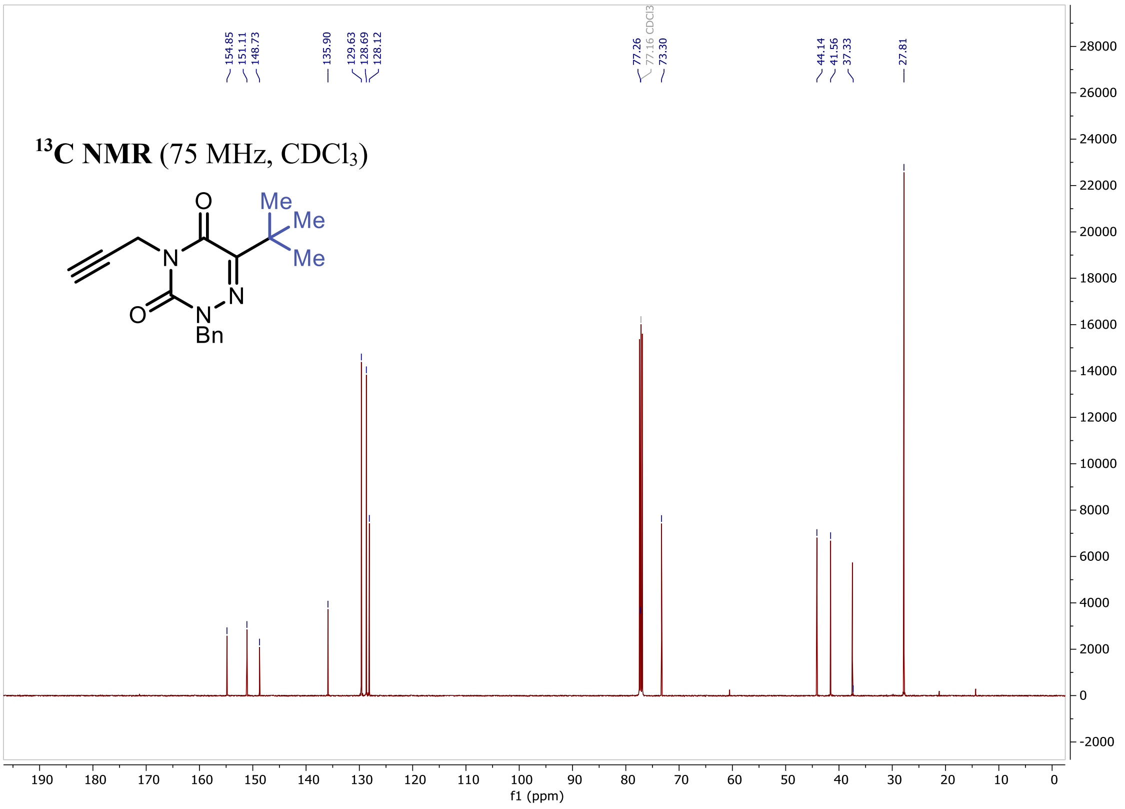

3-(*tert*-Butyl)-2-phenyl-2*H*-indazole (3n)

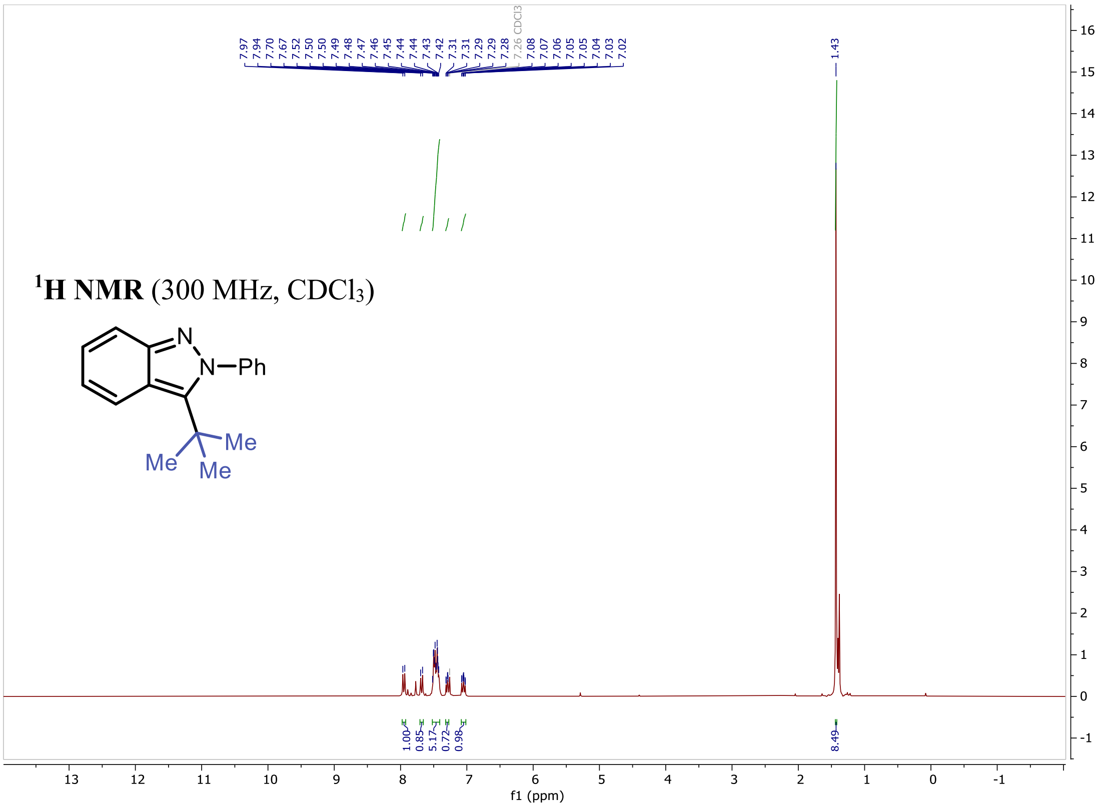

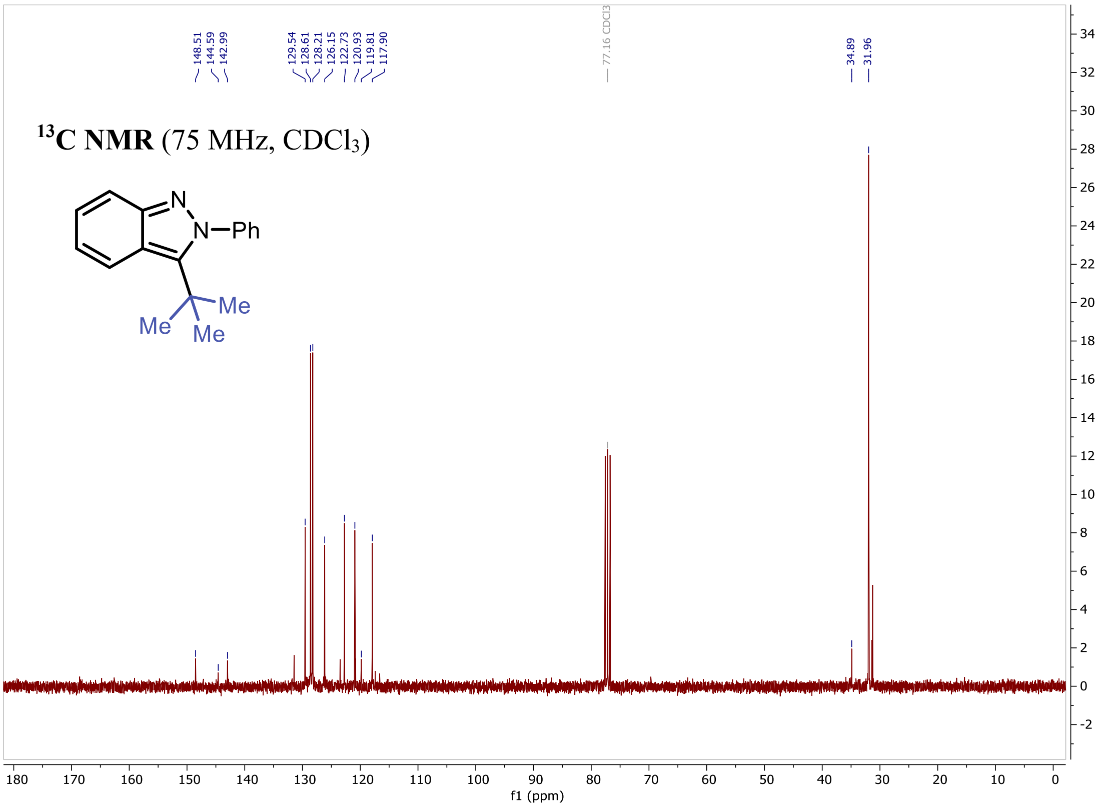

*Together with a small inseparable impurity; yield was corrected accordingly using an internal standard.

2-(*tert*-Butyl) quinoxaline (3o)

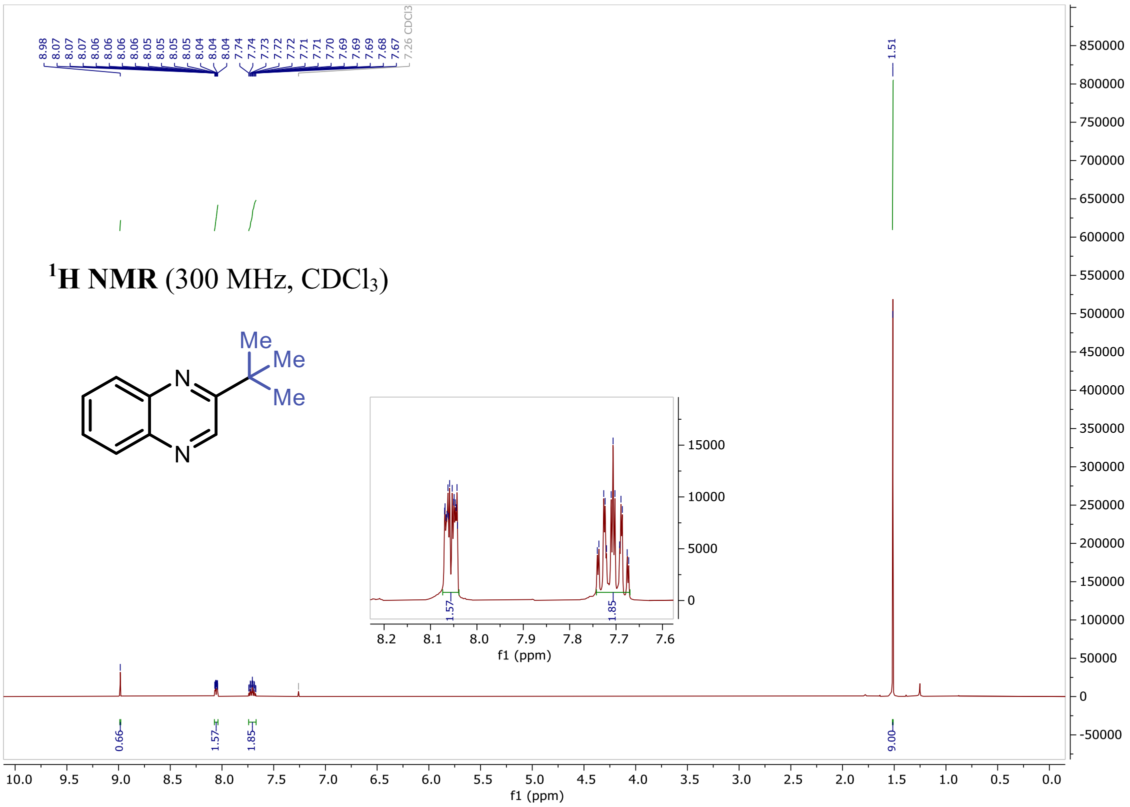

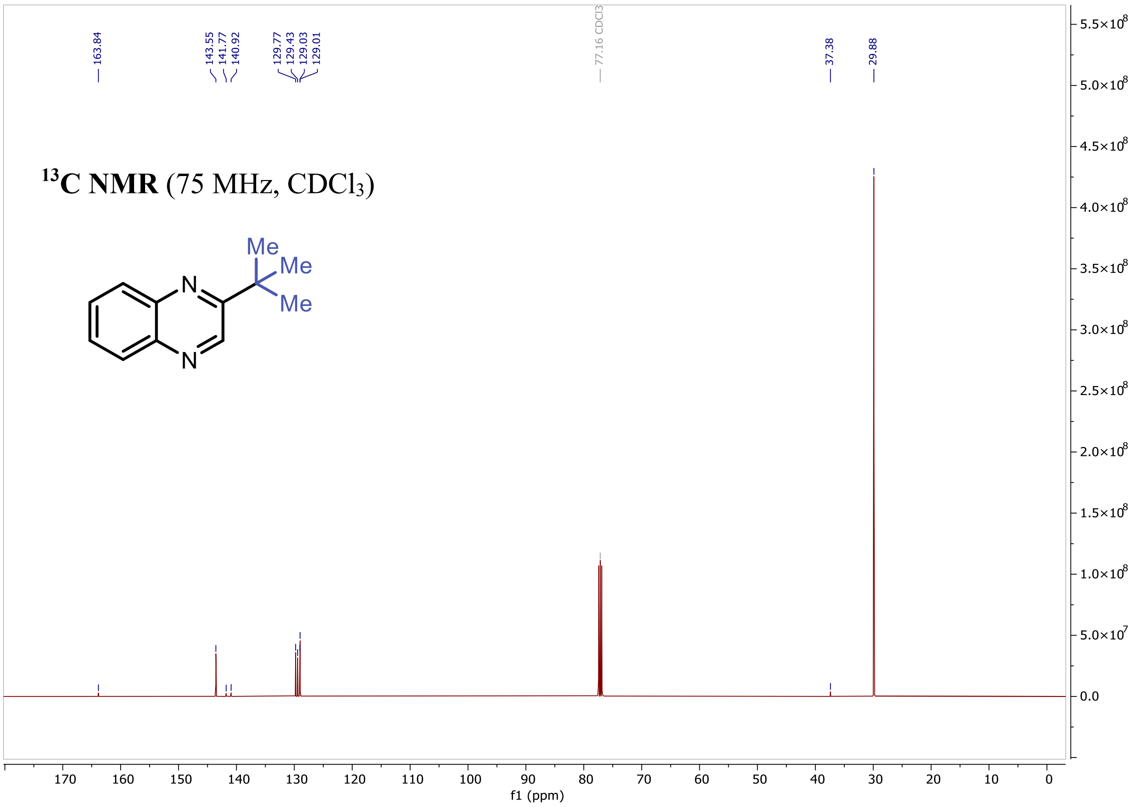

1-Benzyl-3-(*tert*-butyl) quinoxalin-2(1*H*)-one (3p)

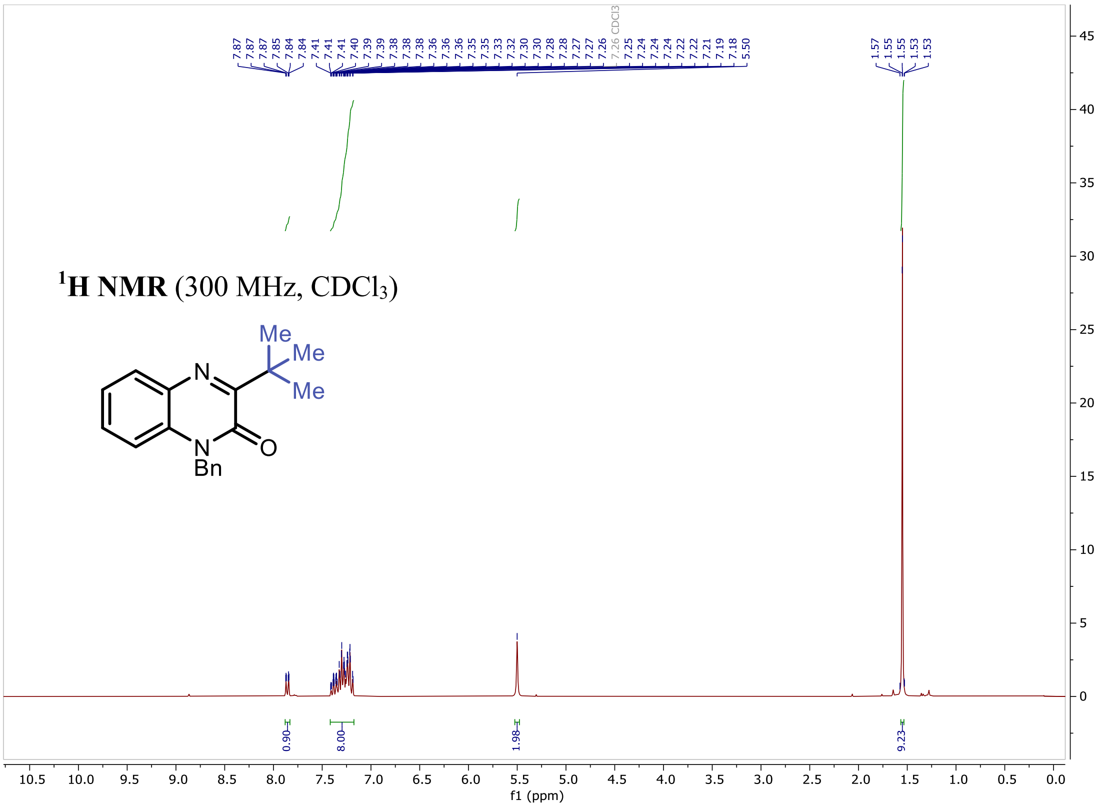

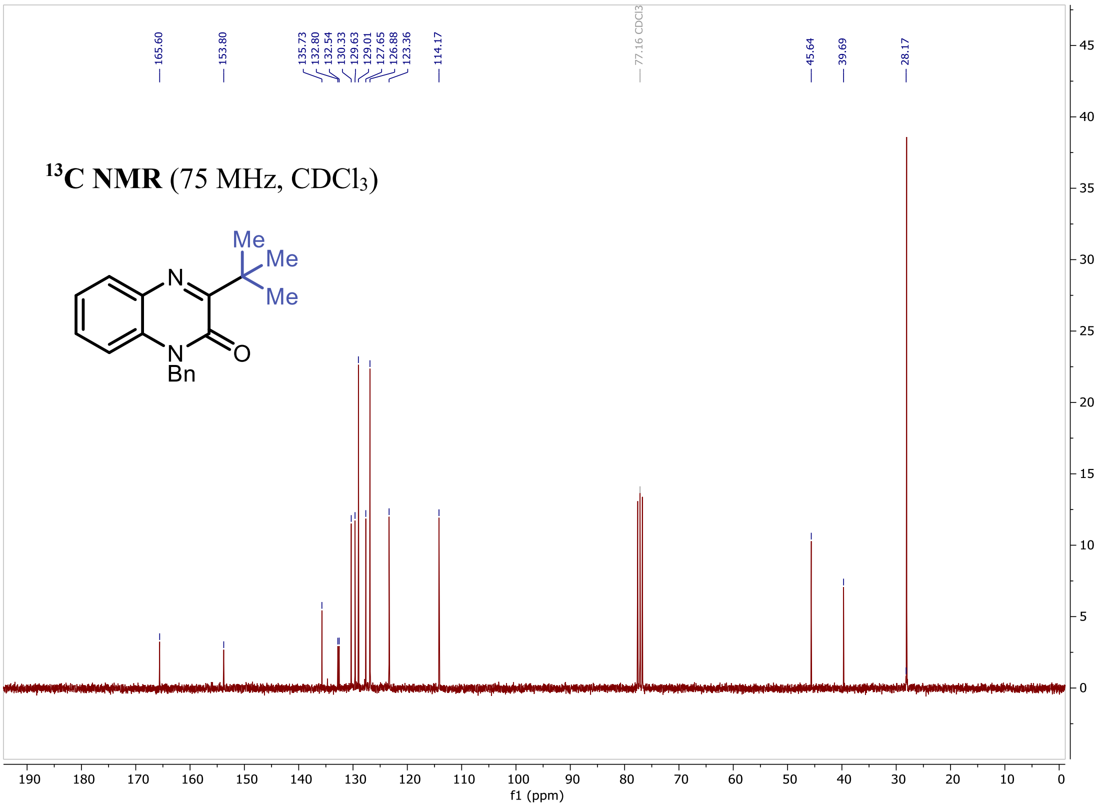

3-(tert-butyl)-4*H*-chromen-4-one (3q)

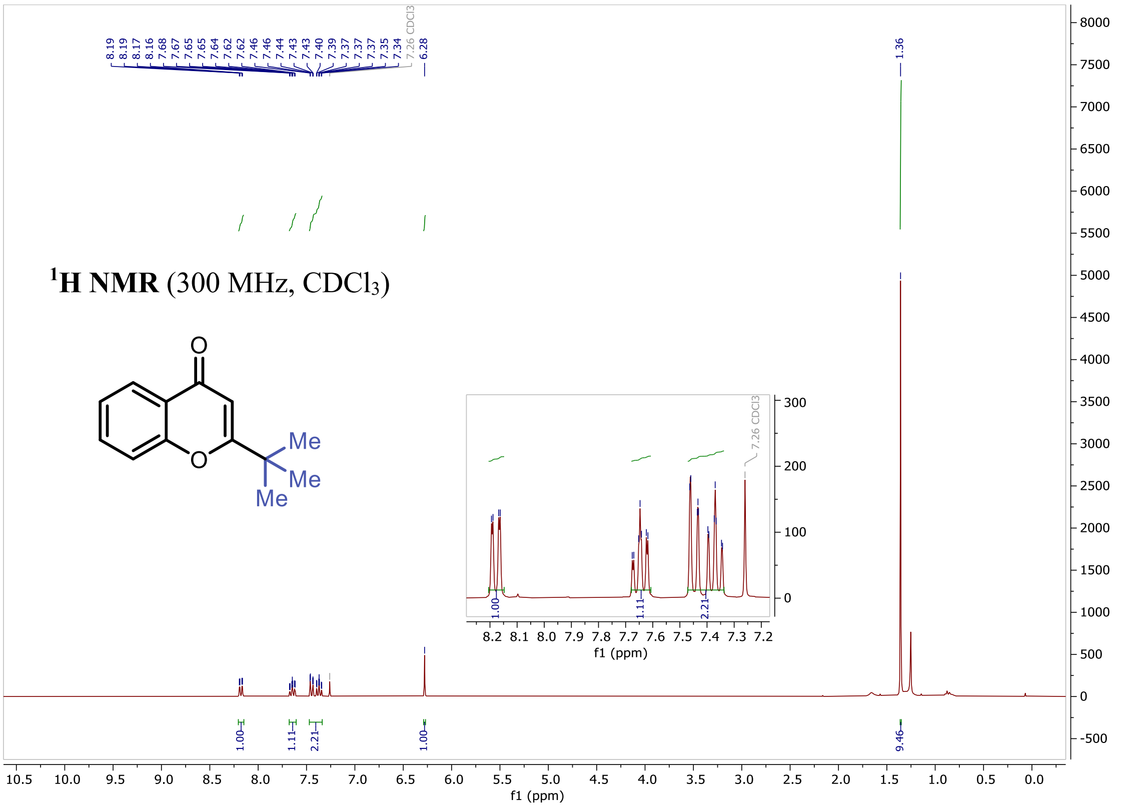

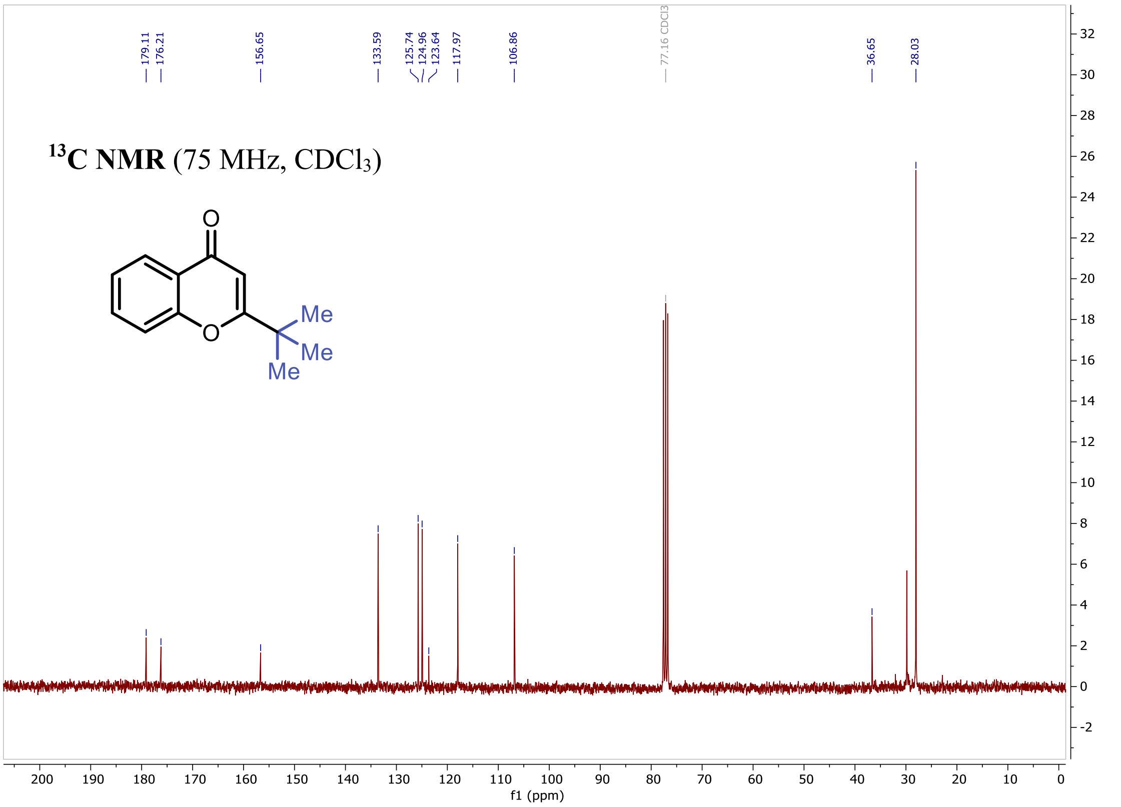

12. References

1. (a) Toriyama, F.; Cornella, J.; Wimmer, L.; Chen, T.-G.; Dixon, D. D.; Creech, G.; Baran, P. S. *J. Am Chem. Soc.* **2016**, *138*, 11132–11135. (b) Sherwood, T. C.; Xiao, H.-Y.; Bhaskar, R. G.; Simmons, E. M.; Zaretsky, S.; Rauch, M. P.; Knowles R. R.; Murali Dahr, T. G. *J. Org. Chem.* **2019**, *84*, 8360–8379. (c) Chowdhury, R.; Yu, Z.; Tong, M. L.; Kohlhepp, S. V.; Yin, X.; Mendoza, A. *J. Am. Chem. Soc.* **2020**, *142*, 20143–20151. (d) Montoto, D.; Deus-Lorenzo, U.; Tomás-Gamasa, M.; Mascareñas, J. L.; Mato, M. *Org. Biomol. Chem.* **2025**, *23*, 9833-9838.
2. (a) Chen, D.; Wang, Z. J.; Bao, W. *J. Org. Chem.* **2010**, *75*, 5768–5771. (b) Panda, S. P.; Hota, S. K.; Dash, R.; Roy L.; Murarka, S. *Org. Lett.* **2023**, *25*, 3739−3744. (c) Zhu, J.; Hong, Y.; Wang, Y.; Guo, Y.; Zhang, Y.; Ni, Z.; Li, W.; Xu, J. *ACS Catal.* **2024**, *14*, 6247–6258. (d) Wang, M.; Wang, Y.; Wang, J.; Zhu, Y.; Zhang, P.; Zhang, C.; Chen, J.; Guo, L.; Lv, G.; Wu, Y. *J. Org. Chem.* **2025**, *90*, 7049–7061.
3. (a) Tait, M. A.; Hik, D. S. *Photosynth. Res.* **2003**, *78*, 87–91. (b) Shinano, T.; Lei, T. T.; Kawamukai, T.; Inoue, M. T.; Koike, T.; Tadano, T. *Photosynthetica* **1996**, *32*, 409–415.
4. van Stokkum, I. H. M.; Larsen, D. S.; van Grondelle, R. *Biochim. Biophys. Acta* **2016**, *1857*, 1627–1640.
5. (a) Mullen, K. M.; van Stokkum, I. H. M. *J. Stat. Sowftw.* **2007**, *18*, 1–48. (b) Snellenburg, J. J.; Laptenok, S.; Seger, R.; Mullen, K. M.; van Stokkum, I. H. M. *J. Stat. Softw.* **2012**, *49*, 1–22.
6. Hanna, L.; Movsesian, E.; Orozco, M.; Bernot Jr., A. R.; Asadinamin, M.; Shenje, L.; Ullrich, S.; Zhao, Y.; Marshall, N.; Weeks, J. A.; Thomas, M. B.; Teprovich Jr., J. A.; Ward, P. A. *Spectrochim Acta A Mol Biomol Spectrosc.* **2022**, *278*, 121300.
7. Fukuzumi, S.; Inada, O.; Suenobu, T. *J. Am. Chem. Soc.* **2003**, *125*, 4808–4816.
8. (a) Dance, Z. E. X.; Mi, Q.; McCamant, D. W.; Ahrens, M. J.; Ratner, M. A.; Wasielewski, M. R. *J. Phys. Chem. B* **2006**, *110*, 25163–25173. (b) Wasielewski, M. R. *J. Org. Chem.* **2006**, *71*, 5051–5066. (c) Liu, M.; Zhu, J.; Zhao, G.; Li, Y.; Yang, Y.; Gao, K.; Wu, K. *Nat. Mater.* **2025**, *24*, 260–267.
